# Leveraging artificial intelligence for pandemic preparedness and response: a scoping review to identify key use cases

**DOI:** 10.1038/s41746-021-00459-8

**Published:** 2021-06-10

**Authors:** Ania Syrowatka, Masha Kuznetsova, Ava Alsubai, Adam L. Beckman, Paul A. Bain, Kelly Jean Thomas Craig, Jianying Hu, Gretchen Purcell Jackson, Kyu Rhee, David W. Bates

**Affiliations:** 1 Division of General Internal Medicine, Brigham and Women’s Hospital, Boston, MA USA; 2 Harvard Medical School, Boston, MA USA; 3 Harvard Business School, Boston, MA USA; 4 Countway Library of Medicine, Harvard Medical School, Boston, MA USA; 5 IBM Watson Health, Cambridge, MA USA; 6 IBM Research, Center for Computational Health, Yorktown Heights, NY USA; 7 Department of Pediatric Surgery, Vanderbilt University Medical Center, Nashville, TN USA; 8 CVS Health, Wellesley Hills, MA USA; 9 Harvard T. H. Chan School of Public Health, Boston, MA USA

**Keywords:** Viral infection, Health policy, Epidemiology

## Abstract

Artificial intelligence (AI) represents a valuable tool that could be widely used to inform clinical and public health decision-making to effectively manage the impacts of a pandemic. The objective of this scoping review was to identify the key use cases for involving AI for pandemic preparedness and response from the peer-reviewed, preprint, and grey literature. The data synthesis had two parts: an in-depth review of studies that leveraged machine learning (ML) techniques and a limited review of studies that applied traditional modeling approaches. ML applications from the in-depth review were categorized into use cases related to public health and clinical practice, and narratively synthesized. One hundred eighty-three articles met the inclusion criteria for the in-depth review. Six key use cases were identified: forecasting infectious disease dynamics and effects of interventions; surveillance and outbreak detection; real-time monitoring of adherence to public health recommendations; real-time detection of influenza-like illness; triage and timely diagnosis of infections; and prognosis of illness and response to treatment. Data sources and types of ML that were useful varied by use case. The search identified 1167 articles that reported on traditional modeling approaches, which highlighted additional areas where ML could be leveraged for improving the accuracy of estimations or projections. Important ML-based solutions have been developed in response to pandemics, and particularly for COVID-19 but few were optimized for practical application early in the pandemic. These findings can support policymakers, clinicians, and other stakeholders in prioritizing research and development to support operationalization of AI for future pandemics.

## Introduction

Given the pace of globalization, future pandemics are likely to follow novel coronavirus disease 2019 (COVID-19), although their frequency is uncertain. Half a year into the pandemic, it was estimated that 59–92% of COVID-19 deaths in the USA could have been avoided if the pandemic had been managed differently and mortality rates were similar to those in countries with moderate rates of COVID-19 deaths, such as Norway or Canada^1^.

Despite a significantly lower mortality rate compared with severe acute respiratory syndrome (SARS), caused by a related coronavirus (SARS-CoV) with a case fatality rate of 11%^2^, COVID-19 has resulted in exponentially more harm. The virus spread rapidly and widely around the world, in a way SARS-CoV did not, from asymptomatic and mild cases resulting in undetected spread and leading to a higher number of deaths overall. If pandemics are to be managed effectively, policymakers, clinicians, and other stakeholders need access to data and recommendations in near-real time, including models to weigh the relative risks and benefits of various interventions. Notably, there have been numerous conflicting projection models for COVID-19, but few were accurate for this novel pathogen.

Policymakers and governments have many choices for population-level health interventions, which are critical to control spread early on. Non-pharmaceutical interventions include implementing travel bans, closing businesses, shutting schools, mandating masks, and allocating scarce supplies such as personal protective equipment (PPE) and testing. Implementation, timing, enforcement, and cessation all represent additional choices. Many of these decisions are still based on expert recommendations, rather than data-driven models. With these decisions come difficult tradeoffs, as many have serious economic consequences as well as direct health implications. For example, implementing restrictions (e.g., stay-at-home orders) during a pandemic may reduce infection-related morbidity and mortality, but the associated economic decline, social isolation, and delayed medical care also adversely affect public health and welfare.

Optimally managing a pandemic necessitates rapid feedback cycles of data-driven learning to respond effectively at each step. Policymakers must make initial decisions about which interventions are most likely to protect public health, and make mid-course adjustments, including updating policies and recommendations as more data become available. Clinicians must determine how to diagnose, triage, and care for infected patients under uncertainty, given the possibility that the pathogen may behave differently from known infections; rapidly studying and disseminating information about symptoms, disease progression, and responses to treatments are critical for reducing harm.

Data have always been important for healthcare and public health decision-making; however, data have been especially instrumental in efforts to tackle COVID-19 worldwide. Unprecedented levels of global collaboration have initiated data-sharing efforts from traditional sources such as those from health services, and non-traditional ones including transportation records and personal data from smartphones. These early strides in data sharing are critical for artificial intelligence (AI) where performance improves with large, inclusive, historical and real-time datasets. Innovations are rapidly advancing the application of data, advanced analytics, and machine learning (ML) to help manage the COVID-19 pandemic.

The objective of this scoping review was to synthesize available literature describing the use of AI to inform clinical and public health decision-making for pandemic preparedness and response. This review had two parts: an in-depth review of studies that leveraged ML techniques, and a limited review of studies that applied traditional modeling approaches. The in-depth review identified key use cases for ML alongside data sources and types of ML well suited for each use case. The limited review highlighted additional areas where ML could be leveraged for improving the accuracy of estimations or projections.

## Methods

This scoping review is reported in accordance with the Preferred Reporting Items for Systematic Reviews and Meta-Analyses extension for Scoping Reviews (PRISMA-ScR)^3^.

### Search strategies

Five databases (PubMed [NCBI], Embase [Elsevier], Web of Science [Clarivate], IEEE Xplore [IEEE], and the ACM Guide to Computing Literature [ACM]) were searched without date limits on May 4, 2020, to identify relevant peer-reviewed literature. Two main concepts of AI and pandemics were mapped to the most relevant controlled vocabulary using Medical Subject Headings (MeSH), and free-text terms were included. Although the search strategy captured the published literature on all pandemics, additional MeSH terms and keywords were added to focus on the COVID-19 pandemic and the most recent past pandemic of influenza A subtype H1N1 (H1N1) in 2009. The search also captured relevant literature about the SARS global outbreak caused by SARS-CoV in 2003. Two preprint servers (medRxiv and bioRxiv) were searched from January 1 to May 27, 2020, to locate relevant research that had not yet been published. The main concepts of AI and COVID-19 were captured using free-text terms. Reference lists of included structured reviews were hand searched to identify further relevant studies.

In addition, a structured Google search was conducted to locate grey literature describing the application of AI for the management of COVID-19. Reputable trade and commercial publications were also reviewed to identify emerging and proprietary AI solutions. Peer-reviewed, preprint, and grey literature search strategies are provided in Supplementary Notes1–3.

### Inclusion and exclusion criteria

This scoping review had two parts: an in-depth review focused on the use of ‘complex’ ML for preparedness or response to viral respiratory pandemics as well as the SARS global outbreak, and a limited review describing the use of traditional modeling approaches. ‘Complex’ ML (hereafter referred to as ML) included neural networks, tree-based algorithms, support vector machines, and natural language processing. Traditional approaches included compartmental, simulation, statistical, and time series models. The *Glossary* provides a detailed listing of complex and traditional models (Box 1). Although categorization could be considered somewhat arbitrary, models were categorized as complex if they were generally less explainable, required increased computing power, or could more effectively manage irregularly sampled or high-dimensional data. Various publications have summarized these methods and offer insights about strengths and weaknesses^4–6^. All study designs were considered for inclusion. Articles were excluded if they did not report on original research or describe a structured review of the literature, did not focus on human populations, or were not published in the English language. Studies reporting on public opinion, vaccine uptake or adverse events, molecular docking, genomic sequencing, or applications in robotics were also excluded. Detailed inclusion and exclusion criteria are provided in Supplementary Table 1.

The Google search focused on grey literature describing the application of proprietary AI solutions by governments or industry for COVID-19 response, and other emerging applications not yet captured by the peer-reviewed and preprint literature. The same exclusion criteria were applied.

Box 1 Glossary of artificial intelligence-related terms Artificial intelligence^123^: computer applications that can perform tasks that normally require human intelligence.

Machine learning^123^: algorithms and models which machines can use to learn without explicit instructions.

Deep learning^123^: a subset of machine learning that generally uses neural networks.

Glossary Table 1 Distinction between complex and traditional models used for the scoping review^a^

**Table:** 

Complex machine learning techniques^b^ (in-depth review)	Traditional artificial intelligence and disease transmission models (limited review)
Adaptive boosting, decision trees, fuzzy logic, gradient boosting, k-means clustering, natural language processing, nearest neighbors, neural networks, random forests, support vector machines	Compartmental (e.g., Susceptible-Infected-Recovered), simulation (e.g., agent-based, Markov), statistical (e.g., Bayesian, exponential or logistic growth, linear or logistic regression), time series (e.g., auto regressive integrated moving average)

^a^Categorization of models as complex or traditional was somewhat arbitrary. Models were categorized as complex if they were generally less explainable, required increased computing power, or could more effectively manage irregularly sampled or high-dimensional data. This distinction was necessary given the large body of literature identified through the scoping review.

^b^Various publications have summarized these methods and offer insights about strengths and weaknesses^4–6^.

### Screening and data abstraction

Articles were screened in two stages using Covidence (Australia), a web-based review management tool. Articles were first screened for relevance based on the information provided in the title and abstract and then evaluated for inclusion based on the full text. Articles were screened by one reviewer at each stage. For articles that described the use of ML, the following criteria were abstracted into standardized forms: citation information; relevant use cases; respiratory pandemic (or SARS); population under study (i.e., region); purpose of the models (e.g., surveillance or prediction); type of ML models; outcomes of interest (e.g., infections or deaths); and data sources. Given the volume of relevant peer-reviewed and preprint literature reporting on traditional modeling approaches, data abstraction was not completed for studies included in the limited review. Manuscript details are provided in Supplementary Tables 2 and 3. Similarly, data were not abstracted for relevant grey literature.

### In-depth review of studies that applied machine learning techniques

The characteristics of studies that reported on the use of ML were summarized. Examples from the peer-reviewed, preprint, and grey literature were categorized into a framework of use cases related to public health and clinical practice. Each use case was narratively synthesized. Commonly used data sources and ML techniques were summarized in tabular form. Emerging use cases were identified as opportunities for future work.

### Limited review of studies that used traditional modeling approaches

The number of peer-reviewed articles and preprints that described traditional modeling approaches was reported to highlight the large volume of literature compared with manuscripts describing the application of ML. The objectives of these models and data sources were summarized in tabular form to identify additional areas where ML could be leveraged to provide more accurate estimations or projections.

## Results

**Fig. 1:**
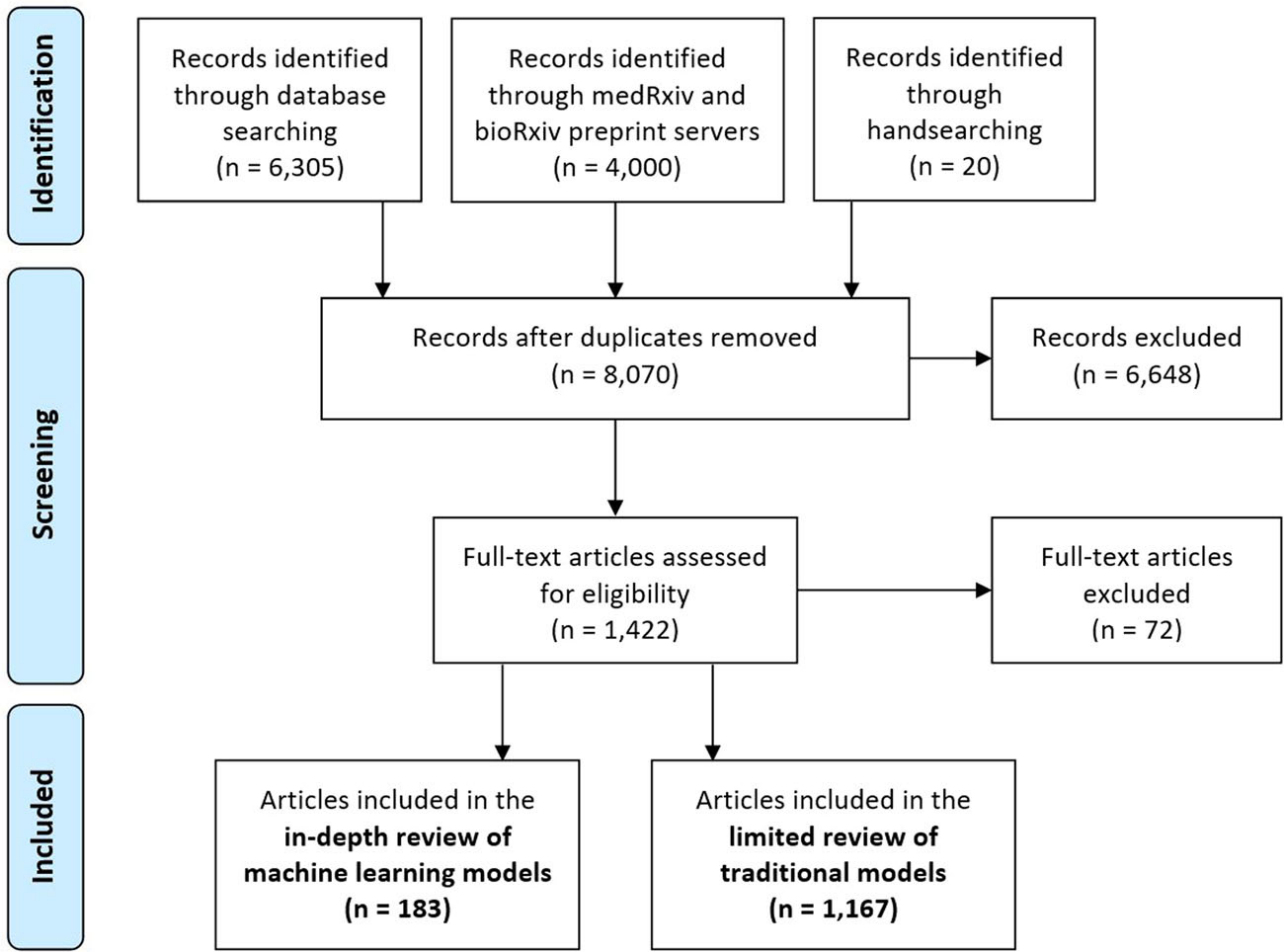
Study selection flow diagram. Modified Preferred Reporting Items for Systematic Reviews and Meta-Analyses (PRISMA) flow diagram showing disposition of articles.

From 8070 unique peer-reviewed and preprint records, 183 reported on the use of ML and met the inclusion criteria for the in-depth review (Supplementary Table 2). A modified Preferred Reporting Items for Systematic Reviews and Meta-Analyses (PRISMA) flow diagram is provided in Fig. 1. The review of the grey literature identified one additional use case not captured by the peer-reviewed or preprint literature and provided supporting examples for other use cases. Overall, the in-depth review identified six key use cases where ML was used for pandemic preparedness and response, as well as emerging areas beyond management of infectious disease, such as impacts of a pandemic on mental health or chronic conditions (Table 1).

**Table 1:** Number of manuscripts included in the in-depth review by use case and respiratory pandemic or SARS global outbreak^a^.

Use case	Hypothetical pandemic	SARS (2003)	H1N1 (2009)	COVID-19 peer-reviewed^b^	COVID-19 preprint^b^	Total
(1) Forecasting infectious disease dynamics and effects of interventions	1	2	4	4	29	40
(2) Surveillance and outbreak detection	1	1	11	–	3	16
(3) Real-time monitoring of adherence to public health recommendations	–	–	–	–	1	1
(4) Real-time detection of influenza-like illness	–	6	–	–	2	8
(5) Triage and timely diagnosis of infections	–	4	4	8	71	87
(6) Prognosis of illness and response to treatment	–	–	–	4	27	31
Structured reviews covering multiple use cases	–	–	–	2	1	3
Emerging areas beyond management of infection	–	–	–	2	4	6

*COVID-19* coronavirus disease 2019, *H1N1* pandemic influenza A subtype H1N1, *SARS* severe acute respiratory syndrome.

^a^A manuscript may cover multiple use cases.

^b^At the time of database searches (May 4, 2020).

The search also identified 1167 manuscripts that described the use of traditional modeling approaches and met the inclusion criteria for the limited review (Supplementary Table 3). A synthesis of the findings is presented in Box 2 and Table 2.

**Table 2:** Areas where machine learning could be leveraged for improving the accuracy of estimations or projections and potential data sources identified through the review of traditional approaches.

Use cases	Data sources
Infectious disease dynamics: R_0_, reproduction number^1^; peak time, intensity, and duration^2^; proportion of asymptomatic infections^3^; transmission rate^4^; household transmission^5^; spatiotemporal dynamics with GIS^6^; population immunity^7^	Published literature: seroconversion^40^; vaccine effectiveness^42^; estimates from other diseases (e.g., SARS)^46^
	Publicly available data: COVID-19 global cases from the Center for Systems Science and Engineering at Johns Hopkins University^47^; Worldometer COVID-19 pandemic updates^48^
Health outcomes: infections^8^; severe cases^9^; susceptibility^10^; deaths^11^; latency and infectious period^12^; undetected cases^13^; infections among healthcare workers^14^; infections among incarcerated populations^15^; infections among homeless populations^16^	
	Government data: government reported case data^1^; census data^49^; World Health Organization case reports and data on respiratory risk factors^50^; Ministry of Civil Aviation (i.e., airline passenger data)^6^; airport transportation data^51^; Armed Forces health surveillance data^52^; board of education school data (e.g., absentee reports that document H1N1)^53^; hospital/outpatient sentinel surveillance^21^
Impact on healthcare systems and demand for: inpatient beds^17^; intensive care unit beds^18^; N95 respirators and surgical masks^19^; ventilators^18^; medical supplies^20^; staffing^21^	
Effects of NPIs: social distancing^22^; self-isolation/quarantining^2^; workplace closures^23^; contact tracing^24^; wearing masks by general population^25^; handwashing^26^; optimal assay test pooling strategies for efficient testing^27^; frequency of routine testing for COVID-19 in high-risk environments to reduce workplace outbreaks^28^; mass testing/using drones to deliver tests^29^; periodic testing of health workforce^30^; impact of NPIs in residential care facilities^31^; closing borders^32^; effectiveness of airport thermal screening^33^; restrictions to sea, land and air travel^34^; school closures^35^; control measures for children if schools open (e.g., better ventilation, mask wearing)^36^; public risk communication^37^; media/news reports^37^; impact of available information on behavior and vaccination uptake^38^; ventilation of indoor spaces^39^	
	Mobile phone mobility data^54^
	Daily news reports^37^
	Purchasing information^55^
	Google mobility reports using five different categories: retail and recreation, grocery and pharmacy, transit stations, workplace, and residential^56^
	Google query and news data^57^
	Google Flu Trends data^58^
	Apple Maps COVID-19 mobility trends^59^
	Publicly available data on infection rates from cruise companies^60,61^
Impact of lifting NPI-related restrictions^40^	
Effects of pharmaceutical interventions: use of antiviral medications^41^; vaccination strategies^42^; disease spread given vaccination availability constraints^43^; transmission risk at immunization clinics^44^	National Oceanic and Atmospheric Administration: temperature, humidity, wind speed^62,63^
	Home television watching (i.e., proxy for time spent at home)^64^
Humanitarian assistance such as food distribution planning^45^	

^1-64^Provided in supplementary references.

*COVID-19* coronavirus disease 2019, *GIS* geographic information system, *H1N1* pandemic influenza A subtype H1N1, *NPI* non-pharmaceutical intervention, *SARS* severe acute respiratory syndrome.

Box 2 Limited review of traditional modeling approaches Of the 1167 studies that described the use of traditional models, 324 were peer-reviewed including 43 focused on COVID-19, and an additional 843 were COVID-19 preprints. The studies primarily focused on the modeling of infectious disease dynamics and forecasting to inform public health response. Compartmental models (e.g., Susceptible-Infected-Recovered models) were the most common. Others included agent-based, exponential or logistic growth, and autoregressive integrated moving average models. Examples of use cases and data sources that were used to develop these models are provided in Table 2.

**Fig. 2:**
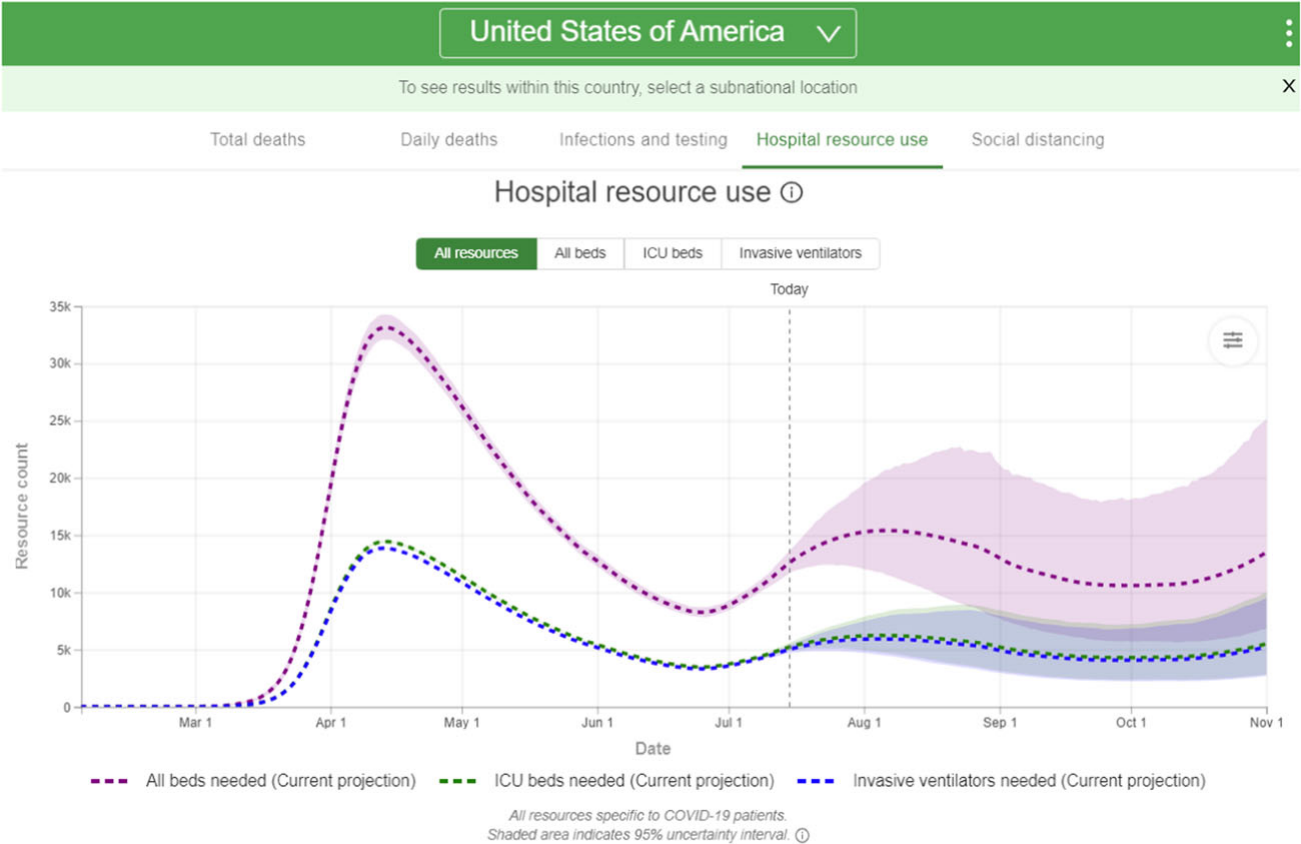
Example of projected hospital resource use for COVID-19 patients in the USA using traditional modeling approaches. The image shows forecasts for use of hospital beds, intensive care beds and ventilators over the next four months from the Institute for Health Metrics and Evaluation^124^. Image courtesy of the University of Washington, available under Public License and used with permission.

Traditional models were widely used to inform public health decision-making about implementation of non-pharmaceutical interventions to manage the COVID-19 pandemic. A notable example was the projections of infections, mortality, and hospital resource use available from the Institute for Health Metrics and Evaluation at the University of Washington under various countermeasures (Fig. 2)^124^. The models evolved over time and integrated additional data sources as the pandemic progressed to provide more accurate data-driven projections^125,126^.

The H1N1 peer-reviewed literature provided a few use cases that will be particularly important as effective preventive measures and therapeutics become available for COVID-19. Models could be used to forecast the impact of pharmaceutical interventions (e.g., antiviral medications), determine optimal vaccination strategies, and understand transmission risks of mass immunization clinics^127–129^. In the shorter-term, modeling could also help to compare various non-pharmaceutical interventions for children returning to school such as mask wearing or proper classroom ventilation, as well as determine the optimal frequency for routine workplace COVID-19 testing to reduce the impact of outbreaks^130,131^.

*COVID-19* coronavirus disease 2019, *H1N1* pandemic influenza A subtype H1N1.

### Forecasting infectious disease dynamics and effects of interventions

ML can be leveraged to improve the accuracy of estimations and projections to inform decision-making about the management of pandemics. Forty studies used ML to identify factors influencing spread of disease, fit epidemic curves, and forecast infectious disease dynamics or effects of interventions (40/183 studies [Supplementary Table 2]; 22%).

Most COVID-19 estimations and forecasts (32/33 studies; 97%) relied on relatively simplistic publicly available data sources such as counts from the Johns Hopkins COVID-19 map, Worldometer, and the World Health Organization as well as data released by country-specific Centers for Disease Control and Prevention, where ML may not provide much benefit compared with traditional modeling approaches. For example, one study used publicly available Worldometer and Google Trends data to project COVID-19 infections; however, traditional linear regression was shown to outperform a recurrent neural network-based model^7^.

Early in the pandemic, when data were limited, ML was used to augment traditional modeling approaches. Four studies used neural networks^8–11^ and one used a random forest algorithm^12^ to provide data-driven estimates of parameters for compartmental or statistical models. Two studies compared the performance of neural network-augmented models with traditional Susceptible-(Exposed)-Infected-Recovered models and showed that the augmented models provided better approximations of the true epidemic curve resulting in more accurate forecasts^8,10^.

Another approach involved augmenting sparse data. One study explored multiple approaches including random forests and variations of neural networks to forecast COVID-19 infections, deaths and effects of non-pharmaceutical interventions^13^. The models were trained using historical SARS data and fine-tuned using limited COVID-19 data. Similarly, a study published one month after the COVID-19 outbreak in Wuhan combined three strategies to develop an accurate ML model to forecast suspected infections by augmenting a 14-day COVID-19 dataset with other data sources, selecting the most appropriate model from a panel of models, and fine-tuning the parameters^14^. The final model used a polynomial neural network and showed significantly lower error compared with traditional time series modeling including autoregressive integrated moving average and exponential growth models.

As the pandemic progressed and more data became available, ML was leveraged to analyze temporal COVID-19 data and many studies integrated additional data sources such as health and demographic information, and geographic characteristics such as population density or climate. The most common techniques were variations of neural networks (Supplementary Table 2). These models forecasted various short- (e.g., 10 days^15^) or longer-term (e.g., 24 days^16^) outcomes including infections, deaths, and effects of non-pharmaceutical interventions; spread of COVID-19 across the globe^17^; and regional vulnerability to COVID-19^18^. Although many studies compared the relative performance of various ML techniques, these models were rarely evaluated against traditional approaches.

Similar ML-based approaches were also explored following SARS and H1N1 using historical data (Table 3). Most studies included in the limited review used traditional approaches to forecast infectious disease dynamics or effects of non-pharmaceutical interventions (Box 2). ML could be used to address other use cases presented in Table 2 with the advantage of integrating additional data sources and more effectively modeling irregularly sampled or high-dimensional data to provide more accurate predictions.

**Table 3:** Machine learning approaches explored in response to past or hypothetical pandemics and the SARS global outbreak by use case.

**Forecasting infectious disease dynamics and effects of interventions**
➢ In 2005, following the SARS global outbreak, two studies used neural networks to project the number of infections, deaths, hospital admissions, and/or effects of non-pharmaceutical interventions^90,91^.
➢ Between 2014 and 2018, three studies applied fuzzy logic to forecast effects of non-pharmaceutical interventions or optimal vaccination strategies using historical H1N1 data^92,93^, and allocation of hospital personnel during a hypothetical pandemic^94^.
➢ In 2018, various types of neural networks were used to estimate the reproductive number for a compartmental model using H1N1 data^95^.
➢ In 2012, a support vector machine was used to analyze Tweets posted during the H1N1 pandemic to inform forecasts of influenza-like illness^96^. These predictions demonstrated lower error than projections based on data from the Centers for Disease Control and Prevention alone.
**Surveillance and outbreak detection**
➢ In response to SARS, a 2004 conference proceeding described a prototype of a system to mine global news, weblogs, and other websites, then analyze and summarize the content to provide timely outbreak alerts^97^.
➢ Two studies applied various ML algorithms to mine emergency department electronic health record data for surveillance of influenza-like illness during the H1N1 pandemic using historical data^98,99^.
➢ Various studies used ML to mine and analyze Tweets from 2009–2010^96,100–104^. Support vector machines were used most often and outperformed other approaches. For example, support vector regression was able to estimate the prevalence of influenza-like illness 1–2 weeks earlier than standard reporting channels^103^.
➢ A similar approach was applied in 2015 to monitor the re-emergence of the H1N1 pandemic strain based on Tweets and web searches^105^. A follow-up study used rough sets theory to identify suspected cases^106^.
➢ A systematic review summarized the literature on the use of social media to track pandemics and highlighted that support vector machines were commonly used to classify Tweets for syndromic surveillance^107^.
➢ In 2016, an article described a smartphone application that used infrared thermal images for fever detection and interpreted audio using a k-nearest neighbors approach to identify coughing in public spaces^108^.
**Real-time monitoring of adherence to public health recommendations**
➢ No relevant studies were identified.
**Real-time detection of influenza-like illness**
➢ In 2005, neural network-based algorithms were developed in response to SARS to interpret thermal imaging for mass temperature screening^109–111^. The data were re-analyzed in 2010 using fuzzy neural networks^112,113^.
➢ In 2017, a study used radar to measure heart and respiratory rates coupled with thermal imaging interpreted using neural networks and k-means clustering to identify febrile air passengers with 98% sensitivity^114^.
**Triage and timely diagnosis of infections**
➢ In 2005, a study applied various ML algorithms to distinguish between SARS and typical pneumonia using chest X-rays. The best performing model was able to appropriately classify 71% of SARS patients^115,116^.
➢ In 2011, a National Institutes of Health study used a support vector machine to differentiate H1N1 pneumonia from healthy lungs as well as other infectious and chronic lung conditions based on CT images with AUCs >0.99; however, the study only included images from four patients diagnosed with H1N1^117^.
➢ In 2006, a hierarchical fuzzy signature was used to accurately distinguish SARS patients from healthy individuals as well as those with pneumonia or hypertension based on vital signs and clinical symptoms^118^.
➢ In 2014, two studies applied case-based reasoning using a neural network and/or nearest neighbors approach to identify patients with H1N1 based on symptoms with accuracies of 95% and 86%^119,120^.
➢ In 2014, a study used random forests and boosted regression trees to identify risk factors associated with contracting the H1N1 pandemic strain in the following 2010–2011 influenza season^121^.
➢ In 2019, a recurrent neural network and decision tree-based model was proposed for detection of SARS. The system was able to identify cases using user-generated text and geospatial information with 91% accuracy^122^.
**Prognosis of illness and response to treatment**
➢ No relevant studies were identified.

*AUC* area under the curve, *COVID-19* coronavirus disease 2019, *CT* computed tomography, *H1N1* pandemic influenza A subtype H1N1; *SARS* severe acute respiratory syndrome.

### Surveillance and outbreak detection

The grey literature highlighted many examples where ML was used for outbreak detection. For example, industry-based companies and Boston Children’s Hospital HealthMap (USA) were among the first outside of China to report the emerging risk of COVID-19 by leveraging natural language processing (NLP) to translate and analyze foreign news reports^19^.

Sixteen studies reported on the use of ML for surveillance or outbreak detection (16/183 studies [Supplementary Table 2]; 9%). However, only three studies focused on COVID-19. Two preprints used NLP and deep neural networks to mine and analyze Twitter posts for personal reports of potential exposure to COVID-19^20,21^. Another preprint described leveraging data from smartphone-connected thermometers to monitor rates of influenza-like illness and flag higher than expected rates^22^. Both approaches tracked potential exposures or symptoms in real time coupled with precise geolocation information to understand where outbreaks were occurring. These data sources could also be used to forecast influenza-like illness rates.

Similar approaches were explored in response to or following SARS and H1N1 (Table 3). In addition, clinical information, such as electronic health record (EHR) data from emergency departments, was shown to be an informative data source for monitoring rates of influenza-like illness using historical H1N1 data. A smartphone application (app) was also developed for syndromic surveillance in public spaces.

### Real-time monitoring of adherence to public health recommendations

The peer-reviewed and preprint literature did not provide examples of how ML was used in real time to improve adherence to public health recommendations; all the examples were found in the grey literature. This use case was not explored in response to past pandemics or the SARS global outbreak.

Early in the COVID-19 pandemic, some countries such as China and Russia leveraged existing AI-based facial-recognition software and cameras to identify individuals who were not compliant with mandated self-isolation or quarantine^23,24^. This technology also advanced to accurately identify those wearing a mask for mass public monitoring^24^. On a smaller scale, contactless verification of employees was proposed for returning to work^25^.

**Fig. 3:**
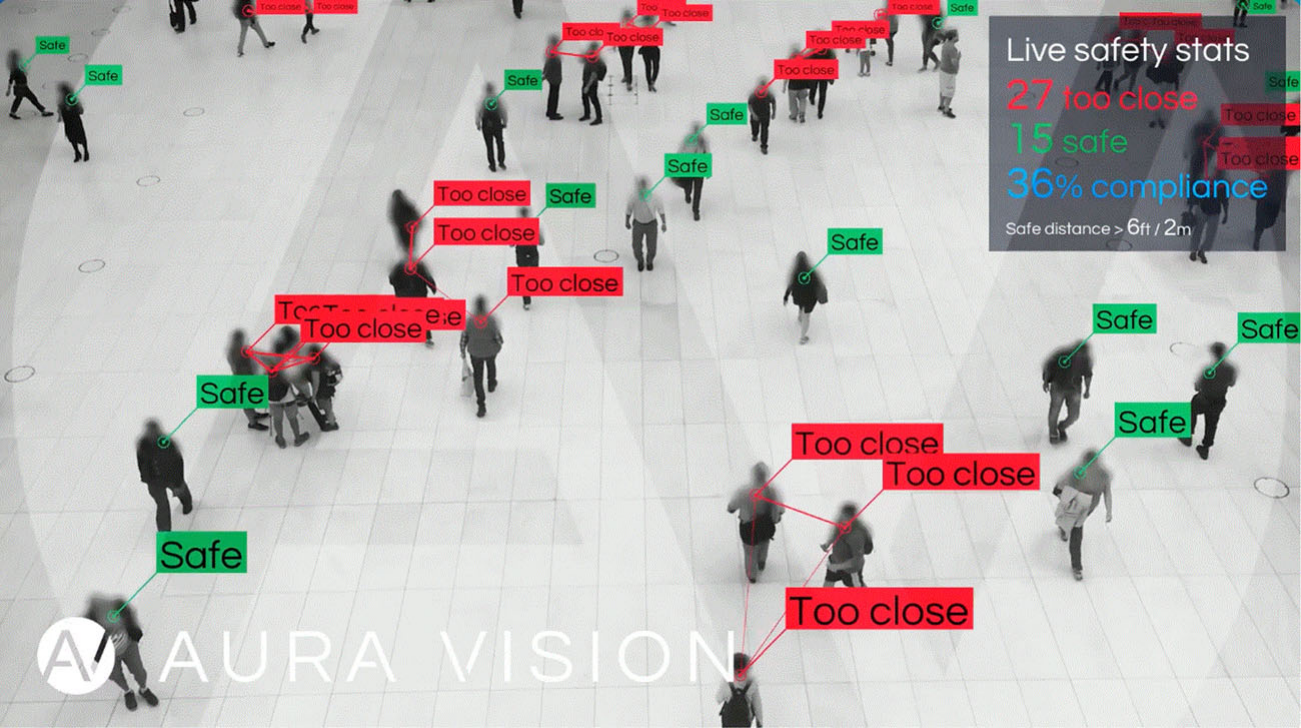
Example of a computer vision solution for real-time monitoring of adherence to social distancing^26^. The image shows the movement of people through a public space and estimates compliance with distancing by at least 6 feet. Image courtesy of Aura Vision, used with permission.

To address privacy concerns, alternatives based on facial detection rather than recognition were developed to help businesses, schools, and workplaces reopen safely. Numerous companies developed computer vision-based solutions to monitor and improve adherence to public health recommendations such as wearing masks, social distancing, and hand sanitization by analyzing closed-circuit surveillance videos using neural networks (Fig. 3)^26,27^. Clients were able to receive daily summaries or real-time alerts to help improve adherence to protect employees and visitors. Additional features included tracking store capacity and prioritizing areas for timely sanitation^26^.

Similar computer vision systems were developed for hospitals to monitor interactions with COVID-19 patients at the bedside and document which employees entered the room and for how long, whether there was close contact with the patient, and if PPE was secure^28^. As a next step, industry was developing computer vision applications to monitor healthcare PPE inventory in real time^29^.

The review identified one related preprint where ML was used to help decision-makers understand adherence to non-pharmaceutical interventions in near real time (1/183 studies [Supplementary Table 2]; <1%). Deep neural networks were used for travel mode detection to calculate various population-level mobility and social distancing metrics reported daily on the COVID-19 Impact Analysis Platform^30^.

### Real-time detection of influenza-like illness

Computer vision solutions were also developed to detect influenza-like illness consistent with viral respiratory pandemic symptoms for mass screening (8/183 studies [Supplementary Table 2]; 4%). However, only two studies focused on COVID-19.

The first COVID-19 study employed computer vision to assess for both fever and cyanosis with 97% and 77% accuracies, respectively^31^. Similar approaches were developed following SARS, however, the types and quality of sensor data and ML techniques improved over time (Table 3). The grey literature showed that thermal scanners were widely deployed for COVID-19 in hospitals and public spaces^29^, although underlying data sources, models and performance may have varied. ‘Pandemic’ drones were also developed to detect influenza-like illness remotely including fever, increased heart and respiratory rates, as well as more overt symptoms such as coughing^32^.

The other COVID-19 study developed a smartphone app that differentiated COVID-19 coughs from other types using convolutional neural networks (CNN) and a support vector machine, and demonstrated promising accuracy^33^. The grey literature search identified another app that was under development and aimed to detect COVID-19 by analyzing voice recordings^34^.

Data from wearable devices were also leveraged for early detection of COVID-19. A press release reported that algorithms integrating data collected by the Oura Ring (Oura Health Ltd, Finland) with patient-reported data from a COVID-19 monitoring app were able to detect subclinical signs of infection up to 3 days prior to onset of classic symptoms such as fever or cough with 90% accuracy^35^.

### Triage and timely diagnosis of infections

The most common use case for the application of ML for pandemic response was triage and timely diagnosis of symptomatic cases (87/183 studies [Supplementary Table 2]; 48%). Most studies developed algorithms or tools in response to COVID-19 (78/87; 90%), and one study conducted a systematic review of these tools^36^. Eight studies (9%) reported on the development of similar tools following H1N1 or SARS (Table 3).

The use of ML for detection or estimation of disease severity based *solely* on chest imaging made up the bulk of COVID-19 original research (65/78 studies [Supplementary Table 2]; 83%). Most studies relied on open-source datasets and leveraged some variation of CNNs for image segmentation, classification to differentiate between COVID-19 and other common lung infections, or estimation of disease severity (Table 4). The algorithms showed varying performance with AUCs ranging from 0.81 to >0.99, which could have been impacted by size or quality of the data source, type of imaging, approaches to image processing, types of ML used, and fine-tuning of parameters.

**Table 4:** Characteristics of studies that developed machine learning-based algorithms and tools for COVID-19 diagnosis or estimation of disease severity based *solely* on chest imaging (*n* = 65).

Characteristic	*n*	%
Purpose^a^		
Image segmentation only	2	3
Detection of COVID-19	56	86
Estimation of COVID-19 severity	9	14
Type of chest imaging^a^		
CT	35	54
X-ray	32	49
Data sources		
Private medical imaging data	23	35
Publicly available data	42	65
Machine learning models^a,b^		
Convolutional neural network	58	89
Support vector machine	9	14
Other^c^	11	17
Proprietary	2	3
Publication status^d^		
Peer-reviewed	7	11
Preprint	58	89

*COVID-19* coronavirus disease 2019, *CT* computed tomography.

^a^Totals add to more than 65 and percentages add to more than 100; some studies cover multiple categories.

^b^Six studies also covered the *Prognosis of Illness and Response to Treatment* use case; in these cases, some of the models may have been applied for prediction of disease severity, response to treatment, or death.

^c^Adaptive boosting (3); autoencoder (2); decision tree (6); explainable deep neural network (1); fuzzy neural network (1); gradient boosted trees (2); k-nearest neighbors (3); multi-layer perceptron (4); random forest (5); unspecified neural network (1).

^d^At the time of database searches (May 4, 2020).

ML algorithms incorporating other information beyond imaging were also developed to help prioritize patients with a higher likelihood of COVID-19 for isolation and testing. Nine studies developed models using combinations of standard variables such as patient demographics, vital signs, clinical symptoms, comorbidities, and known exposure history, as well as CT images^37–45^; most also included the results from routine bloodwork (8/9 studies; 89%). The algorithms were developed using a wide array of ML approaches and showed varying performance with AUCs ranging from 0.84 to >0.99.

Similarly, one preprint used results from routine bloodwork to estimate COVID-19 disease severity^46^. Another study used a transformer neural network to identify symptoms documented in unstructured clinical notes from an EHR during the week leading up to COVID-19 testing, which could be used to inform development of triage tools^47^.

**Fig. 4:**
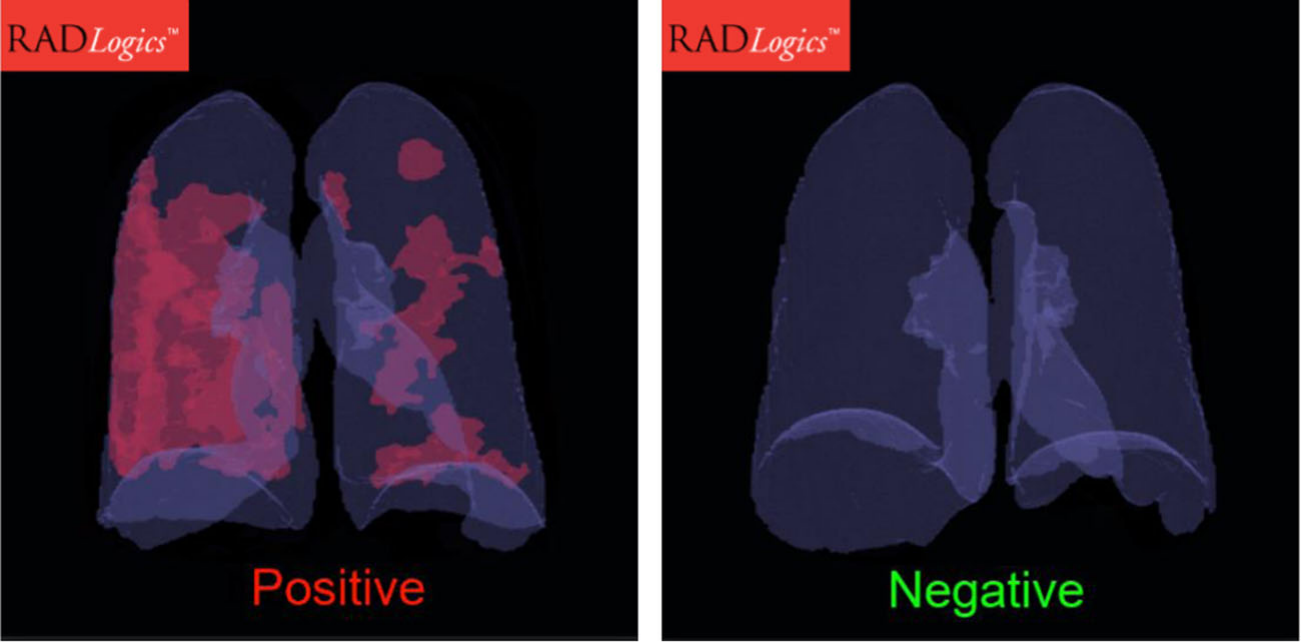
Example of a ML solution for detecting and estimating the extent of COVID-19 infection based on CT images. The image on the left shows a patient infected with COVID-19. The image on the right shows a patient negative for COVID-19. Images courtesy of RADLogics, Inc., used with permission.

Although studied in research settings, these types of clinical decision support tools were not widely available to assist clinicians with timely diagnosis of COVID-19. There were a few notable exceptions. One article described the rapid development and deployment of an ML-based COVID-19 diagnostic system that screened CT images across 16 hospitals in China^48^. More strategically, some healthcare software companies, such as RADLogics, Inc., have adapted existing solutions to accurately detect COVID-19 from CT images and quantify the extent of infection in a clinically interpretable way (Fig. 4)^49,50^.

In contrast, the grey literature reported widespread development and implementation of AI-based chatbots for large-scale public triage by governments and healthcare organizations during the COVID-19 pandemic^51^. These tools were not described in the peer-reviewed or preprint literature, and as a result, the underlying models and appropriateness of chatbot recommendations were generally not known. Only one preprint reported on the *Symptoma* chatbot (Austria), which was shown to have 96% accuracy for detecting COVID-19^52^. Another study used gradient boosting to develop a model for COVID-19 triage for testing using data collected from national symptom surveys and demonstrated an AUC of 0.73^53^.

### Prognosis of illness and response to treatment

ML models were also commonly developed to predict which patients were at higher risk of COVID-19-related deterioration (31/183 studies [Supplementary Table 2]; 17%), including one systematic review^36^. This use case was not explored in response to past pandemics.

Original research focused on predicting progression to severe disease, intensive care admission, ventilator use, or mortality (Table 5). Almost half of the studies used data routinely captured by an EHR or obtained through a quick patient history, and explored various classification algorithms (13/30 studies; 43%)^54–66^. Nine studies compared the performance of complex ML with simple logistic regression^54–62^. In four studies, logistic regression was found to have similar or better performance^55,57,58,60^, and of three studies that developed a clinical prediction tool, two selected logistic regression as the final model for simplicity and interpretability^55,60^. Similarly, another study developed a model using extreme gradient boosted decision trees, but deferred to an explainable single tree for the final model^66^.

**Table 5:** Characteristics of studies that developed machine learning-based algorithms and tools to predict COVID-19-related deterioration (*n* = 30).

Characteristic	*n*	%
Prediction^a^		
Severity of COVID-19	8	27
Hospitalization	1	3
Length of hospital stay	2	7
Intensive care unit admission	7	23
Ventilator use	7	23
Discharge to hospice	1	3
Death	18	60
Response to treatment	1	3
Patients at higher risk of severe complications if infected	1	3
Type of chest imaging		
CT	8	27
X-ray	6	20
None	16	53
Data sources^a^		
Health records		
Electronic	8	27
Unclear if electronic	12	40
Publicly available data	13	43
Machine learning models^a,b^		
Random forest	14	47
Convolutional neural network	12	40
Gradient boosted trees	9	30
Support vector machine	8	27
Other^c^	9	30
Proprietary	2	7
Publication status^d^		
Peer-reviewed	3	10
Preprint	27	90

*COVID-19* coronavirus disease 2019, *CT* computed tomography.

^a^Totals add to more than 30 and percentages add to more than 100; some studies cover multiple categories.

^b^Six studies also covered the *Triage and Timely Diagnosis of Infections* use case; in these cases, some of the models may have been applied for detection of COVID-19 or estimation of disease severity.

^c^Adaptive boosting (1); decision tree (5); k-nearest neighbors (4); multi-layer perceptron (2); natural language processing (1); recurrent neural network (2); unspecified neural network (3).

^d^At the time of database searches (May 4, 2020).

On the other hand, ML can help to make sense of large amounts of complex, or unstructured data. Thirteen studies used CT or X-ray images to predict deterioration alone or in combination with clinical information (Supplementary Table 2) with AUCs ranging from 0.70 to 0.97. Another study predicted which hospitalized patients would be admitted to intensive care by analyzing unstructured EHR notes using the proprietary NLP- and neural network-based EHRead (Savana, Madrid) extraction technology^67^. The data were then used to classify patients using a decision tree yielding an AUC of 0.76.

Existing tools, such as the Epic Deterioration Index (Epic Systems Corporation, USA), were used to predict COVID-19-related deterioration; the index showed moderate performance with an AUC of 0.79^68^. Other prognostic systems were studied in research settings, but these tools were not widely available to assist clinicians early in the pandemic but slowly came to market. For example, CLEWICU (CLEW Medical Ltd., Israel) received Emergency Use Authorization from the U.S. Food and Drug Administration (FDA) in June 2020 for use in hospitals to predict respiratory failure and hemodynamic instability in COVID-19 patients^69^.

Although there were no known specific treatments for COVID-19 early on, one preprint described an algorithm for prediction of patients’ response to treatment based on age, chronic conditions, respiratory or organ failure, and treatment plan to guide the use of limited healthcare resources^70^. The best performing model had an AUC > 0.99 and was developed using a CNN for image interpretation coupled with a support vector machine to integrate clinical data for prediction of response to treatment.

### Emerging areas beyond management of infection

The COVID-19 pandemic affected health broadly beyond the outcomes of the infectious disease itself. Literature describing the use of ML in other domains, such as mental health and chronic conditions, was emerging even early in the pandemic (6/183 studies [Supplementary Table 2]; 3%). One study reported on the short-term mental health impacts of COVID-19 through sentiment analysis of social media posts before and shortly after the initial outbreak in Wuhan^71^. Another study used ML to group related literature on the impact of coronaviruses on people with intellectual disabilities^72^.

In response to the large body of COVID-19-related literature, the *COVID-19 Open Research Dataset* was released with a ‘call to action’ for academic and industry researchers to develop AI techniques to rapidly analyze the literature to address important knowledge gaps^73^. Three responses to this call were identified: two studies identified themes in the literature using k-means clustering or lexical link analysis, and another study used NLP to generate summaries of the relevant literature^72,74,75^. Two other studies described the use of NLP and/or neural networks to mine other sources of publicly available literature and summarize the results^76,77^.

To ensure that impactful, high-quality research could be used to guide pandemic response, ML techniques were used to identify promising research uploaded to preprint servers for expedited peer-review^78^. Reviews were published in the open-access journal *Rapid Reviews: COVID-19*. These approaches aimed to quickly create evidence bases that could be used to inform public health and clinical decision-making. In addition, neural networks were also applied to limit the spread of misinformation about the COVID-19 pandemic^79^.

## Discussion

We performed a scoping review of the peer-reviewed, preprint and grey literature, and identified six key use cases where ML was leveraged for pandemic preparedness or response. We also found that the sources of data and types of ML that were useful varied by use case (Table 6). While there were many examples of novel solutions, most were still at the research or developmental stage and had not been widely used to inform clinical or public health decisions early in the COVID-19 pandemic. For example, despite numerous publications demonstrating good to excellent performance in diagnosing COVID-19 from lung imaging, practical prospective clinical applications of these algorithms were rare; possibly, due to many algorithms being developed based on the availability of data and knowledge of ML, rather than to address specific clinical or public health-driven questions. However, some existing products were adapted or modified for implementation; these included computer vision for real-time monitoring of adherence to public health recommendations or detection of influenza-like illness, as well as specific tools for triage such as chatbots. A few ML solutions received FDA Emergency Use Authorization for use in clinical settings to detect COVID-19 or predict infection-related deterioration^69,80^. Most examples of tools that were rapidly implemented were identified through the grey literature and were developed by health systems or industry.

**Table 6:** Commonly used data sources and types of machine learning suitable for each use case based on studies from the in-depth review about past pandemics, SARS, and COVID-19.

Key use case	Commonly used data sources	Suitable types of ML
Forecasting infectious disease dynamics and effects of interventions	Publicly available counts (e.g., Johns Hopkins COVID-19 map, Worldometer, World Health Organization), media reports, commercial publications, web searches (e.g., Google Trends), social media (e.g., Twitter), census data, population-level comorbidity statistics, data on outbreaks of similar pathogens	Augmenting traditional models: neural networks
		Data-driven ML: recurrent neural networks
Surveillance and outbreak detection	Social media (e.g., Twitter), web searches, news reports, medical record data (structured and unstructured fields)	Text mining: natural language processing
		Classification: support vector machines, transformer neural networks
Real-time monitoring of adherence to public health recommendations	Cameras in public spaces	Compliance with mandated quarantine: proprietary facial recognition
		Adherence to mask wearing, social distancing, and sanitation: proprietary computer vision
Real-time detection of influenza-like illness	Cameras and sensors in public spaces	Interpretation of thermal imaging: neural networks, computer vision
Triage and timely diagnosis of infections	Exposure history, medical record data (structured and unstructured fields, laboratory results, chest imaging)	Interpretation of chest imaging: convolutional neural networks
		Triage based on routinely collected medical record data: no standout ML
		Interpretation of unstructured clinical notes: transformer neural networks
Prognosis of illness and response to treatment	Medical record data (structured and unstructured fields, laboratory results, chest imaging)	Based on chest imaging: combination of convolutional and recurrent neural networks
		Based on routinely collected medical record data: no standout ML
		Interpretation of unstructured clinical notes: natural language processing, neural networks

*ML* machine learning, *SARS* severe acute respiratory syndrome.

Given the relative technological limitations during past pandemics and SARS, understandably most research relied on traditional modeling approaches. However, the limited review included in our study highlighted that there was still a strong reliance on traditional approaches in response to COVID-19 and identified additional areas where ML could be leveraged to improve performance (Table 2). ML is well positioned to complement traditional modeling in the following ways: (1) Integration of diverse sources of information: ML methods are better at integrating diverse and complex sources of data than traditional statistical regression models; (2) Combination of different types of models: ensemble learning or data augmentation methods can be used to combine different types of prediction models to achieve better accuracy^81^, or more granular models; (3) Temporal modeling: while traditional time series modeling or statistical methods can be effective for dealing with regularly sampled and low-dimensional temporal data, data from a pandemic tend to be irregularly sampled and high dimensional, where ML methods such as neural networks could substantially improve performance.

In addition to the use cases described in this article, ML approaches also played a key role in other aspects of pandemic response. One area was genome sequencing, where ML was used for classification of COVID-19 viral genomes, which allowed for rapid detection of unknown mutations and supported contact tracing by determining the genetic origin of each case^82,83^. On a molecular level, ML was used to understand the underlying structures of associated proteins and molecular docking processes^84^. This knowledge could inform vaccine development or identification of effective drug treatments.

### Challenges of employing machine learning

The performance of ML algorithms depends on the availability and accessibility of vast amounts of data, conditions that are subject to technology infrastructure and interoperability, and privacy and data-sharing laws. In many cases, even the most basic infrastructure necessary to transmit data between healthcare organizations was lacking. For example, based on 2018 data, 41% of US hospitals were not able to electronically report surveillance data to public health agencies^85^.

Moreover, when datasets do exist, a lack of comprehensive and diverse data is a critical challenge. In instances where training data systematically exclude parts of the population (e.g., asymptomatic cases due to lack of testing; or individuals who do not have access to data collection using consumer-centric technologies, such as wearables or smartphones, or reliable Internet service), the applicability of the model to wider populations could be compromised. Data quality could be further compromised by incomplete or inconsistent labeling of racial, ethnic, and other demographic information^86^. Based on biased or limited samples, ML algorithms may inadvertently increase disparities by misrepresenting the burden of disease and inappropriately informing resource allocation.

ML algorithms and tools also face challenges at deployment. Health systems and public health experts must exercise caution when applying models in different contexts. Algorithms trained in a specific health system, cultural or socio-economic context may not provide similar performance for populations with different characteristics. Algorithms must undergo critical evaluation and re-calibration when implemented across settings which requires time, as well as financial and human resources.

Interpretability of ML solutions can also limit approval, implementation, or adoption of these tools in real-world settings. There is generally a tradeoff between model complexity and interpretability that needs to be considered particularly in healthcare settings, given ethical and legal implications of decision-making. A few studies identified through the review compared ML with traditional approaches and in some cases simpler models demonstrated similar or better performance while offering the additional benefit of interpretability, highlighting the importance of comparing ML-based algorithms and solutions with traditional approaches to ensure that increased complexity adds value. However, for many use cases highly complex models were necessary for tasks like image interpretation, and many of the tools described in this review offered some level of interpretability by highlighting physical locations where people were not social distancing (Fig. 3) or areas on chest imaging contributing to detection of COVID-19 (Fig. 4).

### Overarching lessons

These findings have several overarching lessons. First, past pandemics and the SARS global outbreak were followed by spurts of research, followed by rapid declines in research support for approaches that could have enabled better management of COVID-19^87,88^. As such, longitudinal support is essential for this work. Second, while ML was explored widely early in the COVID-19 pandemic as evidenced by the preprint search, almost all this work was at the research or developmental stage, and real-world applications were limited. Third, support for development of large and comprehensive databases preferably at the national, and even international level, containing health data would be extremely valuable for many purposes, with pandemic management being among the most important^89^. Fourth, tools should be developed that allow modeling of multiple scenarios to make better choices about the wide array of options that need to be considered, from choices about school closures to care management for the elderly, to distribution of scarce resources like ventilators and PPE.

### Limitations of the study

This study has several limitations. Each record was evaluated by one reviewer due to the large number of studies identified through the search. As a scoping review, the goal was to provide an overview of key use cases for ML rather than a comprehensive evaluation of specific data sources or ML approaches. Future work is warranted to assess the risk of bias and usability of these solutions in practical settings.

The review included preprint articles to capture the breadth of the rapidly growing body of literature about the COVID-19 pandemic. However, the preprint articles were not peer-reviewed and results should be interpreted with caution.

The grey literature search included reputable trade and commercial publications to identify applications of proprietary AI solutions by governments or industry for COVID-19 response, and other emerging applications not yet captured by the peer-reviewed and preprint literature. Although not the standard approach, it was considered appropriate given the context, where trade and commercial publications have been a valuable source of information throughout the COVID-19 pandemic.

## Conclusions

Important ML-based solutions have been developed in response to pandemics and particularly for COVID-19 but few were optimized for practical clinical or public health application early in the pandemic. These findings can support policymakers, clinicians, and other stakeholders in prioritizing operationalization of AI for future pandemics.

## Supplementary information

Supplementary Information

## Data Availability

All data generated or analyzed during this study are included in this published article and its supplementary information.
