# Supplementary material for: Leveraging artificial intelligence for pandemic preparedness and response: a scoping review to identify key use cases
Source: NPJ Digit Med. 2021 Jun 10;4:96. doi: 10.1038/s41746-021-00459-8 (PMC8192906; doi:10.1038/s41746-021-00459-8)
Supplement: Supplementary file 1 — Supplementary Information [file 41746_2021_459_MOESM1_ESM.pdf]

<u>Supplementary Note 1</u>. Database search strategies

PubMed (NCBI) – 2020/05/04 – 1,534 records

("Pandemics"[Mesh] OR "SARS Virus"[Mesh] AND "Coronavirus Infections"[Mesh:noexp] OR "Severe Acute Respiratory Syndrome"[Mesh] OR "Influenza A Virus, H1N1 Subtype"[Mesh] OR pandemic*[tiab] OR coronavirus*[tiab] OR 2019-nCoV[tw] OR 2019nCoV[tw] OR COVID-19[tw] OR covid19[tw] OR SARS-CoV-2[tw] OR SARS-CoV2[tw] OR SARSCoV2[tw] OR severe acute respiratory syndrome[tiab] OR sars[tiab] OR influenza[tiab] OR flu[tiab] OR h1n1[tiab])

AND ("Artificial Intelligence"[mesh] OR "Pattern Recognition, Automated"[mesh] OR artificial intelligence*[tiab] OR ai model*[tiab] OR computational intelligence*[tiab] OR machine intelligence* OR intelligent system*[tiab] OR computer reasoning[tiab] OR machine learning[tiab] OR deep learning[tiab] OR supervised learning[tiab] OR expert system*[tiab] OR fuzzy logic[tiab] OR language processing[tiab] OR language processor*[tiab] OR neural network*[tiab] OR perceptron*[tiab] OR connectionist*[tiab] OR pattern recognition[tiab] OR random forest[tiab] OR computer vision[tiab] OR multiple kernel[tiab] OR multi kernel[tiab] OR multikernel[tiab] OR knn[tiab] OR rnn[tiab] OR nearest neighb*[tiab] OR kmeans[tiab] OR k means[tiab] OR ensemble learning[tiab] OR ensemble model*[tiab] OR seir[tiab] OR sir[tiab] OR susceptible exposed infectious recovered[tiab] OR susceptible infectious recovered[tiab] OR kalman filter*[tiab] OR data simulation*[tiab] OR structured kernel[tiab] OR gaussian process*[tiab] OR transfer learning[tiab] OR state space[tiab] OR bayesian[tiab] OR non-parametric method*[tiab] OR nonparametic method*[tiab] OR graph convolutional network*[tiab] OR causal inference*[tiab] OR counterfactual estimation*[tiab] OR counterfactual simulation*[tiab] OR adversarial network*[tiab] OR trial enrichment[tiab] OR trial simulation*[tiab])

Embase (Elsevier; 1974 -) – 2020/05/04 – 1,730 records

('pandemic'/exp OR 'sars coronavirus'/exp OR 'covid 19'/exp OR 'Coronavirus infection'/de OR 'severe acute respiratory syndrome'/exp OR 'Influenza A virus (H1N1)'/exp OR pandemic*:ab,ti OR coronavirus*:ab,ti OR '2019-nCoV':ab,ti OR 2019nCoV:ab,ti OR 'COVID-19':ab,ti OR covid19:ab,ti OR 'SARS-CoV-2':ab,ti OR 'SARS-CoV2':ab,ti OR sarscov2:ab,ti OR 'severe acute respiratory syndrome':ab,ti OR sars:ab,ti OR influenza:ab,ti OR flu:ab,ti OR h1n1:ab,ti)

AND ('artificial intelligence'/exp OR 'machine learning'/exp OR 'artificial intelligence*':ab,ti OR 'ai model*':ab,ti OR 'computational intelligence*':ab,ti OR 'machine intelligence*' OR 'intelligent system*':ab,ti OR 'computer reasoning':ab,ti OR 'machine learning':ab,ti OR 'deep learning':ab,ti OR 'supervised learning':ab,ti OR 'expert system*':ab,ti OR 'fuzzy logic':ab,ti OR 'language processing':ab,ti OR 'language processor*':ab,ti OR 'neural network*':ab,ti OR 'perceptron*':ab,ti OR 'connectionist*':ab,ti OR 'pattern recognition':ab,ti OR 'random forest':ab,ti OR 'computer vision':ab,ti OR 'multiple kernel':ab,ti OR 'multi kernel':ab,ti OR 'multikernel':ab,ti OR 'knn':ab,ti OR 'rnn':ab,ti OR 'nearest neighb*':ab,ti OR 'kmeans':ab,ti OR 'k means':ab,ti OR 'ensemble learning':ab,ti OR 'ensemble model*':ab,ti OR 'seir':ab,ti OR 'sir':ab,ti OR 'susceptible exposed infectious recovered':ab,ti OR 'susceptible infectious recovered':ab,ti OR 'kalman filter*':ab,ti OR 'data simulation*':ab,ti OR 'structured kernel':ab,ti OR 'gaussian process*':ab,ti OR 'transfer learning':ab,ti OR 'state space':ab,ti OR

'bayesian':ab,ti OR 'non-parametric method*':ab,ti OR 'nonparametic method*':ab,ti OR 'graph convolutional network*':ab,ti OR 'causal inference*':ab,ti OR 'counterfactual estimation*':ab,ti OR 'counterfactual simulation*':ab,ti OR 'adversarial network*':ab,ti OR 'trial enrichment':ab,ti OR 'trial simulation*':ab,ti)

NOT ('conference abstract'/it OR 'conference paper'/it OR 'conference review'/it)

Web of Science - Indexes=SCI-EXPANDED, SSCI, A&HCI, CPCI-S, CPCI-SSH, BKCI-S, BKCI-SSH, ESCI, CCR-EXPANDED, IC Timespan=All years – 2020/05/04 – 2,296 records

TS=("pandemic*" OR "coronavirus*" OR "2019-nCoV" OR "2019nCoV" OR "COVID-19" OR "covid19" OR "SARS-CoV-2" OR "SARS-CoV2" OR "SARSCoV2" OR "severe acute respiratory syndrome" OR "sars" OR "influenza" OR "flu" OR "h1n1")

AND TS=("artificial intelligence*" OR "ai model*" OR "computational intelligence*" OR "machine intelligence*" OR "intelligent system*" OR "computer reasoning" OR "machine learning" OR "deep learning" OR "supervised learning" OR "expert system*" OR "fuzzy logic" OR "language processing" OR "language processor*" OR "neural network*" OR "perceptron*" OR "connectionist*" OR "pattern recognition" OR "random forest" OR "computer vision" OR "multiple kernel" OR "multi kernel" OR "multikernel" OR "knn" OR "rnn" OR "nearest neighb*" OR "kmeans" OR "k means" OR "ensemble learning" OR "ensemble model*" OR "seir" OR "sir" OR "susceptible exposed infectious recovered" OR "susceptible infectious recovered" OR "kalman filter*" OR "data simulation*" OR "structured kernel" OR "gaussian process*" OR "transfer learning" OR "state space" OR "bayesian" OR "non-parametric method*" OR "nonparametic method*" OR "graph convolutional network*" OR "causal inference*" OR "counterfactual estimation*" OR "counterfactual simulation*" OR "adversarial network*" OR "trial enrichment" OR "trial simulation*")

IEEE Xplore – 2020/05/04 – 353 records

("All Metadata:"pandemic*" OR "coronavirus" OR "2019-nCoV" OR "2019nCoV" OR "COVID-19" OR "covid19" OR "SARS-CoV-2" OR "SARS-CoV2" OR "SARSCoV2" OR "severe acute respiratory syndrome" OR "sars" OR "influenza" OR "flu" OR "h1n1")

AND ("All Metadata":"artificial intelligence" OR "ai model*" OR "computational intelligence" OR "machine intelligence" OR "intelligent system*" OR "computer reasoning" OR "machine learning" OR "deep learning" OR "supervised learning" OR "expert system" OR "expert systems" OR "fuzzy logic" OR "language processing" OR "language processor" OR "neural network*" OR "perceptron*" OR "connectionist" OR "pattern recognition" OR "random forest" OR "computer vision" OR "multiple kernel" OR "multi kernel" OR "multikernel" OR "knn" OR "rnn" OR "nearest neighbor" OR "nearest neighbour" OR "kmeans" OR "k means" OR "ensemble learning" OR "ensemble model" OR "seir" OR "sir" OR "susceptible exposed infectious recovered" OR "susceptible infectious recovered" OR "kalman filter" OR "data simulation" OR "structured kernel" OR "gaussian process" OR "transfer learning" OR "state space" OR "bayesian" OR "non-parametric method" OR "nonparametic method" OR "graph convolutional network" OR "causal inference" OR "counterfactual estimation" OR "counterfactual simulation" OR "adversarial network*" OR "trial enrichment" OR "trial simulation")

ACM Guide to Computing Literature – 2020/05/04 – 392 records

Publication Title (pandemic* OR coronavirus OR "2019-nCoV" OR 2019nCoV OR "COVID-19" OR covid19 OR "SARS-CoV-2" OR "SARS-CoV2" OR SARSCoV2 OR "severe acute respiratory syndrome" OR sars OR influenza OR flu OR h1n1)

OR Publication Abstract (pandemic* OR coronavirus OR "2019-nCoV" OR 2019nCoV OR "COVID-19" OR covid19 OR "SARS-CoV-2" OR "SARS-CoV2" OR SARSCoV2 OR "severe acute respiratory syndrome" OR sars OR influenza OR flu OR h1n1)

Supplementary Note 2. Preprint search strategies

bioRxiv and medRxiv – 2020/05/27 – 4,000 records

1. (covid19 or "covid 19" or wuhan coronavirus) and (artificial intelligence or support vector or expert system or fuzzy logic)
2. (covid19 or "covid 19" or wuhan coronavirus) and (natural language or neural network or machine learning or deep learning)
3. (covid19 or "covid 19" or wuhan coronavirus) and (language processing or bayesian or random forest)
4. (covid19 or "covid 19" or wuhan coronavirus) and (computer vision or multiple kernel or multi kernel or multikernel or knn)
5. (covid19 or "covid 19" or wuhan coronavirus) and (rnn or nearest neighbor or nearest neighbour or kmeans or k means)
6. (covid19 or "covid 19" or wuhan coronavirus) and (ensemble learning or ensemble model or seir or sir or susceptible exposed)
7. (covid19 or "covid 19" or wuhan coronavirus) and (susceptible infectious or kalman filter or data simulation)
8. (covid19 or "covid 19" or wuhan coronavirus) and (structured kernel or gaussian process or transfer learning or state space)
9. (covid19 or "covid 19" or wuhan coronavirus) and (non-parametric method or nonparametic method or graph convolutional)
10. (covid19 or "covid 19" or wuhan coronavirus) and (causal inference or counterfactual estimation or counterfactual simulation)
11. (covid19 or "covid 19" or wuhan coronavirus) and (adversarial network or trial enrichment or trial simulation)

Supplementary Note 3. Google search strategies

1. Covid AND artificial intelligence
2. Covid AND machine learning
3. Covid AND deep learning
4. Covid AND natural language processing
5. Covid AND computer vision
6. Covid AND data simulation
7. Covid AND SIR model (included results for SEIR model)
8. Covid AND data modeling

Supplementary Table 1. Inclusion and exclusion criteria

**Include**	**Exclude**
Pandemics<br>- Coronavirus disease 2019 (COVID-19)<br>- 2009 pandemic influenza A subtype H1N1<br>- Other<br>Global outbreak of severe acute respiratory syndrome (SARS)	Non-respiratory global outbreaks<br>- HIV/AIDS<br>- Hepatitis C<br>- Malaria<br>- Zika<br>- Ebola<br>- Other
Artificial intelligence (see *Glossary* in Box 1 for models)	Molecular docking, genome sequencing, or applications in robotics
Health policy, public health, and clinical practice	Public opinion, vaccine uptake or vaccine-related adverse events
Human populations	Animal populations
Peer-reviewed articles, preprints and conference proceedings describing original research or structured reviews. Grey literature describing novel applications of AI for pandemic preparedness or response.	Not published in the English language

Supplementary Table 2. Data abstraction for in-depth review of studies that used machine learning models

First Author Name	Release Year	Manuscript Title	Publication Status*	Relevant Use Case(s)	Disease	Region(s)	Purpose 1	Purpose 2	ML Model Type(s)	Outcomes for Purpose 1	Outcomes for Purpose 2	EHR Data	CT or X-Ray	Social Media Data	Publicly-Available Data
Li, S.	2020	The Impact of COVID-19 Epidemic Declaration on Psychological Consequences: A Study on Active Weibo Users	Peer-reviewed	Emerging Areas Beyond Management of Infection	COVID-19	China	Detection		NLP	Mental health status		No	No	Yes	Yes
Tummers, J.	2020	Coronaviruses and people with intellectual disability: an exploratory data analysis	Peer-reviewed	Emerging Areas Beyond Management of Infection	COVID-19	Multiple countries	Detection		K-means clustering	Themes from the literature		No	No	No	Yes
Awasthi, R.	2020	CovidNLP: A Web Application for Distilling Systemic Implications of COVID-19 Pandemic with Natural Language Processing	Preprint	Emerging Areas Beyond Management of Infection	COVID-19	Multiple countries	Summarization		NLP; NN	Relevant literature		No	No	No	Yes
Joshi, B.	2020	deepMINE - Natural Language Processing based Automatic Literature Mining and Research Summarization for Early-Stage Comprehension in Pandemic Situations specifically for COVID-19	Preprint	Emerging Areas Beyond Management of Infection	COVID-19	Multiple countries	Summarization		NLP	Relevant literature		No	No	No	Yes
Wagner, T.	2020	Real-time biomedical knowledge synthesis of the exponentially growing world wide web using unsupervised neural networks	Preprint	Emerging Areas Beyond Management of Infection	COVID-19	Multiple countries	Summarization		NN	Relevant literature		No	No	No	Yes
Zhao, Y.	2020	Applying Lexical Link Analysis to Discover Insights from Public Information on COVID-19	Preprint	Emerging Areas Beyond Management of Infection	COVID-19	Multiple countries	Detection		NLP	Themes from the literature		No	No	No	Yes
Al-qaness, M. A. A.	2020	Optimization method for forecasting confirmed cases of COVID-19 in China	Peer-reviewed	Forecasting of Infectious Disease Dynamics and Effects of Interventions	COVID-19	China; USA	Prediction		Fuzzy NN; k-NN; NN; SVM	Infections		No	No	No	Yes
Ayyoubzadeh, S. M.	2020	Predicting COVID-19 Incidence Through Analysis of Google Trends Data in Iran: Data Mining and Deep Learning Pilot Study	Peer-reviewed	Forecasting of Infectious Disease Dynamics and Effects of Interventions	COVID-19	Iran	Prediction		RNN	Infections		No	No	No	Yes
Fong, S. J.	2020	Finding an Accurate Early Forecasting Model from Small Dataset: A Case of 2019-nCoV Novel Coronavirus Outbreak	Peer-reviewed	Forecasting of Infectious Disease Dynamics and Effects of Interventions	COVID-19	China	Prediction		DT; Polynomial NN; SVM	Suspected infections		No	No	No	Yes

Pirouz, B.	2020	Investigating a Serious Challenge in the Sustainable Development Process: Analysis of Confirmed cases of COVID-19 (New Type of Coronavirus) Through a Binary Classification Using Artificial Intelligence and Regression Analysis	Peer-reviewed	Forecasting of Infectious Disease Dynamics and Effects of Interventions	COVID-19	China	Estimation		Polynomial NN	Infections		No	No	No	Yes
Araujo, M. B.	2020	Spread of SARS-CoV-2 Coronavirus likely to be constrained by climate	Preprint	Forecasting of Infectious Disease Dynamics and Effects of Interventions	COVID-19	Multiple countries	Estimation	Prediction	DT; GBT; MLP; RF	Climate suitability for COVID-19 outbreaks	Same as outcome for purpose 1	No	No	No	Yes
Basu, S.	2020	Going by the Numbers: Learning and Modeling COVID-19 Disease Dynamics	Preprint	Forecasting of Infectious Disease Dynamics and Effects of Interventions	COVID-19	Multiple countries	Prediction		RNN	Infections, deaths, and effects of NPIs		No	No	No	Yes
Dandekar, R.	2020	Quantifying the effect of quarantine control in Covid-19 infectious spread using machine learning	Preprint	Forecasting of Infectious Disease Dynamics and Effects of Interventions	COVID-19	Multiple countries	Prediction		NN	Infections, and effects of NPIs		No	No	No	Yes
Direkoglu, C.	2020	Worldwide and Regional Forecasting of Coronavirus (Covid-19) Spread using a Deep Learning Model	Preprint	Forecasting of Infectious Disease Dynamics and Effects of Interventions	COVID-19	Multiple countries	Prediction		RNN	Infections, and deaths		No	No	No	Yes
Distante, C.	2020	Forecasting Covid-19 Outbreak Progression in Italian Regions: A model based on neural network training from Chinese data	Preprint	Forecasting of Infectious Disease Dynamics and Effects of Interventions	COVID-19	Italy	Prediction		AE	Infections		No	No	No	Yes
Dutta, S.	2020	Machine Learning Approach for Confirmation of COVID-19 Cases: Positive, Negative, Death and Release	Preprint	Forecasting of Infectious Disease Dynamics and Effects of Interventions	COVID-19	South Korea	Prediction		RNN	Infections, and deaths		No	No	No	Yes
Ge, Q.	2020	A Noel Intervention Recurrent autoencoder for real time forecasting and non-pharmaceutical intervention selection to curb the spread of Covid-19 in the world	Preprint	Forecasting of Infectious Disease Dynamics and Effects of Interventions	COVID-19	Multiple countries	Prediction		RNN	Infections, and effects of NPIs		No	No	No	Yes
Ghosal, S.	2020	Impact of complete lock-down on total infection and death rates: A hierarchical cluster analysis	Preprint	Forecasting of Infectious Disease Dynamics and Effects of Interventions	COVID-19	Multiple countries	Estimation		K-means clustering	Effects of NPIs		No	No	No	Yes

Hu, Z.	2020	Evaluating the effect of public health intervention on the global-wide spread trajectory of Covid-19	Preprint	Forecasting of Infectious Disease Dynamics and Effects of Interventions	COVID-19	Multiple countries	Prediction		AE	Infections, and effects of NPIs		No	No	No	Yes
Hu, Z.	2020	Spread of Covid-19 in the United States is controlled	Preprint	Forecasting of Infectious Disease Dynamics and Effects of Interventions	COVID-19	USA	Prediction		AE	Infections, and effects of NPIs		No	No	No	Yes
Hu, Z.	2020	Artificial intelligence forecasting of COVID-19 in China	Preprint	Forecasting of Infectious Disease Dynamics and Effects of Interventions	COVID-19	China	Prediction		AE	Infections		No	No	No	Yes
Huang, C.-J.	2020	Multiple-Input Deep Convolutional Neural Network Model for COVID-19 Forecasting in China	Preprint	Forecasting of Infectious Disease Dynamics and Effects of Interventions	COVID-19	China	Prediction		CNN; MLP; RNN	Infections		No	No	No	Yes
Huang, C.-J.	2020	Novel Spatiotemporal Feature Extraction Parallel Deep Neural Network for Forecasting Confirmed Cases of Coronavirus Disease 2019	Preprint	Forecasting of Infectious Disease Dynamics and Effects of Interventions	COVID-19	Germany; Italy; Spain	Prediction		CNN; RNN	Infections		No	No	No	Yes
Ibrahim, M. R.	2020	Variational-LSTM Autoencoder to forecast the spread of coronavirus across the globe	Preprint	Forecasting of Infectious Disease Dynamics and Effects of Interventions	COVID-19	Multiple countries	Prediction		AE; RNN	Infections		No	No	No	Yes
Kafieh, R.	2020	COVID-19 in Iran: A Deeper Look Into The Future	Preprint	Forecasting of Infectious Disease Dynamics and Effects of Interventions	COVID-19	Multiple countries	Prediction		MLP; RF; RNN	Infections, deaths, and effects of NPIs		No	No	No	Yes
Khan, H. R.	2020	Countries are Clustered but Number of Tests is not Vital to Predict Global COVID-19 Confirmed Cases: A Machine Learning Approach	Preprint	Forecasting of Infectious Disease Dynamics and Effects of Interventions	COVID-19	Multiple countries	Prediction		DT	Infections		No	No	No	Yes
Kolozsvari, L. R.	2020	Predicting the epidemic curve of the coronavirus (SARS-CoV-2) disease (COVID-19) using artificial intelligence	Preprint	Forecasting of Infectious Disease Dynamics and Effects of Interventions	COVID-19	Multiple countries	Prediction		RNN	Infections		No	No	No	Yes
Kumar, A.	2020	Preparedness and Mitigation by projecting the risk against COVID-19 transmission using Machine Learning Techniques	Preprint	Forecasting of Infectious Disease Dynamics and Effects of Interventions	COVID-19	India	Prediction		DT; SVM	Criticality index of low, moderate, and high risk		No	No	No	Yes

Li, M.	2020	Predicting the epidemic trend of COVID-19 in China and across the world using the machine learning approach	Preprint	Forecasting of Infectious Disease Dynamics and Effects of Interventions	COVID-19	Multiple countries	Prediction		Proprietary	Infections, and deaths		No	No	No	Yes
Mehta, M.	2020	Early Stage Prediction of US County Vulnerability to the COVID-19 Pandemic	Preprint	Forecasting of Infectious Disease Dynamics and Effects of Interventions	COVID-19	USA	Prediction		GBT	Infections, and vulnerability to COVID-19		No	No	No	Yes
Ogundokun, R. O.	2020	Machine Learning Prediction for COVID 19 Pandemic in India	Preprint	Forecasting of Infectious Disease Dynamics and Effects of Interventions	COVID-19	India	Prediction		MLP; SVM	Infections		No	No	No	Yes
Pinter, G.	2020	COVID-19 Pandemic Prediction for Hungary; a Hybrid Machine Learning Approach	Preprint	Forecasting of Infectious Disease Dynamics and Effects of Interventions	COVID-19	Hungary	Prediction		Fuzzy NN; MLP	Infections, and deaths		No	No	No	Yes
Suzuki, Y.	2020	Machine learning model estimating number of COVID-19 infection cases over coming 24 days in every province of South Korea (XGBoost and MultiOutputRegressor)	Preprint	Forecasting of Infectious Disease Dynamics and Effects of Interventions	COVID-19	South Korea	Prediction		GBT	Infections		No	No	No	Yes
Tian, T.	2020	COVID-Net: A deep learning based and interpretable predication model for the county-wise trajectories of COVID-19 in the United States	Preprint	Forecasting of Infectious Disease Dynamics and Effects of Interventions	COVID-19	USA	Prediction		RNN	Infections, and deaths		No	No	No	Yes
Uhlig, S.	2020	Modeling projections for COVID-19 pandemic by combining epidemiological, statistical, and neural network approaches	Preprint	Forecasting of Infectious Disease Dynamics and Effects of Interventions	COVID-19	Multiple countries	Prediction		NN	Infections		No	No	No	Yes
Watson, G. L.	2020	Fusing a Bayesian case velocity model with random forest for predicting COVID-19 in the U.S.	Preprint	Forecasting of Infectious Disease Dynamics and Effects of Interventions	COVID-19	USA	Prediction		RF	Infections, and deaths		No	No	No	Yes
Yu, Y.	2020	COVID-19 Asymptomatic Infection Estimation	Preprint	Forecasting of Infectious Disease Dynamics and Effects of Interventions	COVID-19	Multiple countries	Estimation	Prediction	RNN	Asymptomatic infections	Infections, and effects of NPIs	No	No	No	Yes
Zhao, Z.	2020	How well can we forecast the COVID-19 pandemic with curve fitting and recurrent neural networks?	Preprint	Forecasting of Infectious Disease Dynamics and Effects of Interventions	COVID-19	USA	Prediction		RNN	Infections		No	No	No	Yes

Lopez, D.	2015	Assessment of Vaccination Strategies Using Fuzzy Multi-criteria Decision Making	Conference Proceeding	Forecasting of Infectious Disease Dynamics and Effects of Interventions	H1N1	India	Prediction		Fuzzy logic	Optimal vaccination strategies		No	No	No	Yes
Mei, S.	2014	Individual Decision Making Can Drive Epidemics: A Fuzzy Cognitive Map Study	Peer-reviewed	Forecasting of Infectious Disease Dynamics and Effects of Interventions	H1N1	China	Prediction		Fuzzy logic	Infections, and effects of NPIs		No	No	No	No
Tessmer, H. L.	2018	Can Machines Learn Respiratory Virus Epidemiology?: A Comparative Study of Likelihood-Free Methods for the Estimation of Epidemiological Dynamics	Peer-reviewed	Forecasting of Infectious Disease Dynamics and Effects of Interventions	H1N1	USA	Estimation		CNN; MLP; RNN	Reproductive number		No	No	No	Yes
Aviso, K. B.	2018	Allocating human resources in organizations operating under crisis conditions: A fuzzy input-output optimization modeling framework	Peer-reviewed	Forecasting of Infectious Disease Dynamics and Effects of Interventions	Hypothetical	USA	Prediction		Fuzzy logic	Optimal allocation of available personnel		No	No	No	Yes
Jiang, C. L.	2005	A prediction method with more precision on SARS epidemic transmission	Conference Proceeding	Forecasting of Infectious Disease Dynamics and Effects of Interventions	SARS	China	Prediction		MLP	Infections		No	No	No	Yes
Bai, Y. P.	2005	Prediction of SARS epidemic by BP neural networks with online prediction strategy	Peer-reviewed	Forecasting of Infectious Disease Dynamics and Effects of Interventions	SARS	China	Prediction		MLP	Infections, death, hospitalization, and effects of NPIs		No	No	No	Yes
Elavarasan, R. M.	2020	Restructured society and environment: A review on potential technological strategies to control the COVID-19 pandemic	Peer-reviewed	Multiple Use Cases (Review)	COVID-19	Various - Review	Various - Review		Various - Review	Various - Review		No	No	No	Yes
Vaishya, R.	2020	Artificial Intelligence (AI) applications for COVID-19 pandemic	Peer-reviewed	Multiple Use Cases (Review)	COVID-19	Various - Review	Various - Review		Various - Review	Various - Review		No	No	No	Yes
Shuja, J.	2020	COVID-19 Datasets: A Survey and Future Challenges	Preprint	Multiple Use Cases (Review)	COVID-19	Various - Review	Various - Review		Various - Review	Various - Review		No	No	No	Yes
Al-Najjar, H.	2020	A classifier prediction model to predict the status of Coronavirus COVID-19 patients in South Korea	Peer-reviewed	Prognosis of Illness and Response to Treatment	COVID-19	South Korea	Prediction		NN	Death		No	No	No	Yes
Gong, J.	2020	A Tool to Early Predict Severe Corona Virus Disease 2019 (COVID-19): A Multicenter Study using the Risk Nomogram in Wuhan and Guangdong, China	Peer-reviewed	Prognosis of Illness and Response to Treatment	COVID-19	China	Prediction		DT; RF; SVM	Disease severity		Unclear	No	No	No
Jiang, X. G.	2020	Towards an Artificial Intelligence Framework for Data-Driven Prediction of Coronavirus Clinical Severity	Peer-reviewed	Prognosis of Illness and Response to Treatment	COVID-19	China	Prediction		DT; k-NN; RF; SVM	Disease severity		Unclear	No	No	No

Bai, X.	2020	Predicting COVID-19 malignant progression with AI techniques	Preprint	Prognosis of Illness and Response to Treatment	COVID-19	China	Prediction		MLP; RNN	Disease severity		Unclear	CT	No	No
Barda, N.	2020	Performing risk stratification for COVID-19 when individual level data is not available - the experience of a large healthcare organization	Preprint	Prognosis of Illness and Response to Treatment	COVID-19	Israel	Prediction		GBT	Death		Unclear	No	No	No
Burian, E.	2020	Intensive care risk estimation in COVID-19 pneumonia based on clinical and imaging parameters: experiences from the Munich cohort	Preprint	Prognosis of Illness and Response to Treatment	COVID-19	Germany	Prediction		RF	Intensive care admission		Unclear	CT	No	No
Das, A.	2020	Predicting community mortality risk due to CoVID-19 using machine learning and development of a prediction tool	Preprint	Prognosis of Illness and Response to Treatment	COVID-19	South Korea	Prediction		GBT; k-NN; RF; SVM	Death		No	No	No	Yes
DeCaprio, D.	2020	Building a COVID-19 Vulnerability Index	Preprint	Prognosis of Illness and Response to Treatment	COVID-19	USA	Prediction		GBT	Severe complications due to COVID-19 if infected		No	No	No	Yes
Duchesne, S.	2020	Tracking and Predicting COVID-19 Radiological Trajectory using Deep Learning on Chest X-Rays: Initial Accuracy Testing	Preprint	Prognosis of Illness and Response to Treatment	COVID-19	Multiple countries	Prediction		CNN	Disease severity		No	X-ray	No	Yes
Fakhfakh, M.	2020	ProgNet: Covid-19 prognosis using recurrent and convolutional neural networks	Preprint	Prognosis of Illness and Response to Treatment	COVID-19	Multiple countries	Prediction		CNN; RNN	Disease severity		No	X-ray	No	Yes
Heldt, F. S.	2020	Early risk assessment for COVID-19 patients from emergency department data using machine learning	Preprint	Prognosis of Illness and Response to Treatment	COVID-19	England	Prediction		GBT; RF	Death, intensive care admission, and need for mechanical ventilation		Yes	No	No	No
Hu, C.	2020	Early prediction of mortality risk among severe COVID-19 patients using machine learning	Preprint	Prognosis of Illness and Response to Treatment	COVID-19	China	Prediction		RF	Death		Unclear	No	No	No
Izquierdo, J. L.	2020	Clinical Characteristics and Prognostic Factors for ICU Admission of Patients With COVID-19 using Machine Learning and Natural Language Processing	Preprint	Prognosis of Illness and Response to Treatment	COVID-19	Spain	Prediction		Proprietary extraction technology (including CNN, NLP, NN); DT	Intensive care admission		Yes	No	No	No
Lassau, N.	2020	AI-based multi-modal integration of clinical characteristics, lab tests and chest CTs improves COVID-19 outcome prediction of hospitalized patients	Preprint	Prognosis of Illness and Response to Treatment	COVID-19	France	Prediction		CNN	Disease severity, need for mechanical ventilation, intensive care admission, and death		Unclear	CT	No	No

Liew, C.	2020	A chest radiography-based artificial intelligence deep-learning model to predict severe Covid-19 patient outcomes: the CAPE (Covid-19 AI Predictive Engine) Model	Preprint	Prognosis of Illness and Response to Treatment	COVID-19	Redacted for journal review	Prediction		CNN	Death, and intensive care admission		Yes	X-ray	No	No
Pourhomayoun, M.	2020	Predicting Mortality Risk in Patients with COVID-19 Using Artificial Intelligence to Help Medical Decision-Making	Preprint	Prognosis of Illness and Response to Treatment	COVID-19	Multiple countries	Prediction		DT; k-NN; NN; RF; SVM	Death		No	No	No	Yes
Qi, X.	2020	Machine learning-based CT radiomics model for predicting hospital stay in patients with pneumonia associated with SARS-CoV-2 infection: A multicenter study	Preprint	Prognosis of Illness and Response to Treatment	COVID-19	China	Prediction		CNN; RF	Length of hospital stay		Unclear	CT	No	No
Sarkar, J.	2020	A Machine Learning Model Reveals Older Age and Delayed Hospitalization as Predictors of Mortality in Patients with COVID-19	Preprint	Prognosis of Illness and Response to Treatment	COVID-19	Multiple countries	Prediction		RF	Death		No	No	No	Yes
Singh, K.	2020	Validating a Widely Implemented Deterioration Index Model Among Hospitalized COVID-19 Patients	Preprint	Prognosis of Illness and Response to Treatment	COVID-19	USA	Prediction		Proprietary	Intensive care admission, mechanical ventilation, and death		Yes	No	No	No
Vaid, A.	2020	Machine Learning to Predict Mortality and Critical Events in COVID-19 Positive New York City Patients	Preprint	Prognosis of Illness and Response to Treatment	COVID-19	USA	Prediction		GBT	Death, intubation, and discharge to hospice care		Yes	No	No	No
Wollenstein-Betech, S.	2020	Personalized Predictive Models for Symptomatic COVID-19 Patients Using Basic Preconditions: Hospitalizations, Mortality, and the Need for an ICU or Ventilator	Preprint	Prognosis of Illness and Response to Treatment	COVID-19	Mexico	Prediction		GBT; RF; SVM	Hospitalization, death, intensive care admission, and ventilator use		Unclear	No	No	Yes
Wu, G.	2020	Development of a Clinical Decision Support System for Severity Risk Prediction and Triage of COVID-19 Patients at Hospital Admission: an International Multicenter Study	Preprint	Prognosis of Illness and Response to Treatment	COVID-19	Belgium; China; Italy	Prediction		RF	Disease severity		Yes	CT	No	Yes
Yadaw, A.	2020	Clinical predictors of COVID-19 mortality	Preprint	Prognosis of Illness and Response to Treatment	COVID-19	USA	Prediction		GBT; RF; SVM	Death		Yes	No	No	No
Yan, L.	2020	A machine learning-based model for survival prediction in patients with severe COVID-19 infection	Preprint	Prognosis of Illness and Response to Treatment	COVID-19	China	Prediction		GBT	Death		Yes	No	No	No
Hegde, C.	2020	AutoTriage - An Open Source Edge Computing Raspberry Pi-based Clinical Screening System	Preprint	Real-time Detection of Influenza-like Illness	COVID-19	Multiple countries	Detection		CNN; k-NN; SVM	Symptoms		No	No	No	Yes

Imran, A.	2020	AI4COVID-19: AI Enabled Preliminary Diagnosis for COVID-19 from Cough Samples via an App	Preprint	Real-time Detection of Influenza-like Illness	COVID-19	Not reported - Authors from USA	Detection		CNN; SVM	Disease		No	No	No	Yes
Ng, E. Y. K.	2006	ANN-based mapping of febrile subjects in mass thermogram screening: facts and myths	Peer-reviewed	Real-time Detection of Influenza-like Illness	SARS	Singapore	Detection		MLP; SLP; SOM	Symptoms		No	No	No	No
Ng, E. Y. K.	2005	Classification of human facial and aural temperature using neural networks and IR fever scanner: A responsible second look	Peer-reviewed	Real-time Detection of Influenza-like Illness	SARS	Singapore	Detection		MLP; SLP; SOM	Symptoms		No	No	No	No
Ng, E. Y. K.	2005	Is thermal scanner losing its bite in mass screening of fever due to SARS?	Peer-reviewed	Real-time Detection of Influenza-like Illness	SARS	Singapore	Detection		MLP; SLP; SOM	Symptoms		No	No	No	No
Quek, C.	2010	A Cognitive Interpretation of Thermographic Images Using Novel Fuzzy Learning Semantic Memories	Peer-reviewed	Real-time Detection of Influenza-like Illness	SARS	Singapore	Detection		Fuzzy NN; MLP	Symptoms		No	No	No	No
Quek, C.	2010	A novel brain-inspired neural cognitive approach to SARS thermal image analysis	Peer-reviewed	Real-time Detection of Influenza-like Illness	SARS	Singapore	Detection		Fuzzy NN; MLP	Symptoms		No	No	No	No
Sun, G. H.	2017	Applications of Infrared Thermography for Noncontact and Noninvasive Mass Screening of Febrile International Travelers at Airport Quarantine Stations	Peer-reviewed	Real-time Detection of Influenza-like Illness	SARS	Japan	Detection		K-means clustering; k-NN; SOM; SVM	Symptoms		No	No	No	No
Zhang, L.	2020	An Interactive COVID-19 Mobility Impact and Social Distancing Analysis Platform	Preprint	Real-time Monitoring of Adherence to Public Health Recommendations	COVID-19	USA	Estimation		NN	Adherence to NPIs		No	No	No	No
Golder, S.	2020	Extending A Chronological and Geographical Analysis of Personal Reports of COVID-19 on Twitter to England, UK	Preprint	Surveillance and Outbreak Detection	COVID-19	UK	Surveillance		NLP; Transformer NN	Potential exposure to COVID-19		No	No	Yes	Yes
Klein, A. Z.	2020	A Chronological and Geographical Analysis of Personal Reports of COVID-19 on Twitter	Preprint	Surveillance and Outbreak Detection	COVID-19	USA	Surveillance		NLP; Transformer NN	Potential exposure to COVID-19		No	No	Yes	Yes
Aramaki, E.	2011	Twitter catches the flu: detecting influenza epidemics using Twitter	Conference Proceeding	Surveillance and Outbreak Detection	H1N1	Japan	Surveillance		NLP; SVM	Disease		No	No	Yes	Yes
Culotta, A.	2010	Towards detecting influenza epidemics by analyzing Twitter messages	Conference Proceeding	Surveillance and Outbreak Detection	H1N1	USA	Surveillance		NLP	Symptoms		No	No	Yes	Yes
Jain, V. K.	2015	An Effective Approach to Track Levels of Influenza-A (H1N1) Pandemic in India Using Twitter	Conference Proceeding	Surveillance and Outbreak Detection	H1N1	India	Surveillance		DT; NLP; RF; SVM	Symptoms		No	No	Yes	Yes

Lampos, V.	2010	Tracking the flu pandemic by monitoring the social web	Conference Proceeding	Surveillance and Outbreak Detection	H1N1	UK	Surveillance		NLP	Symptoms		No	No	Yes	Yes
Pei, J.	2013	Improving prediction accuracy of influenza-like illnesses in hospital emergency departments	Conference Proceeding	Surveillance and Outbreak Detection	H1N1	Canada	Surveillance		NN; DT; SVM	Symptoms		Unclear	No	No	No
Collier, N.	2011	OMG U got flu? Analysis of shared health messages for bio-surveillance	Peer-reviewed	Surveillance and Outbreak Detection	H1N1	USA	Surveillance		NLP; SVM	Disease		No	No	Yes	Yes
Jain, V. K.	2018	Rough set based intelligent approach for identification of H1N1 suspect using social media	Peer-reviewed	Surveillance and Outbreak Detection	H1N1	India	Surveillance		DT; Fuzzy logic; RF; SVM	Disease		No	No	Yes	Yes
López Pineda, A.	2015	Comparison of machine learning classifiers for influenza detection from emergency department free-text reports	Peer-reviewed	Surveillance and Outbreak Detection	H1N1	USA	Surveillance		MLP; NLP; RF; SVM	Disease		Yes	No	No	No
Signorini, A.	2011	The Use of Twitter to Track Levels of Disease Activity and Public Concern in the U.S. during the Influenza A H1N1 Pandemic	Peer-reviewed	Surveillance and Outbreak Detection	H1N1	USA	Surveillance		SVM	Symptoms		No	No	Yes	Yes
Huang, H.	2016	(SH)-H-3: A Symptom Surveillance System in High Spatial Resolution using Smartphones	Peer-reviewed	Surveillance and Outbreak Detection	Hypothetical	USA	Surveillance		k-NN	Symptoms		No	No	No	Yes
Damianos, L. E.	2004	MiTAP for SARS detection	Conference Proceeding	Surveillance and Outbreak Detection	SARS	USA	Surveillance		NLP	Disease		No	No	Yes	Yes
Al-garadi, M. A.	2016	Using online social networks to track a pandemic: A systematic review	Peer-reviewed	Surveillance and Outbreak Detection (Review)	H1N1, pandemics	Various - Review	Surveillance		Various - Review	Various - Review		No	No	No	Yes
Chamberlain, S. D.	2020	Real-time detection of COVID-19 epicenters within the United States using a network of smart thermometers	Preprint	Surveillance and Outbreak Detection; Forecasting of Infectious Disease Dynamics and Effects of Interventions	COVID-19	USA	Surveillance	Prediction	k-NN	Symptoms	Same as outcome for purpose 1	No	No	No	No
Achrekar, H.	2012	Twitter Improves Seasonal Influenza Prediction	Conference Proceeding	Surveillance and Outbreak Detection; Forecasting of Infectious Disease Dynamics and Effects of Interventions	H1N1	USA	Surveillance	Prediction	DT; SVM	Symptoms	Same as outcome for purpose 1	No	No	Yes	Yes
Apostolopoulos, I. D.	2020	Covid-19: automatic detection from X-ray images utilizing transfer learning with convolutional neural networks	Peer-reviewed	Triage and Timely Diagnosis of Infections	COVID-19	Multiple countries	Detection		CNN	Disease		No	X-ray	No	Yes

Bai, H. X.	2020	AI Augmentation of Radiologist Performance in Distinguishing COVID-19 from Pneumonia of Other Etiology on Chest CT	Peer-reviewed	Triage and Timely Diagnosis of Infections	COVID-19	China; USA	Detection		CNN	Disease		Yes	CT	No	No
Cheng, Z.	2020	Quantitative computed tomography of the coronavirus disease 2019 (COVID-19) pneumonia	Peer-reviewed	Triage and Timely Diagnosis of Infections	COVID-19	China	Estimation		CNN	Disease severity		Unclear	CT	No	No
Hurt, B.	2020	Deep Learning Localization of Pneumonia: 2019 Coronavirus (COVID-19) Outbreak	Peer-reviewed	Triage and Timely Diagnosis of Infections	COVID-19	China; USA	Estimation		CNN	Disease severity		No	X-ray	No	Yes
Li, L.	2020	Artificial Intelligence Distinguishes COVID-19 from Community Acquired Pneumonia on Chest CT	Peer-reviewed	Triage and Timely Diagnosis of Infections	COVID-19	Not reported - Authors from China	Detection		CNN	Disease		Unclear	CT	No	No
Singh, D.	2020	Classification of COVID-19 patients from chest CT images using multi-objective differential evolution-based convolutional neural networks	Peer-reviewed	Triage and Timely Diagnosis of Infections	COVID-19	Not reported - Authors in India	Detection		CNN; Fuzzy NN; NN	Disease		Unclear	CT	No	Unclear
Ucar, F.	2020	COVIDiagnosis-Net: Deep Bayes-SqueezeNet based diagnosis of the coronavirus disease 2019 (COVID-19) from X-ray images	Peer-reviewed	Triage and Timely Diagnosis of Infections	COVID-19	Multiple countries	Detection		CNN	Disease		No	X-ray	No	Yes
Abbas, A.	2020	Classification of COVID-19 in chest X-ray images using DeTraC deep convolutional neural network	Preprint	Triage and Timely Diagnosis of Infections	COVID-19	Multiple countries	Detection		CNN	Disease		No	X-ray	No	Yes
Al-karawi, D.	2020	Machine Learning Analysis of Chest CT Scan Images as a Complementary Digital Test of Coronavirus (COVID-19) Patients	Preprint	Triage and Timely Diagnosis of Infections	COVID-19	China	Detection		SVM	Disease		No	CT	No	Yes
Al-karawi, D.	2020	AI based Chest X-Ray (CXR) Scan Texture Analysis Algorithm for Digital Test of COVID-19 Patients	Preprint	Triage and Timely Diagnosis of Infections	COVID-19	Multiple countries	Detection		SVM	Disease		No	X-ray	No	Yes
Asif, S.	2020	Automatic Detection of COVID-19 Using X-ray Images with Deep Convolutional Neural Networks and Machine Learning	Preprint	Triage and Timely Diagnosis of Infections	COVID-19	Multiple countries	Detection		CNN	Disease		No	X-ray	No	Yes
Barstugan, M.	2020	Coronavirus (COVID-19) classification using CT images by machine learning methods	Preprint	Triage and Timely Diagnosis of Infections	COVID-19	Italy	Detection		SVM	Disease		No	CT	No	Yes
Basu, S.	2020	Deep Learning for Screening COVID-19 using Chest X-Ray Images	Preprint	Triage and Timely Diagnosis of Infections	COVID-19	Multiple countries	Detection		CNN	Disease		No	X-ray	No	Yes
Borkowski, A. A.	2020	Using Artificial Intelligence for COVID-19 Chest X-ray Diagnosis	Preprint	Triage and Timely Diagnosis of Infections	COVID-19	Multiple countries	Detection		Proprietary	Disease		No	X-ray	No	Yes

Brinati, D.	2020	Detection of COVID-19 Infection from Routine Blood Exams with Machine Learning: a Feasibility Study	Preprint	Triage and Timely Diagnosis of Infections	COVID-19	Italy	Detection		DT; Extremely randomized trees; k-NN; RF; SVM	Disease		Unclear	No	No	No
Bukhari, S. U.	2020	The diagnostic evaluation of Convolutional Neural Network (CNN) for the assessment of chest X-ray of patients infected with COVID-19	Preprint	Triage and Timely Diagnosis of Infections	COVID-19	Multiple countries	Detection		CNN	Disease		No	X-ray	No	Yes
Castiglioni, I.	2020	Artificial intelligence applied on chest X-ray can aid in the diagnosis of COVID-19 infection: a first experience from Lombardy, Italy	Preprint	Triage and Timely Diagnosis of Infections	COVID-19	Italy	Detection		CNN	Disease		Unclear	X-ray	No	No
Chaganti, S.	2020	Automated Quantification of CT Patterns Associated with COVID-19 from Chest CT	Preprint	Triage and Timely Diagnosis of Infections	COVID-19	Multiple countries	Estimation		CNN	Disease severity		Unclear	CT	No	No
Chen, J.	2020	Deep learning-based model for detecting 2019 novel coronavirus pneumonia on high-resolution computed tomography: a prospective study	Preprint	Triage and Timely Diagnosis of Infections	COVID-19	China	Detection		CNN	Disease		Unclear	CT	No	No
Chen, Y.	2020	An Interpretable Machine Learning Framework for Accurate Severe vs Non-severe COVID-19 Clinical Type Classification	Preprint	Triage and Timely Diagnosis of Infections	COVID-19	China	Estimation		RF	Disease severity		Unclear	No	No	No
Chowdhury, M.	2020	Can AI help in screening Viral and COVID-19 pneumonia?	Preprint	Triage and Timely Diagnosis of Infections	COVID-19	Multiple countries	Detection		CNN	Disease		No	X-ray	No	Yes
de Freitas Barbosa, V. A.	2020	Heg.IA: An intelligent system to support diagnosis of Covid-19 based on blood tests	Preprint	Triage and Timely Diagnosis of Infections	COVID-19	Brazil	Detection		DT; MLP; RF; SVM	Disease		No	No	No	Yes
de Moraes Batista, A. F.	2020	COVID-19 diagnosis prediction in emergency care patients: a machine learning approach	Preprint	Triage and Timely Diagnosis of Infections	COVID-19	Brazil	Detection		GBT; NN; RF; SVM	Disease		Unclear	No	No	No
de Moura, J.	2020	Fully automatic deep convolutional approaches for the analysis of Covid-19 using chest X-ray images	Preprint	Triage and Timely Diagnosis of Infections	COVID-19	Multiple countries	Detection		CNN	Disease		No	X-ray	No	Yes
Elgendi, M.	2020	The Evaluation of Deep Neural Networks and X-Ray as a Practical Alternative for Diagnosis and Management of COVID-19	Preprint	Triage and Timely Diagnosis of Infections	COVID-19	Multiple countries	Detection		CNN	Disease		No	X-ray	No	Yes
Fan, D.-P.	2020	Inf-Net: Automatic COVID-19 Lung Infection Segmentation from CT Images	Preprint	Triage and Timely Diagnosis of Infections	COVID-19	Multiple countries	Detection		CNN	Image segmentation		No	CT	No	Yes
Feng, C.	2020	A Novel Triage Tool of Artificial Intelligence Assisted Diagnosis Aid System for Suspected COVID-19 pneumonia In Fever Clinics	Preprint	Triage and Timely Diagnosis of Infections	COVID-19	China	Detection		AdaBoost; DT	Disease		Unclear	No	No	No

Fu, M.	2020	Deep Learning-Based Recognizing COVID-19 and other Common Infectious Diseases of the Lung by Chest CT Scan Images	Preprint	Triage and Timely Diagnosis of Infections	COVID-19	China	Detection		CNN	Disease		Unclear	CT	No	No
Gomes, J. C.	2020	IKONOS: An intelligent tool to support diagnosis of Covid-19 by texture analysis of x-ray images	Preprint	Triage and Timely Diagnosis of Infections	COVID-19	Multiple countries	Detection		DT; MLP; RF; SVM	Disease		No	X-ray	No	Yes
Gozes, O.	2020	Rapid AI development cycle for the coronavirus (covid-19) pandemic: initial results for automated detection & patient monitoring using deep learning CT image analysis	Preprint	Triage and Timely Diagnosis of Infections	COVID-19	China; USA	Detection	Estimation	CNN; Proprietary	Disease	Disease severity	Unclear	CT	No	Yes
Gozes, O.	2020	Coronavirus detection and analysis on chest CT with deep learning	Preprint	Triage and Timely Diagnosis of Infections	COVID-19	Multiple countries	Detection	Estimation	CNN	Disease	Disease severity	Unclear	CT	No	Yes
Gueguim, K.	2020	A web-based Diagnostic Tool for COVID-19 Using Machine Learning on Chest Radiographs (CXR)	Preprint	Triage and Timely Diagnosis of Infections	COVID-19	Multiple countries	Detection		CNN	Disease		No	X-ray	No	Yes
Hassanien, A. E.	2020	Automatic X-ray COVID-19 Lung Image Classification System based on Multi-Level Thresholding and Support Vector Machine	Preprint	Triage and Timely Diagnosis of Infections	COVID-19	Multiple countries	Detection		SVM	Disease		No	X-ray	No	Yes
He, X.	2020	Sample-Efficient Deep Learning for COVID-19 Diagnosis Based on CT Scans	Preprint	Triage and Timely Diagnosis of Infections	COVID-19	Multiple countries	Detection		CNN	Disease		No	CT	No	Yes
Hu, R.	2020	Automated Diagnosis of COVID-19 Using Deep Learning and Data Augmentation on Chest CT	Preprint	Triage and Timely Diagnosis of Infections	COVID-19	Multiple countries	Detection		CNN	Disease		No	CT	No	Yes
Jin, C.	2020	Development and evaluation of an AI system for covid-19 diagnosis	Preprint	Triage and Timely Diagnosis of Infections	COVID-19	Multiple countries	Detection		CNN	Disease		Unclear	CT	No	Yes
Jin, S.	2020	AI-assisted CT imaging analysis for COVID-19 screening: Building and deploying a medical AI system in four weeks	Preprint	Triage and Timely Diagnosis of Infections	COVID-19	China	Detection		CNN	Disease		Unclear	CT	No	No
Khobahi, S.	2020	CoroNet: A Deep Network Architecture for Semi-Supervised Task-Based Identification of COVID-19 from Chest X-ray Images	Preprint	Triage and Timely Diagnosis of Infections	COVID-19	Multiple countries	Detection		AE; CNN	Disease		No	X-ray	No	Yes
Kumar, R.	2020	Accurate Prediction of COVID-19 using Chest X-Ray Images through Deep Feature Learning model with SMOTE and Machine Learning Classifiers	Preprint	Triage and Timely Diagnosis of Infections	COVID-19	Multiple countries	Detection		AdaBoost; CNN; DT; GBT; k-NN; RF	Disease		No	X-ray	No	Yes
Kumar, S.	2020	Deep Transfer Learning-based COVID-19 prediction using Chest X-rays	Preprint	Triage and Timely Diagnosis of Infections	COVID-19	Multiple countries	Detection		CNN	Disease		No	X-ray	No	Yes

Li, Z.	2020	From Community Acquired Pneumonia to COVID-19: A Deep Learning Based Method for Quantitative Analysis of COVID-19 on thick-section CT Scans	Preprint	Triage and Timely Diagnosis of Infections	COVID-19	China	Estimation		CNN	Disease severity		Unclear	CT	No	No
Liu, B.	2020	Assisting Scalable Diagnosis Automatically via CT Images in the Combat against COVID-19	Preprint	Triage and Timely Diagnosis of Infections	COVID-19	China	Detection		CNN	Disease		Unclear	CT	No	No
Lucius, M.	2020	Robust COVID-19-Related Condition Classification Network	Preprint	Triage and Timely Diagnosis of Infections	COVID-19	Multiple countries	Detection		CNN	Disease		No	X-ray	No	Yes
Majeed, T.	2020	Covid-19 Detection using CNN Transfer Learning from X-ray Images	Preprint	Triage and Timely Diagnosis of Infections	COVID-19	Multiple countries	Detection		CNN	Disease		No	X-ray	No	Yes
Makris, A.	2020	COVID-19 detection from chest X-Ray images using Deep Learning and Convolutional Neural Networks	Preprint	Triage and Timely Diagnosis of Infections	COVID-19	Multiple countries	Detection		CNN	Disease		No	X-ray	No	Yes
Martin, A.	2020	An artificial intelligence-based first-line defence against COVID-19: digitally screening citizens for risks via a chatbot	Preprint	Triage and Timely Diagnosis of Infections	COVID-19	Multiple countries	Detection		Proprietary	Disease		No	No	No	No
Medhi, K.	2020	Automatic Detection of COVID-19 Infection from Chest X-ray using Deep Learning	Preprint	Triage and Timely Diagnosis of Infections	COVID-19	China	Detection		CNN	Disease		No	X-ray	No	Yes
Mei, X.	2020	Artificial intelligence-enabled rapid diagnosis of COVID-19 patients	Preprint	Triage and Timely Diagnosis of Infections	COVID-19	China	Detection		CNN; MLP; RF; SVM	Disease		Unclear	CT	No	No
Ozsahin, I.	2020	Differentiating COVID-19 from other types of pneumonia with convolutional neural networks	Preprint	Triage and Timely Diagnosis of Infections	COVID-19	Multiple countries	Detection		CNN	Disease		No	X-ray	No	Yes
Ozturk, S.	2020	Classification of Coronavirus Images using Shrunken Features	Preprint	Triage and Timely Diagnosis of Infections	COVID-19	Multiple countries	Detection		AE; k-NN; SVM	Disease		No	CT and X-ray	No	Yes
Rajaraman, S.	2020	Training deep learning algorithms with weakly labeled pneumonia chest X-ray data for COVID-19 detection	Preprint	Triage and Timely Diagnosis of Infections	COVID-19	Multiple countries	Detection		CNN	Disease		No	X-ray	Yes	Yes
Rehman, A.	2020	Improving Coronavirus (COVID-19) Diagnosis using Deep Transfer Learning	Preprint	Triage and Timely Diagnosis of Infections	COVID-19	Multiple countries	Detection		CNN	Disease		No	CT and X-ray	No	Yes
Shan, F.	2020	Lung infection quantification of covid-19 in CT images with deep learning	Preprint	Triage and Timely Diagnosis of Infections	COVID-19	China	Estimation		CNN	Disease severity		Unclear	CT	No	No
Shi, F.	2020	Large-scale screening of covid-19 from community acquired pneumonia using infection size-aware classification	Preprint	Triage and Timely Diagnosis of Infections	COVID-19	China	Detection		CNN; DT; MLP; RF; SVM	Disease		Unclear	CT	No	No
Shibly, K. H.	2020	COVID Faster R-CNN: A Novel Framework to Diagnose Novel Coronavirus Disease (COVID-19) in X-Ray Images	Preprint	Triage and Timely Diagnosis of Infections	COVID-19	Multiple countries	Detection		CNN	Disease		No	X-ray	No	Yes

Shoer, S.	2020	Who should we test for COVID-19? A triage model built from national symptom surveys	Preprint	Triage and Timely Diagnosis of Infections	COVID-19	Multiple countries	Detection		GBT	Disease		No	No	No	No
Soares, E.	2020	SARS-CoV-2 CT-scan dataset: A large dataset of real patients CT scans for SARS-CoV-2 identification	Preprint	Triage and Timely Diagnosis of Infections	COVID-19	Brazil	Detection		AdaBoost; CNN; DT; Explainable deep NN	Disease		Unclear	CT	No	No
Soares, F.	2020	A novel specific artificial intelligence-based method to identify COVID-19 cases using simple blood exams	Preprint	Triage and Timely Diagnosis of Infections	COVID-19	Brazil	Detection		k-NN; SVM	Disease		No	No	No	Yes
Song, Y.	2020	Deep learning Enables Accurate Diagnosis of Novel Coronavirus (COVID-19) with CT images	Preprint	Triage and Timely Diagnosis of Infections	COVID-19	China	Detection		CNN	Disease		Unclear	CT	No	No
Voulodimos, A.	2020	Deep learning models for COVID-19 infected area segmentation in CT images	Preprint	Triage and Timely Diagnosis of Infections	COVID-19	Multiple countries	Detection		CNN	Image segmentation		No	CT	No	Yes
Wagner, T.	2020	Augmented curation of clinical notes from a massive EHR system reveals symptoms of impending COVID-19 diagnosis	Preprint	Triage and Timely Diagnosis of Infections	COVID-19	USA	Detection		Transformer NN	Disease		Yes	No	No	No
Wang, S.	2020	A deep learning algorithm using CT images to screen for Corona Virus Disease (COVID-19)	Preprint	Triage and Timely Diagnosis of Infections	COVID-19	China	Detection		CNN	Disease		Unclear	CT	No	No
Warman, A.	2020	Interpretable Artificial Intelligence for COVID-19 Diagnosis from Chest CT Reveals Specificity of Ground-Glass Opacities	Preprint	Triage and Timely Diagnosis of Infections	COVID-19	Multiple countries	Detection		CNN; DT	Disease		No	CT	No	Yes
Wu, J.	2020	Rapid and accurate identification of COVID-19 infection through machine learning based on clinical available blood test results	Preprint	Triage and Timely Diagnosis of Infections	COVID-19	China	Detection		RF	Disease		Unclear	No	No	No
Xu, X.	2020	Deep learning system to screen coronavirus disease 2019 pneumonia	Preprint	Triage and Timely Diagnosis of Infections	COVID-19	China	Detection		CNN	Disease		Unclear	CT	No	No
Xu, Y.	2020	A collaborative online AI engine for CT-based COVID-19 diagnosis	Preprint	Triage and Timely Diagnosis of Infections	COVID-19	China	Detection		CNN	Disease		Unclear	CT	No	No
Yousefzadeh, M.	2020	ai-corona: Radiologist-Assistant Deep Learning Framework for COVID-19 Diagnosis in Chest CT Scans	Preprint	Triage and Timely Diagnosis of Infections	COVID-19	Not reported - Authors from Iran	Detection		CNN	Disease		Unclear	CT	No	No
Yu, H.	2020	Data-driven discovery of clinical routes for severity detection in COVID-19 pediatric cases	Preprint	Triage and Timely Diagnosis of Infections	COVID-19	China	Estimation		DT	Disease severity		Unclear	No	No	No
Zhang, J.	2020	Viral Pneumonia Screening on Chest X-ray Images Using Confidence-Aware Anomaly Detection	Preprint	Triage and Timely Diagnosis of Infections	COVID-19	Multiple countries	Detection		CNN; MLP	Disease		Unclear	X-ray	No	Yes
Zheng, C.	2020	Deep Learning-based Detection for COVID-19 from Chest CT using Weak Label	Preprint	Triage and Timely Diagnosis of Infections	COVID-19	China	Detection		CNN	Disease		Yes	CT	No	No

Zhou, M.	2020	Improved deep learning model for differentiating novel coronavirus pneumonia and influenza pneumonia	Preprint	Triage and Timely Diagnosis of Infections	COVID-19	Not reported - Authors from China	Detection		CNN	Disease		Unclear	CT	No	No
Zoabi, Y.	2020	COVID-19 diagnosis prediction by symptoms of tested individuals: a machine learning approach	Preprint	Triage and Timely Diagnosis of Infections	COVID-19	Israel	Detection		GBT	Disease		No	No	No	Yes
Biswas, S. K.	2014	Hybrid expert system using case based reasoning and neural network for classification	Peer-reviewed	Triage and Timely Diagnosis of Infections	H1N1	Not reported - Authors from India	Detection		k-NN; MLP	Disease		Unclear	No	No	Yes
Biswas, S. K.	2014	Intelligent decision support system of swine flu prediction using novel case classification algorithm	Peer-reviewed	Triage and Timely Diagnosis of Infections	H1N1	Not reported - Authors from India	Detection		k-NN	Disease		Unclear	No	No	Yes
Mansiaux, Y.	2014	Detection of independent associations in a large epidemiologic dataset: a comparison of random forests, boosted regression trees, conventional and penalized logistic regression for identifying independent factors associated with H1N1pdm influenza infections	Peer-reviewed	Triage and Timely Diagnosis of Infections	H1N1	France	Detection		GBT; RF	Disease		No	No	No	No
Yao, J.	2011	Computer-aided diagnosis of pulmonary infections using texture analysis and support vector machine classification	Peer-reviewed	Triage and Timely Diagnosis of Infections	H1N1	USA	Detection		SVM	Disease		No	CT	No	No
Mendis, B. S. U.	2006	Learning Generalized Weighted Relevance Aggregation Operators Using Levenberg-Marquardt Method	Conference Proceeding	Triage and Timely Diagnosis of Infections	SARS	Not reported - Authors from Australia and Hungary	Detection		Fuzzy logic	Disease		Unclear	No	No	No
Xuanyang, X.	2005	Computer Aided Detection of SARS Based on Radiographs Data Mining	Conference Proceeding	Triage and Timely Diagnosis of Infections	SARS	China	Detection		DT; MLP	Disease		Unclear	X-ray	No	No
Raghav, R. S.	2019	Bigdata fog based cyber physical system for classifying, identifying and prevention of SARS disease	Peer-reviewed	Triage and Timely Diagnosis of Infections	SARS	Not reported - Authors from India	Detection		DT; RNN	Disease		Unclear	No	No	No
Xie, X.	2006	Mining X-ray images of SARS patients	Peer-reviewed	Triage and Timely Diagnosis of Infections	SARS	Not reported - Authors from China	Detection		DT; MLP	Disease		Unclear	X-ray	No	No

Chassagnon, G.	2020	AI-Driven CT-based quantification, staging and short-term outcome prediction of COVID-19 pneumonia	Preprint	Triage and Timely Diagnosis of Infections; Prognosis of Illness and Response to Treatment	COVID-19	Blinded for peer-review	Estimation	Prediction	AdaBoost; CNN; DT; k-NN; MLP; RF; SVM	Disease severity	Death, and intubation	Unclear	CT	No	No
Elghamrawy, S. M.	2020	Diagnosis and Prediction Model for COVID-19 Patient's Response to Treatment based on Convolutional Neural Networks and Whale Optimization Algorithm Using CT Images	Preprint	Triage and Timely Diagnosis of Infections; Prognosis of Illness and Response to Treatment	COVID-19	Multiple countries	Detection	Prediction	CNN; SVM	Disease	Response to treatment	No	CT	No	Yes
Li, M. D.	2020	Automated Assessment of COVID-19 Pulmonary Disease Severity on Chest Radiographs using Convolutional Siamese Neural Networks	Preprint	Triage and Timely Diagnosis of Infections; Prognosis of Illness and Response to Treatment	COVID-19	USA	Estimation	Prediction	CNN	Disease severity	Intubation, and death	Unclear	X-ray	No	Yes
Li, X.	2020	COVID-MobileXpert: on-device covid-19 screening using snapshots of chest x-ray	Preprint	Triage and Timely Diagnosis of Infections; Prognosis of Illness and Response to Treatment	COVID-19	Multiple countries	Detection	Prediction	CNN; GBT; RF	Disease	Disease severity	No	X-ray	No	Yes
Wang, S.	2020	A Fully Automatic Deep Learning System for COVID-19 Diagnostic and Prognostic Analysis	Preprint	Triage and Timely Diagnosis of Infections; Prognosis of Illness and Response to Treatment	COVID-19	China	Detection	Prediction	CNN	Disease	Length of hospital stay	Unclear	CT	No	No
Yasar, Y.	2020	MantisCOVID: Rapid X-Ray Chest Radiograph and Mortality Rate Evaluation With Artificial Intelligence For COVID-19	Preprint	Triage and Timely Diagnosis of Infections; Prognosis of Illness and Response to Treatment	COVID-19	Multiple countries	Detection	Prediction	CNN	Disease	Death	No	X-ray	No	Yes
Wynants, L.	2020	Prediction models for diagnosis and prognosis of covid-19 infection: systematic review and critical appraisal	Peer-reviewed	Triage and Timely Diagnosis of Infections; Prognosis of Illness and Response to Treatment (Review)	COVID-19	Various - Review	Detection	Prediction	Various - Review	Various - Review	Same as outcomes for purpose 1	No	No	No	Yes

*At the time of the PubMed, Embase, Web of Science, IEEE Xplore, and ACM Guide to Computing Literature database searches (May 4, 2020).

Abbreviations: AdaBoost, adaptive boosting; AE, autoencoder; CNN, convolutional neural network; CT, computed tomography; COVID-19, coronavirus disease 2019; DT, decision tree; EHR, electronic health record; GBT, gradient boosted trees; H1N1, pandemic influenza A subtype H1N1; k-NN, k-nearest neighbors; ML, machine learning; MLP, multi-layer perceptron; NLP, natural language processing; NN, neural network (unspecified); NPI, non-pharmaceutical interventions; RF, random forest; RNN, recurrent neural network; SARS, severe acute respiratory syndrome; SLP, single layer perceptron; SVM, support vector machine.

Supplementary Table 3. Data abstraction for limited review of studies that used traditional modeling approaches

First Author Name	Release Year	Manuscript Title	Publication Status*
Aleman, D. M.	2009	Accounting for individual behaviors in a pandemic disease spread model	Conference Proceeding
Andradottir, S.	2010	Simulation of strategies for containing pandemic influenza	Conference Proceeding
Araz, O. M.	2009	A pandemic influenza simulation model for preparedness planning	Conference Proceeding
Aschwanden, C.	2004	Spatial Simulation Model for Infectious Viral Diseases with Focus on SARS and the Common Flu	Conference Proceeding
Beckman, R.	2014	ISIS: a networked-epidemiology based pervasive web app for infectious disease pandemic planning and response	Conference Proceeding
Beeler, M. F.	2012	A large simulation experiment to test influenza pandemic behavior	Conference Proceeding
Beeler, M. F.	2011	Estimation and management of pandemic influenza transmission risk at mass immunization clinics	Conference Proceeding
Bisset, K. R.	2012	High performance informatics for pandemic preparedness	Conference Proceeding
Brigantic, R.	2008	Simulation of passenger screening for pandemic influenza at U.S. airport ports of entry	Conference Proceeding
Cao, J.	2014	Evaluating the impacts of vaccination, antiviral treatment and school closure on H1N1 influenza epidemic	Conference Proceeding
Cauchemez, S.	2011	Role of social networks in shaping disease transmission during a community outbreak of 2009 H1N1 pandemic influenza	Conference Proceeding
Chang, H.-J.	2015	The Impact of Household Structures on Pandemic Influenza Vaccination Priority	Conference Proceeding
Chang, H.-J.	2014	A Comparison Between a Deterministic, Compartmental Model and an Individual Based-stochastic Model for Simulating the Transmission Dynamics of Pandemic Influenza	Conference Proceeding
Chen, Y.-D.	2007	Incorporating geographical contacts into social network analysis for contact tracing in epidemiology: a study on Taiwan SARS data	Conference Proceeding
Choi, H.	2010	A Patch Model for Pandemic Influenza Simulation in Korea	Conference Proceeding
Chu, S.	2017	A Comparison of Targeted Layered Containment Strategies for a Flu Pandemic in Three US Cities	Conference Proceeding
Dibble, C.	2010	Effective real-time allocation of pandemic interventions	Conference Proceeding
Dibble, C.	2007	Simulating pandemic influenza risks of US cities	Conference Proceeding
Duan, W.	2011	Modeling and simulation for the spread of H1N1 influenza in school using artificial societies	Conference Proceeding
Dube, M.	2019	Pandemic: A Graph Evolution Story	Conference Proceeding
Ekici, A.	2008	Pandemic influenza response	Conference Proceeding
Fang, H.	2005	Modelling the SARS epidemic by a lattice-based Monte-Carlo simulation	Conference Proceeding
Ge, Y.	2011	Agent based modeling for H1N1 influenza in artificial campus	Conference Proceeding
Goedecke, D. M.	2007	A stochastic equation-based model of the value of international air-travel restrictions for controlling pandemic flu	Conference Proceeding
Gong, J.	2006	Design and implementation of an intelligent virtual geographic environment for the simulation of SARS transmission	Conference Proceeding
Huang, C.-Y.	2011	A Multilayer Framework to Assess Influenza Intervention Policies	Conference Proceeding
Huang, Y.	2010	Modeling the severe acute respiratory syndrome (SARS) outbreak in Beijing: an agent-based approach	Conference Proceeding
Jones, D. A.	2008	Pandemic Influenza, Worker Absenteeism and Impacts on Freight Transportation	Conference Proceeding
Jumpen, W.	2011	SIS-SEIQR adaptive network model for pandemic influenza	Conference Proceeding
Lant, T.	2008	Simulating pandemic influenza preparedness plans for a public university: a hierarchical system dynamics approach	Conference Proceeding
Laobing, Z.	2013	An approach to model the interventions of unconventional emergency	Conference Proceeding
Liu, Y. L.	2010	Investigation of prediction and establishment of SIR model for H1N1 epidemic disease	Conference Proceeding
Lizon, N. E.	2010	Incorporating healthcare systems in pandemic models	Conference Proceeding
Loganathan, P.	2011	Towards forecasting flu dynamics using a regionalized state space model	Conference Proceeding
Lu, X. L.	2004	Infrastructure for Web-GIS based interoperable SARS information system	Conference Proceeding
Maki, Y.	2013	Infectious Disease Spread Analysis Using Stochastic Differential Equations for SIR Model	Conference Proceeding
Mao, L.	2013	Cost-effectiveness of workplace closure and travel restriction for mitigating influenza outbreaks: a network-based simulation	Conference Proceeding
Martin, G.	2013	Parallel algorithm for simulating the spatial transmission of influenza in EpiGraph	Conference Proceeding
Merler, S.	2006	Strategies for containing an influenza pandemic: the case of Italy	Conference Proceeding
Mhlanga, F. S.	2013	Towards a predictive model architecture for current or emergent pandemic situations	Conference Proceeding
Mniszewski, S. M.	2008	EpiSimS simulation of a multi-component strategy for pandemic influenza	Conference Proceeding
Nieto-Chaupis, H.	2019	Face To Face with Next Flu Pandemic with a Wiener-Series-Based Machine Learning: Fast Decisions to Tackle Rapid Spread	Conference Proceeding
Nuno, M.	2008	Protecting residential care facilities from pandemic influenza	Conference Proceeding
Ozaltin, O. Y.	2014	Optimal distribution of the influenza vaccine	Conference Proceeding
Paleshi, A.	2011	Simulation of mitigation strategies for a pandemic influenza	Conference Proceeding
Park, H.	2010	Extracting distinctive features of swine (H1N1) flu through data mining clinical documents	Conference Proceeding
Park, J.	2017	Epidemic Simulation of H1N1 Influenza Virus using GIS in South Korea	Conference Proceeding
Prejmerean, V.	2008	Graphical Representation of the Pandemic Spreading	Conference Proceeding

Ramos, M.	2018	E-Health:Agent-Based Models to Simulate Behavior of Individuals During an Epidemic Outbreak	Conference Proceeding
Rathore, H.	2012	Modular Network Effects on Communicable Disease Models	Conference Proceeding
Rico, F.	2007	Emergency departments nurse allocation to face a pandemic influenza outbreak	Conference Proceeding
Safarishahrbijari, A.	2015	Particle filtering in a SEIRV simulation model of H1N1 influenza	Conference Proceeding
Saito, M. M.	2011	Parallel agent-based simulator for influenza pandemic	Conference Proceeding
Saito, M. M.	2011	Estimation of macroscopic parameter in agent-based pandemic simulation	Conference Proceeding
Santos, E. E.	2011	Intent-Driven Behavioral Modeling during Cross-Border Epidemics	Conference Proceeding
Sato, H.	2016	Agent-based Infectious Diffusion Simulation using Japanese Domestic Human Mobility Data as Metapopulation Network	Conference Proceeding
See, B. D.	2009	Staffing a pandemic urgent care facility during an outbreak of pandemic influenza	Conference Proceeding
Shekh, B.	2015	Hybrid multi-threaded simulation of agent-based pandemic modeling using multiple GPUs	Conference Proceeding
Tan, W. K.	2012	Stochastic FluSiM for influenza transmission dynamics	Conference Proceeding
Thakkar, B. A.	2010	Health Care Decision Support System for Swine Flu Prediction Using Naive Bayes Classifier	Conference Proceeding
Wallace, D. I.	2014	A SIMULATION OF THE US INFLUENZA OUTBREAK IN 2009-2010 USING A PATCH SIR MODEL BASED ON AIRPORT TRANSPORTATION DATA	Conference Proceeding
Wong, Z. S.-Y.	2015	School closure strategies for the 2009 Hong Kong H1N1 influenza pandemic	Conference Proceeding
Wu, M.	2017	Optimal Fuzzy Control of SIR Epidemic with State Dependent Cost Function	Conference Proceeding
Wu, S.	2017	A Framework for Validation of Network-based Simulation Models: an Application to Modeling Interventions of Pandemics	Conference Proceeding
Xia, H.	2013	Evaluating Strategies for Pandemic Response in Delhi Using Realistic Social Networks	Conference Proceeding
Yarmand, H.	2010	Cost-effectiveness analysis of vaccination and self-isolation in case of H1N1	Conference Proceeding
Zhang, Y.	2010	Spatially Explicit Epidemiological Simulation System of Influenza A (H1N1) in China	Conference Proceeding
Zheng, X.	2008	Network-Based Analysis of Beijing SARS Data	Conference Proceeding
Zhou, J.	2006	Human Daily Behavior Based Simulation for Epidemic Transmission: A Case Study of SARS	Conference Proceeding
Adegboye, O. A.	2020	Early Transmission Dynamics of Novel Coronavirus (COVID-19) in Nigeria	Peer-reviewed
Aleman, D. M.	2011	A Nonhomogeneous Agent-Based Simulation Approach to Modeling the Spread of Disease in a Pandemic Outbreak	Peer-reviewed
Ali, S. T.	2013	Transmission dynamics of the 2009 influenza A (H1N1) pandemic in India: the impact of holiday-related school closure	Peer-reviewed
An der Heiden, M.	2009	Breaking the waves: modelling the potential impact of public health measures to defer the epidemic peak of novel influenza A/H1N1	Peer-reviewed
Anastassopoulou, C.	2020	Data-based analysis, modelling and forecasting of the COVID-19 outbreak	Peer-reviewed
Araz, O. M.	2013	Simulation modeling for pandemic decision making: A case study with bi-criteria analysis on school closures	Peer-reviewed
Arora, H.	2012	Decision support for containing pandemic propagation	Peer-reviewed
Azmon, A.	2014	On the estimation of the reproduction number based on misreported epidemic data	Peer-reviewed
Barrett, C.	2010	An Integrated Modeling Environment to Study the Coevolution of Networks, Individual Behavior, and Epidemics	Peer-reviewed
Bastos, L. S.	2019	A modelling approach for correcting reporting delays in disease surveillance data	Peer-reviewed
Bekiros, S.	2020	SBDiEM: A new Mathematical model of Infectious Disease Dynamics	Peer-reviewed
Bilge, A. H.	2018	Determination of epidemic parameters from early phase fatality data: A case study of the 2009 A(H1N1) pandemic in Europe	Peer-reviewed
Bilge, A. H.	2015	On the uniqueness of epidemic models fitting a normalized curve of removed individuals	Peer-reviewed
Bin, S.	2019	Spread of infectious disease modeling and analysis of different factors on spread of infectious disease based on cellular automata	Peer-reviewed
Birrell, P. J.	2017	Real-time modelling of a pandemic influenza outbreak	Peer-reviewed
Birrell, P. J.	2011	Bayesian modeling to unmask and predict influenza A/H1N1pdm dynamics in London	Peer-reviewed
Bouaine, A.	2019	Indirect Optimal Approach Applied to H1N1 Spread Through Moroccan Regions	Peer-reviewed
Buonomo, B.	2020	Effects of information-dependent vaccination behavior on coronavirus outbreak: insights from a SIRI model	Peer-reviewed
Burger, R.	2016	Modelling the spatial-temporal progression of the 2009 A/H1N1 influenza pandemic in Chile	Peer-reviewed
Burr, T. L.	2008	Signatures of non-homogeneous mixing in disease outbreaks	Peer-reviewed
Campbell, F.	2019	Bayesian inference of transmission chains using timing of symptoms, pathogen genomes and contact data	Peer-reviewed
Cao, C.	2016	Analysis of Spatiotemporal Characteristics of Pandemic SARS Spread in Mainland China	Peer-reviewed
Cao, Z.	2010	Spatio-temporal evolution of Beijing 2003 SARS epidemic	Peer-reviewed
Cauchemez, S.	2014	Determinants of influenza transmission in South East Asia: insights from a household cohort study in Vietnam	Peer-reviewed
Cauchemez, S.	2006	Estimating in real time the efficacy of measures to control emerging communicable diseases	Peer-reviewed
Cauchemez, S.	2006	Real-time estimates in early detection of SARS	Peer-reviewed
Chan, J. S. K.	2006	Modelling SARS data using threshold geometric process	Peer-reviewed
Chang, C.	2010	The novel H1N1 Influenza A global airline transmission and early warning without travel containments	Peer-reviewed
Chang, R.	2020	Phase- and epidemic region-adjusted estimation of the number of coronavirus disease 2019 cases in China	Peer-reviewed
Chatterjee, K.	2020	Healthcare impact of COVID-19 epidemic in India: A stochastic mathematical model	Peer-reviewed
Chen, S. C.	2008	Modelling control measures to reduce the impact of pandemic influenza among schoolchildren	Peer-reviewed
Chen, T.	2017	Evaluating the effects of common control measures for influenza A (H1N1) outbreak at school in China: A modeling study	Peer-reviewed

Cheng, Z. L.	2012	An Agent-Based Artificial Transportation System Framework for H1N1 Transmission Simulation	Peer-reviewed
Chong, K. C	2018	Approximate Bayesian algorithm to estimate the basic reproduction number in an influenza pandemic using arrival times of imported cases	Peer-reviewed
Chong, K. C.	2017	A statistical method utilizing information of imported cases to estimate the transmissibility for an influenza pandemic	Peer-reviewed
Chong, K. C.	2014	Estimating the incidence reporting rates of new influenza pandemics at an early stage using travel data from the source country	Peer-reviewed
Chong, K. C.	2012	Modeling the impact of air, sea, and land travel restrictions supplemented by other interventions on the emergence of a new influenza pandemic virus	Peer-reviewed
Chowell, G.	2007	Comparative estimation of the reproduction number for pandemic influenza from daily case notification data	Peer-reviewed
Ciofi degli Atti, M. L.	2008	Mitigation measures for pandemic influenza in Italy: an individual based model considering different scenarios	Peer-reviewed
Clamer, V.	2016	Estimating transmission probability in schools for the 2009 H1N1 influenza pandemic in Italy	Peer-reviewed
Cooley, P.	2010	Protecting health care workers: a pandemic simulation based on Allegheny County	Peer-reviewed
Cori, A.	2012	Estimating influenza latency and infectious period durations using viral excretion data	Peer-reviewed
Cori, A.	2009	Temporal variability and social heterogeneity in disease transmission: the case of SARS in Hong Kong	Peer-reviewed
Cruz-Aponte, M.	2016	Metapopulation and Non-proportional Vaccination Models Overview	Peer-reviewed
Cruz-Aponte, M.	2011	Mitigating effects of vaccination on influenza outbreaks given constraints in stockpile size and daily administration capacity	Peer-reviewed
Cui, J.	2019	Influence of asymptomatic infections for the effectiveness of facemasks during pandemic influenza	Peer-reviewed
da Costa, A. C. C.	2018	Spatiotemporal diffusion of influenza A (H1N1): Starting point and risk factors	Peer-reviewed
de Silva, E.	2012	Inferring pandemic growth rates from sequence data	Peer-reviewed
Denphedtnong, A.	2013	On the dynamics of SEIRS epidemic model with transport-related infection	Peer-reviewed
Distante, C.	2020	Covid-19 Outbreak Progression in Italian Regions: Approaching the Peak by the End of March in Northern Italy and First Week of April in Southern Italy	Peer-reviewed
Donnelly, C. A.	2003	Epidemiological determinants of spread of causal agent of severe acute respiratory syndrome in Hong Kong	Peer-reviewed
Dorigatti, I	2012	A new approach to characterising infectious disease transmission dynamics from sentinel surveillance: Application to the Italian 2009-2010 A/H1N1 influenza pandemic	Peer-reviewed
Dukic, V.	2012	Tracking Epidemics With Google Flu Trends Data and a State-Space SEIR Model	Peer-reviewed
Dureau, J.	2013	Capturing the time-varying drivers of an epidemic using stochastic dynamical systems	Peer-reviewed
Earnest, A.	2005	Using autoregressive integrated moving average (ARIMA) models to predict and monitor the number of beds occupied during a SARS outbreak in a tertiary hospital in Singapore	Peer-reviewed
Eggo, R. M.	2011	Spatial dynamics of the 1918 influenza pandemic in England, Wales and the United States	Peer-reviewed
Eichner, M.	2007	The influenza pandemic preparedness planning tool InfluSim	Peer-reviewed
Ekici, A.	2014	Modeling Influenza Pandemic and Planning Food Distribution	Peer-reviewed
Fang, H. P.	2004	On the origin of the super-spreading events in the SARS epidemic	Peer-reviewed
Fang, Y.	2020	Transmission dynamics of the COVID-19 outbreak and effectiveness of government interventions: A data-driven analysis	Peer-reviewed
Farah, M.	2014	Bayesian Emulation and Calibration of a Dynamic Epidemic Model for A/H1N1 Influenza	Peer-reviewed
Fatima-Zohra, Y.	2015	A Surveillance and Spatiotemporal Visualization Model for Infectious Diseases using Social Network	Peer-reviewed
Feng, Z.	2017	Evaluating targeted interventions via meta-population models with multi-level mixing	Peer-reviewed
Feng, Z.	2011	Modeling the effects of vaccination and treatment on pandemic influenza	Peer-reviewed
Fierro, A.	2013	Lattice model for influenza spreading with spontaneous behavioral changes	Peer-reviewed
Fierro, A.	2011	A simple stochastic lattice gas model for H1N1 pandemic. Application to the Italian epidemiological data	Peer-reviewed
Finkelstein, S. N.	2015	Engineering Effective Responses to Influenza Outbreaks	Peer-reviewed
Flahault, A.	2009	Potential for a global dynamic of Influenza A (H1N1)	Peer-reviewed
Flahault, A.	2006	Strategies for containing a global influenza pandemic	Peer-reviewed
Freiesleben de Blasio, B.	2012	Effect of vaccines and antivirals during the major 2009 a(H1N1) pandemic wave in norway - and the influence of vaccination timing	Peer-reviewed
Fujie, R.	2007	Effects of superspreaders in spread of epidemic	Peer-reviewed
Fukutome, A.	2007	Mathematical modeling of severe acute respiratory syndrome nosocomial transmission in Japan: the dynamics of incident cases and prevalent cases	Peer-reviewed
Furushima, D.	2017	Estimation of the Basic Reproduction Number of Novel Influenza A (H1N1) pdm09 in Elementary Schools Using the SIR Model	Peer-reviewed
Gani, S. R.	2011	The transmission dynamics of pandemic influenza A/H1N1 2009-2010 in India	Peer-reviewed
Gao, X. L.	2009	Ventilation Control of Indoor Transmission of Airborne Diseases in an Urban Community	Peer-reviewed
Gao, Z. M.	2012	Epidemic Spreading in a Multi-compartment System	Peer-reviewed
Garcia, Y. E.	2015	A Bayesian Outbreak Detection Method for Influenza-Like Illness	Peer-reviewed
Gatto, M.	2020	Spread and dynamics of the COVID-19 epidemic in Italy: Effects of emergency containment measures	Peer-reviewed
Glass, K.	2007	Predicting case numbers during infectious disease outbreaks when some cases are undiagnosed	Peer-reviewed
Gomez-Barroso, D.	2014	Geographical spread of influenza incidence in Spain during the 2009 A(H1N1) pandemic wave and the two succeeding influenza seasons	Peer-reviewed
Gonzalez-Parra, G.	2015	Modelling influenza A(H1N1) 2009 epidemics using a random network in a distributed computing environment	Peer-reviewed
Gonzalez-Parra, G.	2011	Modeling the epidemic waves of AH1N1/09 influenza around the world	Peer-reviewed
Goudie, R. J. B.	2019	Joining and splitting models with Markov melding	Peer-reviewed
Grais, R. F.	2003	Assessing the impact of airline travel on the geographic spread of pandemic influenza	Peer-reviewed
Heaton, M. J.	2012	A spatio-temporal absorbing state model for disease and syndromic surveillance	Peer-reviewed

Hill, E. M.	2017	Evidence for history-dependence of influenza pandemic emergence	Peer-reviewed
Hirose, H.	2007	The mixed trunsored model with applications to SARS	Peer-reviewed
Hou, C.	2020	The effectiveness of quarantine of Wuhan city against the Corona Virus Disease 2019 (COVID-19): A well-mixed SEIR model analysis	Peer-reviewed
House, T.	2012	Estimation of outbreak severity and transmissibility: Influenza A(H1N1)pdm09 in households	Peer-reviewed
Hu, H.	2015	Information Dissemination of Public Health Emergency on Social Networks and Intelligent Computation	Peer-reviewed
Hu, W.	2015	Socio-Ecological Drivers and 2009 Pandemic Influenza A (H1N1): A Bayesian Dynamic Spatiotemporal Model	Peer-reviewed
Hu, W.	2012	Did socio-ecological factors drive the spatiotemporal patterns of pandemic influenza A (H1N1)?	Peer-reviewed
Huang, R.	2020	Spatial-temporal distribution of COVID-19 in China and its prediction: A data-driven modeling analysis	Peer-reviewed
Huang, X.	2016	Bayesian estimation of the dynamics of pandemic (H1N1) 2009 influenza transmission in Queensland: A space-time SIR-based model	Peer-reviewed
Hwang, G. M.	2012	A model-based tool to predict the propagation of infectious disease via airports	Peer-reviewed
Ivorra, B.	2020	Mathematical modeling of the spread of the coronavirus disease 2019 (COVID-19) taking into account the undetected infections. The case of China	Peer-reviewed
Iwata, K.	2020	A Simulation on Potential Secondary Spread of Novel Coronavirus in an Exported Country Using a Stochastic Epidemic SEIR Model	Peer-reviewed
Izadi, M.	2017	Using Dynamic Bayesian Networks for Incorporating Nontraditional Data Sources in Public Health Surveillance	Peer-reviewed
Jiang, C.	2007	Optimal control of SARS epidemics based on cybernetics	Peer-reviewed
Jin, Z.	2007	A cellular automata model with probability infection and spatial dispersion	Peer-reviewed
Jombart, T.	2014	Bayesian reconstruction of disease outbreaks by combining epidemiologic and genomic data	Peer-reviewed
Kadi, A. S.	2015	A Bayesian inferential approach to quantify the transmission intensity of disease outbreak	Peer-reviewed
Karako, K.	2020	Analysis of COVID-19 infection spread in Japan based on stochastic transition model	Peer-reviewed
Kass-Hout, T. A.	2012	Application of change point analysis to daily influenza-like illness emergency department visits	Peer-reviewed
Katriel, G.	2011	Modelling the initial phase of an epidemic using incidence and infection network data: 2009 H1N1 pandemic in Israel as a case study	Peer-reviewed
Katriel, G.	2010	Pandemic dynamics and the breakdown of herd immunity	Peer-reviewed
Kelly, H. A.	2010	Pandemic (H1N1) 2009 influenza community transmission was established in one Australian state when the virus was first identified in North America	Peer-reviewed
Kenah, E.	2013	Non-parametric survival analysis of infectious disease data	Peer-reviewed
Ketsetzis, G.	2010	REAL-TIME FORECASTING FOR AN INFLUENZA PANDEMIC IN THE UK FROM PRIOR INFORMATION AND MULTIPLE SURVEILLANCE DATASETS	Peer-reviewed
Kim, S.	2020	School Opening Delay Effect on Transmission Dynamics of Coronavirus Disease 2019 in Korea: Based on Mathematical Modeling and Simulation Study	Peer-reviewed
Kock, K. D.	2017	Calculation of reproducibility rates (R-0) by simplification of SIR model applied to Influenza A epidemic (H1N1) in Brazil occurred in 2009	Peer-reviewed
Kostkova, P.	2014	#swineflu: The Use of Twitter as an Early Warning and Risk Communication Tool in the 2009 Swine Flu Pandemic	Peer-reviewed
Koyuncu, M.	2010	Optimal Resource Allocation Model to Mitigate the Impact of Pandemic Influenza: A Case Study for Turkey	Peer-reviewed
Kumar, V.	2016	SIR Model of Swine Flu in Shimla	Peer-reviewed
Kuniya, T.	2020	Prediction of the Epidemic Peak of Coronavirus Disease in Japan, 2020	Peer-reviewed
Kwok, K. O.	2020	Epidemiological characteristics of the first 53 laboratory-confirmed cases of COVID-19 epidemic in Hong Kong, 13 February 2020	Peer-reviewed
Kwok, K. O.	2019	Epidemic Models of Contact Tracing: Systematic Review of Transmission Studies of Severe Acute Respiratory Syndrome and Middle East Respiratory Syndrome	Peer-reviewed
Laguzet, L.	2015	Individual Vaccination as Nash Equilibrium in a SIR Model with Application to the 2009-2010 Influenza A (H1N1) Epidemic in France	Peer-reviewed
Lai, D.	2012	Controlling influenza A (H1N1) in China: Bayesian or frequentist approach	Peer-reviewed
Lai, P.-C.	2015	An early warning system for detecting H1N1 disease outbreak - a spatio-temporal approach	Peer-reviewed
Laskowski, M.	2011	Agent-Based Modeling of the Spread of Influenza-Like Illness in an Emergency Department: A Simulation Study	Peer-reviewed
Lechien, J. R.	2020	Clinical and Epidemiological Characteristics of 1,420 European Patients with mild-to-moderate Coronavirus Disease 2019	Peer-reviewed
Lee, E. C.	2018	Deploying digital health data to optimize influenza surveillance at national and local scales	Peer-reviewed
Lee, E. K.	2015	Vaccine Prioritization for Effective Pandemic Response	Peer-reviewed
Lee, H.	2016	Stochastic methods for epidemic models: An application to the 2009 H1N1 influenza outbreak in Korea	Peer-reviewed
Lee, J. M.	2012	The effect of public health interventions on the spread of influenza among cities	Peer-reviewed
Lee, V. J.	2011	Comparability of different methods for estimating influenza infection rates over a single epidemic wave	Peer-reviewed
Lekone, P. E.	2008	Bayesian analysis of severe acute respiratory syndrome: the 2003 Hong Kong epidemic	Peer-reviewed
Leung, K.	2020	First-wave COVID-19 transmissibility and severity in China outside Hubei after control measures, and second-wave scenario planning: a modelling impact assessment	Peer-reviewed
Li, B. Z.	2020	Strong policies control the spread of COVID-19 in China	Peer-reviewed
Li, R.	2020	Substantial undocumented infection facilitates the rapid dissemination of novel coronavirus (SARS-CoV-2)	Peer-reviewed
Li, S.	2020	Preliminary Assessment of the COVID-19 Outbreak Using 3-Staged Model e-ISHR	Peer-reviewed
Li, W.	2011	Effects of Variant Rates and Noise on Epidemic Spreading	Peer-reviewed
Li, X.	2013	Was mandatory quarantine necessary in China for controlling the 2009 H1N1 pandemic?	Peer-reviewed
Liao, C. M.	2005	A probabilistic transmission dynamic model to assess indoor airborne infection risks	Peer-reviewed
Liccardo, A.	2013	A lattice model for influenza spreading	Peer-reviewed
Ling, M. H.	2017	Efficient heterogeneous sampling for stochastic simulation with an illustration in health care applications	Peer-reviewed
Liu, C.	2020	D(2)EA: Depict the Epidemic Picture of COVID-19	Peer-reviewed

Liu, M.	2015	A dynamic allocation model for medical resources in the control of influenza diffusion	Peer-reviewed
Lopez, D.	2017	Modelling the H1N1 influenza using mathematical and neural network approaches	Peer-reviewed
Lunelli, A.	2009	Epidemic patch models applied to pandemic influenza: contact matrix, stochasticity, robustness of predictions	Peer-reviewed
Ma, J.	2013	The importance of contact network topology for the success of vaccination strategies	Peer-reviewed
Maciejewski, R.	2011	A pandemic influenza modeling and visualization tool	Peer-reviewed
Maeno, Y.	2016	Detecting a trend change in cross-border epidemic transmission	Peer-reviewed
Magal, P.	2018	Final size of a multi-group SIR epidemic model: Irreducible and non-irreducible modes of transmission	Peer-reviewed
Malik, R.	2014	Individual-level modeling of the spread of influenza within households	Peer-reviewed
Marcelino, J.	2012	Critical paths in a metapopulation model of H1N1: Efficiently delaying influenza spreading through flight cancellation	Peer-reviewed
Marmara, V.	2014	Estimation of force of infection based on different epidemiological proxies: 2009/2010 Influenza epidemic in Malta	Peer-reviewed
Martinez-Beneito, M. A.	2011	A kernel-based spatio-temporal surveillance system for monitoring influenza-like illness incidence	Peer-reviewed
Marziano, V.	2017	Detecting a Surprisingly Low Transmission Distance in the Early Phase of the 2009 Influenza Pandemic	Peer-reviewed
McBryde, E. S.	2006	Bayesian modelling of an epidemic of severe acute respiratory syndrome	Peer-reviewed
Mills, C. E.	2004	Transmissibility of 1918 pandemic influenza	Peer-reviewed
Mizumoto, K.	2013	Effectiveness of antiviral prophylaxis coupled with contact tracing in reducing the transmission of the influenza A (H1N1-2009): a systematic review	Peer-reviewed
Mniszewski, S.M.	2008	Pandemic simulation of antivirals + school closures: buying time until strain-specific vaccine is available	Peer-reviewed
Modchang, C.	2012	A modeling study of school closure to reduce influenza transmission: A case study of an influenza A (H1N1) outbreak in a private Thai school	Peer-reviewed
Morales, K. F.	2017	Possible explanations for why some countries were harder hit by the pandemic influenza virus in 2009 - a global mortality impact modeling study	Peer-reviewed
Moser, C. B.	2015	The impact of prior information on estimates of disease transmissibility using Bayesian tools	Peer-reviewed
Moss, R.	2011	Diagnosis and antiviral intervention strategies for mitigating an influenza epidemic	Peer-reviewed
Naheed, A.	2014	Numerical study of SARS epidemic model with the inclusion of diffusion in the system	Peer-reviewed
Ng, T. W.	2003	A double epidemic model for the SARS propagation	Peer-reviewed
Niehus, R.	2020	Using observational data to quantify bias of traveller-derived COVID-19 prevalence estimates in Wuhan, China	Peer-reviewed
Ohkusa, Y.	2011	Real-time estimation and prediction for pandemic A/H1N1(2009) in Japan	Peer-reviewed
Patel, R.	2005	Finding optimal vaccination strategies for pandemic influenza using genetic algorithms	Peer-reviewed
Peirlinck, M.	2020	Outbreak dynamics of COVID-19 in China and the United States	Peer-reviewed
Popinga, A.	2015	Inferring epidemiological dynamics with Bayesian coalescent inference: the merits of deterministic and stochastic models	Peer-reviewed
Prem, K.	2020	The effect of control strategies to reduce social mixing on outcomes of the COVID-19 epidemic in Wuhan, China: a modelling study	Peer-reviewed
Presanis, A. M.	2014	SYNTHESISING EVIDENCE TO ESTIMATE PANDEMIC (2009) A/H1N1 INFLUENZA SEVERITY IN 2009-2011	Peer-reviewed
Presanis, A. M.	2011	Changes in severity of 2009 pandemic A/H1N1 influenza in England: a Bayesian evidence synthesis	Peer-reviewed
Presanis, A. M.	2009	The severity of pandemic H1N1 influenza in the United States, from April to July 2009: a Bayesian analysis	Peer-reviewed
Presanis, A. M.	2009	The severity of pandemic H1N1 influenza in the United States, April - July 2009	Peer-reviewed
Que, J.	2012	Spatial and Temporal Algorithm Evaluation for Detecting Over-The-Counter Thermometer Sale Increases during 2009 H1N1 Pandemic	Peer-reviewed
Rabiei Motlagh, O.	2011	An SIR estimation for pandemic by influenza A during the haj	Peer-reviewed
Rakowski, F.	2010	Influenza epidemic spread simulation for Poland - a large scale, individual based model study	Peer-reviewed
Riley, P.	2013	Multiple estimates of transmissibility for the 2009 influenza pandemic based on influenza-like-illness data from small US military populations	Peer-reviewed
Rizzo, C.	2008	Scenarios of diffusion and control of an influenza pandemic in Italy	Peer-reviewed
Roberts, M. G.	2013	Epidemic models with uncertainty in the reproduction number	Peer-reviewed
Roberts, M. G.	2004	Modelling strategies for minimizing the impact of an imported exotic infection	Peer-reviewed
Rocklov, J.	2020	COVID-19 outbreak on the Diamond Princess cruise ship: estimating the epidemic potential and effectiveness of public health countermeasures	Peer-reviewed
Roda, W. C.	2020	Why is it difficult to accurately predict the COVID-19 epidemic?	Peer-reviewed
Roosa, K.	2019	Assessing parameter identifiability in compartmental dynamic models using a computational approach: application to infectious disease transmission models	Peer-reviewed
Safarishahrbijari, A.	2019	Social Media Surveillance for Outbreak Projection via Transmission Models: Longitudinal Observational Study	Peer-reviewed
Saito, M. M.	2013	Extension and verification of the SEIR model on the 2009 influenza A (H1N1) pandemic in Japan	Peer-reviewed
Samsuzzoha, M.	2013	Parameter estimation of influenza epidemic model	Peer-reviewed
Santos, E. E.	2013	Modeling Emergent Border-Crossing Behaviors during Pandemics	Peer-reviewed
Schimit, P. H. T.	2010	Who should wear mask against airborne infections? Altering the contact network for controlling the spread of contagious diseases	Peer-reviewed
Schwartz, E. J.	2015	Estimating epidemic parameters: Application to H1N1 pandemic data	Peer-reviewed
Sertsou, G.	2006	Key transmission parameters of an institutional outbreak during the 1918 influenza pandemic estimated by mathematical modelling	Peer-reviewed
Shao, Q.	2015	Influences on influenza transmission within terminal based on hierarchical structure of personal contact network	Peer-reviewed
Shen, Y.	2012	Multivariate Bayesian modeling of known and unknown causes of events-An application to biosurveillance	Peer-reviewed
Shen, Y.	2009	Bayesian modeling of unknown diseases for biosurveillance	Peer-reviewed
Shubin, M.	2014	Estimating the burden of A(H1N1)pdm09 influenza in Finland during two seasons	Peer-reviewed

Silva, J. C.	2013	Comparing the accuracy of syndrome surveillance systems in detecting influenza-like illness: GUARDIAN vs. RODS vs. electronic medical record reports	Peer-reviewed
Smirnova, A.	2019	Forecasting Epidemics Through Nonparametric Estimation of Time-Dependent Transmission Rates Using the SEIR Model	Peer-reviewed
Springborn, M.	2015	Accounting for behavioral responses during a flu epidemic using home television viewing	Peer-reviewed
Sun, L.	2014	Multi-objective optimization models for patient allocation during a pandemic influenza outbreak	Peer-reviewed
Tan, X.	2013	Modeling the initial transmission dynamics of influenza A H1N1 in Guangdong Province, China	Peer-reviewed
Tang, B.	2020	Estimation of the Transmission Risk of the 2019-nCoV and Its Implication for Public Health Interventions	Peer-reviewed
Tang, B.	2020	The effectiveness of quarantine and isolation determine the trend of the COVID-19 epidemics in the final phase of the current outbreak in China	Peer-reviewed
te Beest, D. E.	2013	Estimating the generation interval of influenza A (H1N1) in a range of social settings	Peer-reviewed
Teytelman, A.	2013	Multiregional Dynamic Vaccine Allocation During an Influenza Epidemic	Peer-reviewed
Thommes, E. W.	2016	Absenteeism Impact on Local Economy During a Pandemic via Hybrid SIR Dynamics	Peer-reviewed
Tiwari, S.	2020	Outbreak trends of CoronaVirus (COVID-19) in India: A Prediction	Peer-reviewed
Tomar, A.	2020	Prediction for the spread of COVID-19 in India and effectiveness of preventive measures	Peer-reviewed
Towers, S.	2012	The impact of school closures on pandemic influenza: Assessing potential repercussions using a seasonal SIR model	Peer-reviewed
Towers, S.	2011	Antiviral treatment for pandemic influenza: assessing potential repercussions using a seasonally forced SIR model	Peer-reviewed
Towers, S.	2009	Pandemic H1N1 influenza: predicting the course of a pandemic and assessing the efficacy of the planned vaccination programme in the United States	Peer-reviewed
Tsai, M.-T.	2010	Efficient simulation of the spatial transmission dynamics of influenza	Peer-reviewed
Tsai, Y.-S.	2011	Integrating epidemic dynamics with daily commuting networks: building a multilayer framework to assess influenza A (H1N1) intervention policies	Peer-reviewed
Tsang, T. K.	2016	Interpreting Seroepidemiologic Studies of Influenza in a Context of Nonbracketing Sera	Peer-reviewed
Tsui, F.	2011	Probabilistic case detection for disease surveillance using data in electronic medical records	Peer-reviewed
Tuli, S.	2020	Predicting the Growth and Trend of COVID-19 Pandemic using Machine Learning and Cloud Computing	Peer-reviewed
Uribe-Sánchez, A.	2011	A predictive decision-aid methodology for dynamic mitigation of influenza pandemics	Peer-reviewed
Vaidya, N. K.	2015	Modelling the epidemic spread of an H1N1 influenza outbreak in a rural university town	Peer-reviewed
Volpert, V.	2020	On a quarantine model of coronavirus infection and data analysis	Peer-reviewed
Wan, K.	2020	When will the battle against novel coronavirus end in Wuhan: A SEIR modeling analysis	Peer-reviewed
Wan, X.	2014	Inferring Epidemic Network Topology from Surveillance Data	Peer-reviewed
Wang, H.	2020	Phase-adjusted estimation of the number of Coronavirus Disease 2019 cases in Wuhan, China	Peer-reviewed
Wang, J. B.	2019	Uncovering Spatial Invasion on Metapopulation Networks with SIR Epidemics	Peer-reviewed
Wang, X. S.	2012	Richards model revisited: validation by and application to infection dynamics	Peer-reviewed
Wang, Y.	2015	Revisiting node-based SIR models in complex networks with degree correlations	Peer-reviewed
Weissman, G. E.	2020	Locally Informed Simulation to Predict Hospital Capacity Needs During the COVID-19 Pandemic	Peer-reviewed
Weng, W.	2015	Evaluation of containment and mitigation strategies for an influenza A pandemic in China	Peer-reviewed
Wilson, S.	2019	Green Simulation of Pandemic Disease Propagation	Peer-reviewed
Wu, J. T.	2020	Nowcasting and forecasting the potential domestic and international spread of the 2019-nCoV outbreak originating in Wuhan, China: a modelling study	Peer-reviewed
Xia, H.	2015	Synthesis of a high resolution social contact network for Delhi with application to pandemic planning	Peer-reviewed
Xiao, H.	2013	Influence of extreme weather and meteorological anomalies on outbreaks of influenza A (H1N1)	Peer-reviewed
Xue, Y.	2012	Dynamic modelling of costs and health consequences of school closure during an influenza pandemic	Peer-reviewed
Yan, Q.	2016	Media coverage and hospital notifications: Correlation analysis and optimal media impact duration to manage a pandemic	Peer-reviewed
Yan, Q. L.	2018	Impact of individual behaviour change on the spread of emerging infectious diseases	Peer-reviewed
Yan, X.	2008	Optimal and sub-optimal quarantine and isolation control in SARS epidemics	Peer-reviewed
Yang, F.	2013	Bayesian estimation of the effective reproduction number for pandemic influenza A H1N1 in Guangdong Province, China	Peer-reviewed
Yang, K. H.	2015	Application of a modified SIR-based model-case of H1N1 in Taiwan	Peer-reviewed
Yang, W.	2015	Inference of seasonal and pandemic influenza transmission dynamics	Peer-reviewed
Yang, W.	2015	Forecasting Influenza Epidemics in Hong Kong	Peer-reviewed
Yang, Y.	2009	A Bayesian model for evaluating influenza antiviral efficacy in household studies with asymptomatic infections	Peer-reviewed
Yang, Z.	2020	Modified SEIR and AI prediction of the epidemics trend of COVID-19 in China under public health interventions	Peer-reviewed
Yoneyama, T.	2012	Simulating the spread of influenza pandemic of 2009 considering international traffic	Peer-reviewed
Yoneyama, T.	2012	A Hybrid Model for Disease Spread and an Application to the SARS Pandemic	Peer-reviewed
You, S. H.	2013	Linking contact behavior and droplet patterns to dynamically model indoor respiratory infections among schoolchildren	Peer-reviewed
Yu, X.	2013	Change-Point Detection in Binomial Thinning Processes, with Applications in Epidemiology	Peer-reviewed
Yu, Z.	2016	Efficient Vaccine Distribution Based on a Hybrid Compartmental Model	Peer-reviewed
Yu, Z.	2015	Inferring a district-based hierarchical structure of social contacts from census data	Peer-reviewed
Yue, M.	2020	Estimating the Size of a COVID-19 Epidemic from Surveillance Systems	Peer-reviewed
Zareie, B.	2020	A Model for COVID-19 Prediction in Iran Based on China Parameters	Peer-reviewed

Zhang, J.	2020	Evolving epidemiology and transmission dynamics of coronavirus disease 2019 outside Hubei province, China: a descriptive and modelling study	Peer-reviewed
Zhang, J.	2005	A compartmental model for the analysis of SARS transmission patterns and outbreak control measures in China	Peer-reviewed
Zhang, M. X.	2020	Quarantine vehicle scheduling for transferring high-risk individuals in epidemic areas	Peer-reviewed
Zhang, Y.	2014	Characterizing Influenza surveillance systems performance: application of a Bayesian hierarchical statistical model to Hong Kong surveillance data	Peer-reviewed
Zhang, Z.	2007	The outbreak pattern of SARS cases in China as revealed by a mathematical model	Peer-reviewed
Zhong, W.	2017	Simulating influenza pandemic dynamics with public risk communication and individual responsive behavior	Peer-reviewed
Zhong, W.	2013	Modeling dynamics of an influenza pandemic with heterogeneous coping behaviors: case study of a 2009 H1N1 outbreak in Arizona	Peer-reviewed
Zhou, T.	2020	Preliminary prediction of the basic reproduction number of the Wuhan novel coronavirus 2019-nCoV	Peer-reviewed
Zhou, X.	2018	A Spatial-Temporal Method to Detect Global Influenza Epidemics Using Heterogeneous Data Collected from the Internet	Peer-reviewed
Zhou, Y.	2004	A discrete epidemic model for SARS transmission and control in China	Peer-reviewed
Zou, J.	2018	A hybrid hierarchical Bayesian model for spatiotemporal surveillance data	Peer-reviewed
Aadhityaa, M.	2020	A Global Scale Estimate of Novel Coronavirus (COVID-19) Cases Using Extreme Value Distributions	Preprint
Abdeljaoued-Tej, I.	2020	COVID-19 data analysis and modeling in Palestine	Preprint
Abdeljaoued-Tej, I.	2020	A pandemic at the Tunisian scale. Mathematical modelling of reported and unreported COVID-19 infected cases	Preprint
Abdollahi, A.	2020	Effect of Temperature on the Transmission of COVID-19: A Machine Learning Case Study in Spain	Preprint
Abdulrahman, I.	2020	SimCOVID: An Open-Source Simulink-Based Program for Simulating the COVID-19 Epidemic	Preprint
Abhari, R. S.	2020	COVID-19 Epidemic in Switzerland: Growth Prediction and Containment Strategy Using Artificial Intelligence and Big Data	Preprint
Abhijit Dandekar, R.	2020	Safe Blues: A Method for Estimation and Control in the Fight Against COVID-19	Preprint
Aboelkassem, Y.	2020	COVID-19 pandemic: A Hill type mathematical model predicts the US death number and the reopening date	Preprint
Acuna-Zegarra, M. A.	2020	The SARS-CoV-2 epidemic outbreak: a review of plausible scenarios of containment and mitigation for Mexico	Preprint
Adegboye, O.	2020	Novel Coronavirus in Nigeria: Epidemiological analysis of the first 45 days of the pandemic	Preprint
Adegboye, O.	2020	Change in outbreak epicenter and its impact on the importation risks of COVID-19 progression: a modelling study	Preprint
Adekunle, A. I.	2020	Is Nigeria really on top of COVID-19? Message from effective reproduction number	Preprint
Adekunle, A. I.	2020	Delaying the COVID-19 epidemic in Australia: Evaluating the effectiveness of international travel bans	Preprint
Adwibowo, A.	2020	Flattening the COVID 19 curve in susceptible forest indigenous tribes using SIR model	Preprint
Adwibowo, A.	2020	Forecasting undetected COVID-19 cases in Small Island Developing States using Bayesian approach	Preprint
Aggarwal, N.	2020	Importance of Social Distancing: Modeling the spread of 2019-nCoV using Susceptible-Infected-Quarantined-Recovered-t model	Preprint
Aguiar, M.	2020	Modeling COVID 19 in the Basque Country: from introduction to control measure response	Preprint
Aguilar, J. B.	2020	Investigating the Impact of Asymptomatic Carriers on COVID-19 Transmission	Preprint
Ahmadi, A.	2020	Modeling and Forecasting Trend of COVID-19 Epidemic in Iran	Preprint
Ai, L.	2020	Modelling the epidemic trend of the 2019-nCOV outbreak in Hubei Province, China	Preprint
Akamatsu, T.	2020	Can a herd immunity strategy become a viable option against COVID-19? A model-based analysis on social acceptability and feasibility	Preprint
Akay, H.	2020	MARKOVIAN RANDOM WALK MODELING AND VISUALIZATION OF THE EPIDEMIC SPREAD OF COVID-19	Preprint
Al Youha, S.	2020	Validation of the Kuwait Progression Indicator Score for predicting progression of severity in COVID19	Preprint
Albi, G.	2020	Relaxing lockdown measures in epidemic outbreaks using selective socio-economic containment with uncertainty	Preprint
Albi, G.	2020	Control with uncertain data of socially structured compartmental epidemic models	Preprint
Aleta, A.	2020	A data-driven assessment of early travel restrictions related to the spreading of the novel COVID-19 within mainland China	Preprint
Aleta, A.	2020	Evaluation of the potential incidence of COVID-19 and effectiveness of contention measures in Spain: a data-driven approach	Preprint
Aleta, A.	2020	Modeling the impact of social distancing, testing, contact tracing and household quarantine on second-wave scenarios of the COVID-19 epidemic	Preprint
Alharbi, N.	2020	Predicting COVID-19 Pandemic in Saudi Arabia Using Modified Singular Spectrum Analysis	Preprint
Allali, M.	2020	Model of a Testing-and-Quarantine Strategy to Slow-Down the COVID-19 Outbreak in Guadeloupe	Preprint
Allali, M.	2020	Prediction of the time evolution of the COVID-19 disease in Guadeloupe with a stochastic evolutionary model	Preprint
Al-Shammari, A. A. A.	2020	Real-time tracking and forecasting of the COVID-19 outbreak in Kuwait: a mathematical modeling study	Preprint
Alshammari, F. S.	2020	A mathematical model to investigate the transmission of COVID-19 in the Kingdom of Saudi Arabia	Preprint
Amin, R.	2020	Geographical surveillance of COVID-19: Diagnosed cases and death in the United States	Preprint
Amyar, A.	2020	Multi-task Deep Learning Based CT Imaging Analysis For COVID-19: Classification and Segmentation	Preprint
Anderson, S. C.	2020	Estimating the impact of COVID-19 control measures using a Bayesian model of physical distancing	Preprint
Ankarali, H.	2020	Modeling and Short-Term Forecasts of Indicators for COVID-19 Outbreak in 25 Countries at the end of March	Preprint
Annan, J. D.	2020	Model calibration, nowcasting, and operational prediction of the COVID-19 pandemic	Preprint
Anne, R.	2020	ARIMA modelling of predicting COVID-19 infections	Preprint
Ansumali, S.	2020	A Very Flat Peak: Why Standard SEIR Models Miss the Plateau of COVID-19 Infections and How it can be Corrected	Preprint
Anzai, A.	2020	Assessing the impact of reduced travel on exportation dynamics of novel coronavirus infection (COVID-19)	Preprint
Ardabili, S. F.	2020	COVID-19 Outbreak Prediction with Machine Learning	Preprint

Arenas, A.	2020	A mathematical model for the spatiotemporal epidemic spreading of COVID19	Preprint
Arenas, A.	2020	Derivation of the effective reproduction number R for COVID-19 in relation to mobility restrictions and confinement	Preprint
Arif, M.	2020	Estimation of the Final Size of the COVID-19 Epidemic in Balochistan, Pakistan	Preprint
Arifin, W. N.	2020	A Susceptible-Infected-Removed (SIR) model of COVID-19 epidemic trend in Malaysia under Movement Control Order (MCO) using a data fitting approach	Preprint
Arneson, D.	2020	CovidCounties - an interactive, real-time tracker of the COVID-19 pandemic at the level of US counties	Preprint
Arroyo M. F.	2020	Tracking R of COVID-19: A New Real-Time Estimation Using the Kalman Filter	Preprint
Aslan, I. H.	2020	Modeling COVID-19: Forecasting and analyzing the dynamics of the outbreak in Hubei and Turkey	Preprint
Asteris, P. G.	2020	A Novel Heuristic Global Algorithm to Predict the COVID-19 Pandemic Trend	Preprint
Attanayake, A. M. C. H.	2020	Phenomenological Modelling of COVID-19 epidemics in Sri Lanka, Italy and Hebei Province of China	Preprint
Austin, D. J.	2020	Near- and forecasting the SARS-COV-2 epidemic requires a global view and multiple methods	Preprint
Ayubali, A. A.	2020	On predicting the novel COVID-19 human infections by using Infectious Disease modelling method in the Indian State of Tamil Nadu during 2020	Preprint
Azarafza, M.	2020	COVID-19 Infection Forecasting based on Deep Learning in Iran	Preprint
Baerwolff, G. K. F.	2020	A Contribution to the Mathematical Modeling of the Corona/COVID-19 Pandemic	Preprint
Baker, R. E.	2020	Susceptible supply limits the role of climate in the COVID-19 pandemic	Preprint
Balabdaoui, F.	2020	Age-stratified model of the COVID-19 epidemic to analyze the impact of relaxing lockdown measures: nowcasting and forecasting for Switzerland	Preprint
Baldé, M. A. M. T	2020	Fitting SIR model to COVID-19 pandemic data and comparative forecasting with machine learning	Preprint
Baldea, L.	2020	What Can We Learn from the Time Evolution of COVID-19 Epidemic in Slovenia?	Preprint
Barbarossa, M. V.	2020	A first study on the impact of current and future control measures on the spread of COVID-19 in Germany	Preprint
Barbarossa, M. V.	2020	The impact of current and future control measures on the spread of COVID-19 in Germany	Preprint
Barbero, C. A.	2020	A statistical forecast of LOW mortality and morbidity due to COVID-19, in ARGENTINA and other Southern Hemisphere countries	Preprint
Barbero, C. A.	2020	A statistical forecast of LOW mortality (< 400,000 deaths) due to COVID-19, for the whole WORLD	Preprint
Barkan, E.	2020	Comparison of SARS-CoV-2 Exit Strategies Building Blocks	Preprint
Barman, P.	2020	Mathematical framework to model Covid-19 daily deaths	Preprint
Barrett, K.	2020	Potential magnitude of COVID-19-induced healthcare resource depletion in Ontario, Canada	Preprint
Bartolomeo, N.	2020	Estimating the size of the COVID-19 outbreak in Italy: Application of an exponential decay model to the weighted and cumulative average daily growth rate	Preprint
Batista, M.	2020	Estimation of the final size of the COVID-19 epidemic	Preprint
Baumgartner, M. T.	2020	Social distancing and movement constraint as the most likely factors for COVID-19 outbreak control in Brazil	Preprint
Bayham, J.	2020	The Impact of School Closure for COVID-19 on the US Healthcare Workforce and the Net Mortality Effects	Preprint
Bayyurt, L.	2020	Forecasting of COVID-19 Cases and Deaths Using ARIMA Models	Preprint
Bej, S.	2020	The timing of contact restrictions and pro-active testing balances the socio-economic impact of a lockdown with the control of infections	Preprint
Bekker, A.	2020	Pitting the Gumbel and logistic growth models against one another to model COVID-19 spread	Preprint
Belfin, R. V.	2020	COVID-19 peak estimation and effect of nationwide lockdown in India	Preprint
Benatia, D.	2020	Estimating COVID-19 Prevalence in the United States: A Sample Selection Model Approach	Preprint
Bendtsen Cano, O.	2020	COVID-19 Modelling: the Effects of Social Distancing	Preprint
Berg de Almeida, G.	2020	Several countries in one: a mathematical modeling analysis for COVID-19 in inner Brazil	Preprint
Bergman, N. K.	2020	Mobility Levels and Covid-19 Transmission Rates	Preprint
Bhattacharjee, B.	2020	A HEURISTIC MODEL FOR SPREAD OF COVID-19 INFECTION CASES IN INDIA	Preprint
Bhattacharyya, C.	2020	Suppress, and not just flatten:Strategies for Rapid Suppression of COVID19 transmission in Small World Communities	Preprint
Bhola, J.	2020	Corona Epidemic in Indian context: Predictive Mathematical Modelling	Preprint
Bhutta, Z. A.	2020	Evaluation of effects of public health interventions on COVID-19 transmission for Pakistan: A mathematical simulation study	Preprint
Bicher, M. R.	2020	Agent-Based Simulation for Evaluation of Contact-Tracing Policies Against the Spread of SARS-CoV-2	Preprint
Bilal, U.	2020	Spatial Inequities in COVID-19 outcomes in Three US Cities	Preprint
Bilinski, A.	2020	Contact tracing strategies for COVID-19 containment with attenuated physical distancing	Preprint
Biswas, M. H. A.	2020	Modeling the Effective Control Strategy for Transmission Dynamics of Global Pandemic COVID-19	Preprint
Biswas, S.	2020	Risk Assessment of nCOVID-19 Pandemic In India: A Mathematical Model And Simulation	Preprint
Bliznashki, S.	2020	A Bayesian Logistic Growth Model for the Spread of COVID-19 in New York	Preprint
Blyuss, K. B.	2020	Effects of latency and age structure on the dynamics and containment of COVID-19	Preprint
Bock, W.	2020	Mitigation and herd immunity strategy for COVID-19 is likely to fail	Preprint
Bogacz, R.	2020	Estimating the probability of New Zealand regions being free from COVID-19 using a stochastic SEIR model	Preprint
Bokharaie, V. S.	2020	A Stratified Model to Quantify the Effects of Containment Policies on the Spread of COVID-19	Preprint
Boldog, P.	2020	Risk assessment of novel coronavirus COVID-19 outbreaks outside China	Preprint
Bonasera, A.	2020	Chaos, Percolation and the Coronavirus Spread: a two-step model	Preprint
Bongolan, V. P.	2020	Age-stratified Infection Probabilities Combined with Quarantine-Modified SEIR Model in the Needs Assessments for COVID-19	Preprint

Bordehore, C.	2020	Understanding COVID-19 spreading through simulation modeling and scenarios comparison: preliminary results	Preprint
Branas, C. C.	2020	Flattening the curve before it flattens us: hospital critical care capacity limits and mortality from novel coronavirus (SARS-CoV2) cases in US counties	Preprint
Brand, S. P. C.	2020	Forecasting the scale of the COVID-19 epidemic in Kenya	Preprint
Brauer, M.	2020	Global access to handwashing: implications for COVID-19 control in low-income countries	Preprint
Brett, T.	2020	COVID-19 herd immunity strategies: walking an elusive and dangerous tightrope	Preprint
Brown, P.	2020	Mortality from COVID-19 in 12 countries and 6 states of the United States	Preprint
Bryant, P.	2020	Estimating the impact of mobility patterns on COVID-19 infection rates in 11 European countries	Preprint
Buhat, C. A. H.	2020	A mathematical model of COVID-19 transmission between frontliners and the general public	Preprint
Bui, L. V.	2020	Estimation of the incubation period of SARS-CoV-2 in Vietnam	Preprint
Bulchandani, V. B.	2020	Digital Herd Immunity and COVID-19	Preprint
Bulut, T.	2020	New Epidemiological Model Suggestions Revealing Size of Epidemics Based on the COVID-19 Pandemic Example: Wavelength Models	Preprint
Buonomo, B.	2020	Modelling information-dependent social behaviors in response to lockdowns: the case of COVID-19 epidemic in Italy	Preprint
Burns, A.	2020	Symptom-Based Isolation Policies: Evidence from a Mathematical Model of Outbreaks of Influenza and COVID-19	Preprint
Burstyn, I.	2020	Towards reduction in bias in epidemic curves due to outcome misclassification through Bayesian analysis of time-series of laboratory test results: Case study of COVID-19 in Alberta Canada and Philadelphia, USA	Preprint
Caccavo, D.	2020	Chinese and Italian COVID-19 outbreaks can be correctly described by a modified SIRD model	Preprint
Canabarro, A.	2020	Data-Driven Study of the COVID-19 Pandemic via Age-Structured Modelling and Prediction of the Health System Failure in Brazil amid Diverse Intervention Strategies	Preprint
Caramelo, F.	2020	Estimation of risk factors for COVID-19 mortality - preliminary results	Preprint
Carcione, J. M.	2020	A simulation of a COVID-19 epidemic based on a deterministic SEIR model	Preprint
Cardinal, R. N.	2020	Simulating a community mental health service during the COVID-19 pandemic: effects of clinician-clinician encounters, clinician-patient-family encounters, symptom-triggered protective behaviour, and household clustering	Preprint
Carleton, T.	2020	Causal empirical estimates suggest COVID-19 transmission rates are highly seasonal	Preprint
Carletti, T.	2020	COVID-19: The unreasonable effectiveness of simple models	Preprint
Casey, M.	2020	Estimating pre-symptomatic transmission of COVID-19: a secondary analysis using published data	Preprint
Castro, M. C.	2020	Demand for hospitalization services for COVID-19 patients in Brazil	Preprint
Catak, M.	2020	Nonlinear Markov Chain Modelling of the Novel Coronavirus (Covid-19) Pandemic	Preprint
Catala, M.	2020	Empiric model for short-time prediction of COVID-19 spreading	Preprint
Celaschi, S.	2020	Quantifying Effects, Forecasting Releases, and Herd Immunity of the Covid-19 Epidemic in S. Paulo, Brazil	Preprint
Celik, S.	2020	MODELLING OF COVID-19 OUTBREAK INDICATORS IN CHINA BETWEEN JANUARY AND APRIL	Preprint
Chaigneau, P.	2020	When strong mitigation against a pandemic backfires	Preprint
Chakraborty, T.	2020	An integrated deterministic-stochastic approach for predicting the long-term trajectories of COVID-19	Preprint
Chakraborty, T.	2020	Real-time forecasts and risk assessment of novel coronavirus (COVID-19) cases: A data-driven analysis	Preprint
Chandra, S. K.	2020	Mathematical Model with Social Distancing Parameter for Early Estimation of COVID-19 Spread	Preprint
Chandra, V.	2020	Stochastic Compartmental Modelling of SARS-CoV-2 with Approximate Bayesian Computation	Preprint
Chang, M.-C.	2020	Modeling the impact of human mobility and travel restrictions on the potential spread of SARS-CoV-2 in Taiwan	Preprint
Chao, D. L.	2020	Modeling layered non-pharmaceutical interventions against SARS-CoV-2 in the United States with Corvid	Preprint
Charpentier, A.	2020	COVID-19 pandemic control: balancing detection policy and lockdown intervention under ICU sustainability	Preprint
Chen, B.	2020	Roles of meteorological conditions in COVID-19 transmission on a worldwide scale	Preprint
Chen, S.	2020	Mitigating COVID-19 outbreak via high testing capacity and strong transmission-intervention in the United States	Preprint
Chen, Y.	2020	Modeling COVID-19 Growing Trends to Reveal the Differences in the Effectiveness of Non-Pharmaceutical Interventions among Countries in the World	Preprint
Cheng, B.	2020	A fundamental model and predictions for the spread of the COVID-19 epidemic	Preprint
Cheng, Q.	2020	Towards a simulation framework for optimizing infectious disease surveillance: An information theoretic approach for surveillance system design	Preprint
Childs, M. L.	2020	The impact of long-term non-pharmaceutical interventions on COVID-19 epidemic dynamics and control	Preprint
Chin, E. T.	2020	Projected geographic disparities in healthcare worker absenteeism from COVID-19 school closures and the economic feasibility of child care subsidies: a simulation study	Preprint
Chin, E. T.	2020	Frequency of routine testing for SARS-CoV-2 to reduce transmission among workers	Preprint
Chinazzi, M.	2020	The effect of travel restrictions on the spread of the 2019 novel coronavirus (2019-nCoV) outbreak	Preprint
Chitanvis, S. M.	2020	Dynamical model for social distancing in the U.S. during the COVID-19 epidemic	Preprint
Chong, K. C.	2020	Monitoring Disease Transmissibility of 2019 Novel Coronavirus Disease in Zhejiang, China	Preprint
Chong, K. C.	2020	Transmissibility of coronavirus disease 2019 (COVID-19) in Chinese cities with different transmission dynamics of imported cases	Preprint
Chow, C. C.	2020	Global prediction of unreported SARS-CoV2 infection from observed COVID-19 cases	Preprint
Chowell, D.	2020	Sustainable social distancing through facemask use and testing during the Covid-19 pandemic	Preprint
Chowell, G.	2020	Getting to zero quickly in the 2019-nCov epidemic with vaccines or rapid testing	Preprint
Christian, C.	2020	COVID-19 in Canada: Predictions for the future and control lessons from Asia	Preprint

Chun, J. Y.	2020	Transmission onset distribution of COVID-19 in South Korea	Preprint
Cilloni, L.	2020	The potential impact of the COVID-19 pandemic on tuberculosis: a modelling analysis	Preprint
Cintra, P. H. P.	2020	Estimative of real number of infections by COVID-19 on Brazil and possible scenarios	Preprint
Citron, D. T.	2020	Comparing Metapopulation Dynamics of Infectious Diseases under Different Models of Human Movement	Preprint
Ciufolini, I.	2020	A Mathematical prediction of the time evolution of the Covid-19 pandemic in some countries of the European Union using Monte Carlo simulations	Preprint
Ciufolini, I.	2020	Prediction of the time evolution of the Covid-19 Pandemic in Italy by a Gauss Error Function and Monte Carlo simulations	Preprint
Cleary, B.	2020	Efficient prevalence estimation and infected sample identification with group testing for SARS-CoV-2	Preprint
Clifford, S. J.	2020	Effectiveness of interventions targeting air travellers for delaying local outbreaks of SARS-CoV-2	Preprint
Coelho, F. C.	2020	Assessing the potential impact of COVID-19 in Brazil: Mobility, Morbidity and the burden on the Health Care System	Preprint
Cohen, K.	2020	Suppressing the impact of the COVID-19 pandemic using controlled testing and isolation	Preprint
Colombo, R. M.	2020	An Age and Space Structured SIR Model Describing the Covid-19 Pandemic	Preprint
Contreras, S.	2020	Real-time estimation of R_0 for supporting public-health policies against COVID-19	Preprint
Costa, G. S.	2020	Metapopulation modeling of COVID-19 advancing into the countryside: an analysis of mitigation strategies for Brazil	Preprint
Cotta, R. M.	2020	Parametric identification and public health measures influence on the COVID-19 epidemic evolution in Brazil	Preprint
Courtney, J.	2020	COVID-19: Tracking the Pandemic with A Simple Curve Approximation Tool (SCAT)	Preprint
Crokidakis, N.	2020	COVID-19 spreading in Rio de Janeiro, Brazil: do the policies of social isolation really work?	Preprint
Cruz-Pacheco, G.	2020	Dispersion of a new coronavirus SARS-CoV-2 by airlines in 2020: Temporal estimates of the outbreak in Mexico	Preprint
Cuadrado, C.	2020	Impact of small-area lockdowns for the control of the COVID-19 pandemic	Preprint
Curmei, M.	2020	Estimating Household Transmission of SARS-CoV-2	Preprint
Daddi, E.	2020	Early forecasts of the evolution of the COVID-19 outbreaks and quantitative assessment of the effectiveness of countering measures	Preprint
Dana, S.	2020	Brazilian Modeling of COVID-19 (BRAM-COD): a Bayesian Monte Carlo approach for COVID-19 spread in a limited data set context	Preprint
Danon, L.	2020	A spatial model of CoVID-19 transmission in England and Wales: early spread and peak timing	Preprint
Das, S. K.	2020	Spread of COVID-19: Investigation of universal features in real data	Preprint
Dattilo, W.	2020	COVID-19 most vulnerable Mexican cities lack the public health infrastructure to face the pandemic: a new temporally-explicit model	Preprint
Daunizeau, J.	2020	On the reliability of model-based predictions in the context of the current COVID epidemic event: impact of outbreak peak phase and data paucity	Preprint
Davies, G.	2020	The Epidemic Severity Index: Estimating Relative Local Severity of Novel Disease Outbreaks	Preprint
Davies, N. G.	2020	Age-dependent effects in the transmission and control of COVID-19 epidemics	Preprint
Davies, N. G.	2020	The effect of non-pharmaceutical interventions on COVID-19 cases, deaths and demand for hospital services in the UK: a modelling study	Preprint
Davies, N. G.	2020	The impact of Coronavirus disease 2019 (COVID-19) on health systems and household resources in Africa and South Asia	Preprint
de Alcantara, L. R. P.	2020	Using different epidemiological models to modeling the epidemic dynamics in Brazil	Preprint
De Brouwer, E.	2020	Can herd immunity be achieved without breaking ICUs?	Preprint
De Brouwer, E.	2020	Modeling the COVID-19 outbreaks and the effectiveness of the containment measures adopted across countries	Preprint
de Leon, U. A. P.	2020	An SEIARD epidemic model for COVID-19 in Mexico: mathematical analysis and state-level forecast	Preprint
de Oliveira, A. C. S.	2020	Bayesian modeling of COVID-19 cases with a correction to account for under-reported cases	Preprint
de Oliveira, S. B.	2020	Monitoring social distancing and SARS-CoV-2 transmission in Brazil using cell phone mobility data	Preprint
de Souza, D. B.	2020	Using curvature to infer COVID-19 fractal epidemic network fragility and systemic risk	Preprint
de Vlas, S. J.	2020	A phased lift of control: a practical strategy to achieve herd immunity against Covid-19 at the country level	Preprint
Deasy, J.	2020	Forecasting ultra-early intensive care strain from COVID-19 in England	Preprint
de-Camino-Beck, T.	2020	A modified SEIR Model with Confinement and Lockdown of COVID-19 for Costa Rica	Preprint
Deforche, K.	2020	An age-structured epidemiological model of the Belgian COVID-19 epidemic	Preprint
Dehesh, T.	2020	Forecasting of COVID-19 Confirmed Cases in Different Countries with ARIMA Models	Preprint
Delius, G. W.	2020	More prevalent, less deadly? Bayesian inference of the COVID19 Infection Fatality Ratio from mortality data	Preprint
Dell'Anna, L.	2020	Solvable delay model for epidemic spreading: the case of Covid-19 in Italy	Preprint
Deo, V.	2020	Forecasting Transmission Dynamics of COVID-19 Epidemic in India under Various Containment Measures- A Time-Dependent State-Space SIR Approach	Preprint
Dey, S.	2020	MODELING COVID19 IN INDIA (MAR 3 - MAY 7, 2020): HOW FLAT IS FLAT, AND OTHER HARD FACTS	Preprint
Dhanwant, J. N.	2020	Quantitative Estimation of Disruption in Social Contact Structure and its Effect in COVID-19 Spread in India	Preprint
Di Domenico, L.	2020	Expected impact of lockdown in Ile-de-France and possible exit strategies	Preprint
Di Domenico, L.	2020	Expected impact of reopening schools after lockdown on COVID-19 epidemic in Ile-de-France	Preprint
Di Lauro, F.	2020	The timing of one-shot interventions for epidemic control	Preprint
Dias Junior, T.	2020	A Computational Model for Estimating the Evolution of COVID-19 in Rondonia-Brazil	Preprint
DiMaggio, C.	2020	Blacks/African Americans are 5 Times More Likely to Develop COVID-19: Spatial Modeling of New York City ZIP Code-level Testing Results	Preprint
Ding, G.	2020	Brief Analysis of the ARIMA model on the COVID-19 in Italy	Preprint
Diop, B. Z.	2020	The relatively young and rural population may limit the spread and severity of Covid-19 in Africa: a modelling study	Preprint

Djidjou-Demasse, R.	2020	Optimal COVID-19 epidemic control until vaccine deployment	Preprint
Dolbeault, J.	2020	Social heterogeneity and the COVID-19 lockdown in a multi-group SEIR model	Preprint
Donsimoni, J. R.	2020	Should contact bans be lifted in Germany? A quantitative prediction of its effects	Preprint
Donsimoni, J. R.	2020	Projecting the Spread of COVID19 for Germany	Preprint
Dorn, M.	2020	Hemogram Data as a Tool for Decision-making in COVID-19 Management: Applications to Resource Scarcity Scenarios	Preprint
Dropkin, G.	2020	COVID-19 UK Lockdown Forecasts and R0	Preprint
Duque, D.	2020	COVID-19: How to Relax Social Distancing If You Must	Preprint
Dy, L. F.	2020	A COVID-19 Infection Risk Model for Frontline Health Care Workers	Preprint
Dziugys, A.	2020	Simplified model of Covid-19 epidemic prognosis under quarantine and estimation of quarantine effectiveness	Preprint
Eastman, B.	2020	Mathematical modeling of COVID-19 containment strategies with considerations for limited medical resources	Preprint
Ediriweera, D. S.	2020	AN EPIDEMIOLOGICAL MODEL TO AID DECISION-MAKING FOR COVID-19 CONTROL IN SRI LANKA	Preprint
Eguiluz, V. M.	2020	Risk of secondary infection waves of COVID-19 in an insular region: the case of the Balearic Islands, Spain	Preprint
Egwolf, B.	2020	Mobility-Guided Modeling of the COVID-19 Pandemic in Metro Manila	Preprint
Eikenberry, S. E.	2020	To mask or not to mask: Modeling the potential for face mask use by the general public to curtail the COVID-19 pandemic	Preprint
Eilersen, A.	2020	Estimating cost-benefit of quarantine length for COVID-19 mitigation	Preprint
Einian, M.	2020	Modeling of COVID-19 Pandemic and Scenarios for Containment	Preprint
Eksin, C.	2020	Reacting to outbreaks at neighboring localities	Preprint
El Allaoui, A.	2020	A simple mathematical model for Coronavirus (COVID-19)	Preprint
El Desouky, E. D.	2020	Prediction of the Epidemic Peak of Covid19 in Egypt, 2020	Preprint
Emery, J. C.	2020	The contribution of asymptomatic SARS-CoV-2 infections to transmission - a model-based analysis of the Diamond Princess outbreak	Preprint
Eng, G.	2020	Initial Model for Impact of Social Distancing on CoVID-19 Spread	Preprint
Engbert, R.	2020	Sequential data assimilation of the stochastic SEIR epidemic model for regional COVID-19 dynamics	Preprint
Erandi, H.	2020	Effectiveness of the strategies implemented in Sri Lanka for controlling the COVID-19 outbreak	Preprint
Eshragh, A.	2020	Modeling the Dynamics of the COVID-19 Population in Australia: A Probabilistic Analysis	Preprint
Evans, S.	2020	The impact of testing and infection prevention and control strategies on within-hospital transmission dynamics of COVID-19 in English hospitals	Preprint
Evgeniou, T.	2020	Epidemic Models for Personalised COVID-19 Isolation and Exit Policies Using Clinical Risk Predictions	Preprint
Fang, H.	2020	Human Mobility Restrictions and the Spread of the Novel Coronavirus (2019-nCoV) in China	Preprint
Farseev, A.	2020	Understanding Economic and Health Factors Impacting the Spread of COVID-19 Disease	Preprint
Fefferman, N. H.	2020	Fear, Access, and the Real-Time Estimation of Etiological Parameters for Outbreaks of Novel Pathogens	Preprint
Feng, S.	2020	Prediction of the COVID-19 Epidemic Trends Based on SEIR and AI Models	Preprint
Fenga, L.	2020	CoViD-19: An Automatic, Semiparametric Estimation Method for the Population Infected in Italy	Preprint
Fenichel, E. P.	2020	A cell phone data driven time use analysis of the COVID-19 epidemic	Preprint
Fernandez-Recio, J.	2020	Modelling the evolution of COVID-19 in high-incidence European countries and regions: estimated number of infections and impact of past and future intervention measures	Preprint
Ferretti, L.	2020	Quantifying SARS-CoV-2 transmission suggests epidemic control with digital contact tracing	Preprint
Firth, J. A.	2020	Combining fine-scale social contact data with epidemic modelling reveals interactions between contact tracing, quarantine, testing and physical distancing for controlling COVID-19	Preprint
Fokas, A. S.	2020	COVID-19: Predictive Mathematical Models for the Number of Deaths in South Korea, Italy, Spain, France, UK, Germany, and USA	Preprint
Fokas, A. S.	2020	Predictive mathematical models for the number of individuals infected with COVID-19	Preprint
Fortaleza, C. M. C. B.	2020	Elementary spatial structures and dispersion of COVID-19: health geography directing responses to public health emergency in Sao Paulo State, Brazil	Preprint
Fowler, J. H.	2020	The Effect of Stay-at-Home Orders on COVID-19 Cases and Fatalities in the United States	Preprint
Francis, A.	2020	Projected ICU and Mortuary load due to COVID-19 in Sydney	Preprint
Frausto-Solis, J.	2020	The Hybrid Forecasting Method SVR-ESAR for Covid-19	Preprint
Freitas, A. S.	2020	New SIR model used in the projection of COVID 19 cases in Brazil	Preprint
Fu, X.	2020	Forecasting COVID-19 cases and deaths in epidemic-mitigating European countries by Richards function-based regression analyses	Preprint
Fudolig, M.	2020	The local stability of a modified multi-strain SIR model for emerging viral strains	Preprint
Ganem, F.	2020	The impact of early social distancing at COVID-19 Outbreak in the largest Metropolitan Area of Brazil	Preprint
Ganyani, T.	2020	Estimating the generation interval for COVID-19 based on symptom onset data	Preprint
Garbey, M.	2020	A Model of Workflow in the Hospital During a Pandemic to Assist Management	Preprint
Garcia-Iglesias, D.	2020	Early behavior of Madrid Covid-19 disease outbreak: A mathematical model	Preprint
Gardner, J. M.	2020	Intervention strategies against COVID-19 and their estimated impact on Swedish healthcare capacity	Preprint
Gasparek, M.	2020	A stochastic, individual-based model for the evaluation of the impact of non-pharmacological interventions on COVID-19 transmission in Slovakia	Preprint
Gathungu, D. K.	2020	Modeling the Effects of Non-Pharmaceutical Interventions on COVID-19 Spread in Kenya	Preprint
Gayawan, E.	2020	The spatio-temporal epidemic dynamics of COVID-19 outbreak in Africa	Preprint
Gel, E.	2020	COVID-19 Healthcare Demand Projections: Arizona	Preprint

Gelman, A.	2020	Bayesian analysis of tests with unknown specificity and sensitivity	Preprint
Georgiou, H. V.	2020	COVID-19 outbreak in Greece has passed its rising inflection point and stepping into its peak	Preprint
Gerasimov, A.	2020	Reaching collective immunity for COVID-19: an estimate with a heterogeneous model based on the data for Italy	Preprint
Gerasimov, A.	2020	COVID-19 DYNAMICS: A HETEROGENEOUS MODEL	Preprint
German, R.	2020	Modeling Exit Strategies from COVID-19 Lockdown with a Focus on Antibody Tests	Preprint
Gevertz, J.	2020	A novel COVID-19 epidemiological model with explicit susceptible and asymptomatic isolation compartments reveals unexpected consequences of timing social distancing	Preprint
Ghaffarzadegan, N.	2020	Simulation-based Estimation of the Spread of COVID-19 in Iran	Preprint
Ghosh, P.	2020	COVID-19 in India: State-wise Analysis and Prediction	Preprint
Ghosh, P.	2020	Increased Detection coupled with Social Distancing and Health Capacity Planning Reduce the Burden of COVID-19 Cases and Fatalities: A Proof of Concept Study using a Stochastic Computational Simulation Model	Preprint
Ghosh, S.	2020	Predictive Model with Analysis of the Initial Spread of COVID-19 in India	Preprint
Gjini, E.	2020	Modeling Covid-19 dynamics for real-time estimates and projections: an application to Albanian data	Preprint
Glass, D. H.	2020	European lockdowns and the consequences of relaxation during the COVID-19 pandemic	Preprint
Gloeckner, S.	2020	Now-casting the COVID-19 epidemic: The use case of Japan, March 2020	Preprint
Goetz, T.	2020	COVID-19 Disease Dynamics in Germany: First Models and Parameter Identification	Preprint
Goldenbogen, B.	2020	Geospatial precision simulations of community confined human interactions during SARS-CoV-2 transmission reveals bimodal intervention outcomes	Preprint
Goldsztejn, U.	2020	Public policy and economic dynamics of COVID-19 spread: a mathematical modeling study	Preprint
Gomez, J.	2020	INFEKTA: A General Agent-based Model for Transmission of Infectious Diseases: Studying the COVID-19 Propagation in Bogota - Colombia	Preprint
Gomez-Exposito, A.	2020	Monitoring and Tracking the Evolution of a Viral Epidemic Through Nonlinear Kalman Filtering: Application to the Covid-19 Case	Preprint
Gongalsky, M. B.	2020	Early detection of superspreaders by mass group pool testing can mitigate COVID-19 pandemic	Preprint
Gorji, H.	2020	STeCC: Smart Testing with Contact Counting Enhances Covid-19 Mitigation by Bluetooth App Based Contact Tracing	Preprint
Gostic, K.	2020	Estimated effectiveness of traveller screening to prevent international spread of 2019 novel coronavirus (2019-nCoV)	Preprint
Gountas, I.	2020	Act early, save lives: managing COVID-19 in Greece	Preprint
Grant, A.	2020	Dynamics of COVID-19 epidemics: SEIR models underestimate peak infection rates and overestimate epidemic duration	Preprint
Grewelle, R.	2020	Estimating the Global Infection Fatality Rate of COVID-19	Preprint
Griette, Q.	2020	Estimating the last day for COVID-19 outbreak in mainland China	Preprint
Griette, Q.	2020	Unreported cases for Age Dependent COVID-19 Outbreak in Japan	Preprint
Grimm, V.	2020	Extensions of the SEIR Model for the Analysis of Tailored Social Distancing and Tracing Approaches to Cope with COVID-19	Preprint
Grossmann, G.	2020	Importance of Interaction Structure and Stochasticity for Epidemic Spreading: A COVID-19 Case Study	Preprint
Gu, J.	2020	Better Strategies for Containing COVID-19 Epidemics --- A Study of 25 Countries via an Extended Varying Coefficient SEIR Model	Preprint
Gupta, A. K.	2020	Spatial Network based model forecasting transmission and control of COVID-19	Preprint
Gupta, M.	2020	Transmission dynamics of the COVID-19 epidemic in India and modelling optimal lockdown exit strategies	Preprint
Gupta, P.	2020	A data-driven method to detect the flattening of the COVID-19 pandemic curve and estimating its ending life-cycle using only the time-series of new cases per day	Preprint
Gupta, R.	2020	Trend Analysis and Forecasting of COVID-19 outbreak in India	Preprint
Gupta, R.	2020	SEIR and Regression Model based COVID-19 outbreak predictions in India	Preprint
Guttal, V.	2020	Risk assessment via layered mobile contact tracing for epidemiological intervention	Preprint
Guzzi, P. H.	2020	Intensive Care Unit Resource Planning During COVID-19 Emergency at the Regional Level: the Italian case	Preprint
Hackl, K.	2020	Modeling the COVID-19 pandemic - parameter identification and reliability of predictions	Preprint
Hamidouche, M.	2020	COVID-19 outbreak in Algeria: A mathematical Model to predict cumulative cases	Preprint
Hammoumi, A.	2020	A first study on the impact of containment measure on COVID-19 spread in Morocco	Preprint
Han, H.	2020	Estimate the incubation period of coronavirus 2019 (COVID-19)	Preprint
Hao, X.	2020	Full-spectrum dynamics of the coronavirus disease outbreak in Wuhan, China: a modeling study of 32,583 laboratory-confirmed cases	Preprint
Harbert, R. S.	2020	Spatial modeling cannot currently differentiate SARS-CoV-2 coronavirus and human distributions on the basis of climate in the United States	Preprint
Hasan, N. A.	2020	Predict the next moves of COVID-19: reveal the temperate and tropical countries scenario	Preprint
Hauser, A.	2020	Estimation of SARS-CoV-2 mortality during the early stages of an epidemic: a modelling study in Hubei, China and northern Italy	Preprint
Hayot Berk, S.	2020	Purely data-driven exploration of COVID-19 pandemic after three months of the outbreak	Preprint
Hazem, Y.	2020	Hasty Reduction of COVID-19 Lockdown Measures Leads to the Second Wave of Infection	Preprint
He, J.	2020	Comparative Analysis of COVID-19 Transmission Patterns in Three Chinese Regions vs. South Korea, Italy and Iran	Preprint
He, W.	2020	Estimation of the basic reproduction number, average incubation time, asymptomatic infection rate, and case fatality rate for COVID-19: Meta-analysis and sensitivity analysis	Preprint
Hébert-Dufresne, L.	2020	Beyond R0: Heterogeneity in secondary infections and probabilistic epidemic forecasting	Preprint
Hellewell, J.	2020	Feasibility of controlling 2019-nCoV outbreaks by isolation of cases and contacts	Preprint
Heriot, G. S.	2020	Tensions between research and public health: modelling the risks and benefits of SARS-CoV-2 vaccine field trials versus human infection challenge studies	Preprint
Hermanowicz, S. W.	2020	Simple model for Covid-19 epidemics - back-casting in China and forecasting in the US	Preprint

Hernandez, A.	2020	On the impact of early non-pharmaceutical interventions as containment strategies against the COVID-19 pandemic	Preprint
Hernandez, M.	2020	The Impact of Host-Based Early Warning on Disease Outbreaks	Preprint
Herrmann, H. A.	2020	Using network science to propose strategies for effectively dealing with pandemics: The COVID-19 example	Preprint
Hidaka, S.	2020	Predicting Long-term Evolution of COVID-19 by On-going Data using Bayesian Susceptible-Infected-Removed Model	Preprint
Hochberg, M. E.	2020	Importance of suppression and mitigation measures in managing COVID-19 outbreaks	Preprint
Hoertel, N.	2020	Facing the COVID-19 epidemic in NYC: a stochastic agent-based model of various intervention strategies	Preprint
Hoertel, N.	2020	Lockdown exit strategies and risk of a second epidemic peak: a stochastic agent-based model of SARS-CoV-2 epidemic in France	Preprint
Hoffman, B. U.	2020	Significant Relaxation of SARS-CoV-2-Targeted Non-Pharmaceutical Interventions Will Result in Profound Mortality: A New York State Modelling Study	Preprint
Hossain, M. P.	2020	The effects of border control and quarantine measures on global spread of COVID-19	Preprint
Hossein, R.	2020	Real-time time-series modelling for prediction of COVID-19 spread and intervention assessment	Preprint
Hou, J.	2020	Changing transmission dynamics of COVID-19 in China: a nationwide population-based piecewise mathematical modelling study	Preprint
Hu, F.-C.	2020	The Estimated Time-Varying Reproduction Numbers during the Ongoing Pandemic of the Coronavirus Disease 2019 (COVID-19) in 12 Selected Countries outside China	Preprint
Huang, N. E.	2020	Herd immunity vs suppressed equilibrium in COVID-19 pandemic: different goals require different models for tracking	Preprint
Huang, S.	2020	Evolving Epidemiology and Effect of Non-pharmaceutical Interventions on the Epidemic of Coronavirus Disease 2019 in Shenzhen, China	Preprint
Huang, Y.	2020	Comprehensive Investigation and Isolation have Effectively Suppressed the Spread of COVID-19	Preprint
Hughes, R. P.	2020	Impact of relaxing Covid-19 social distancing measures on rural North Wales: a simulation analysis	Preprint
Humphrey, L.	2020	A path out of COVID-19 quarantine: an analysis of policy scenarios	Preprint
Hunter, P. R.	2020	Impact of non-pharmaceutical interventions against COVID-19 in Europe: a quasi-experimental study	Preprint
Hyafil, A.	2020	Analysis of the impact of lockdown on the evolution Covid-19 epidemics in Spain	Preprint
Ibrahim, R. R.	2020	Forecasting the spread of COVID-19 in Nigeria using Box-Jenkins Modeling Procedure	Preprint
Islam, M. M.	2020	Modeling risk of infectious diseases: a case of Coronavirus outbreak in four countries	Preprint
Issanov, A.	2020	COVID-19 OUTBREAK IN POST-SOVIET STATES: MODELING THE BEST AND WORST POSSIBLE SCENARIOS	Preprint
Ives, A.	2020	State-by-State estimates of R0 at the start of COVID-19 outbreaks in the USA	Preprint
Izadi, N.	2020	The epidemiologic parameters for COVID-19: A Systematic Review and Meta-Analysis	Preprint
Jakhar, M.	2020	COVID-19 Epidemic Forecast in Different States of India using SIR Model	Preprint
James, A.	2020	Suppression and Mitigation Strategies for Control of COVID-19 in New Zealand	Preprint
James, A.	2020	A structured model for COVID-19 spread: modelling age and healthcare inequities	Preprint
Jamieson-Lane, A. D.	2020	The Effectiveness of Targeted Quarantine for Minimising Impact of COVID-19	Preprint
Jayatilleke, A. U.	2020	COVID-19 case forecasting model for Sri Lanka based on Stringency Index	Preprint
Jia, W.	2020	Extended SIR prediction of the epidemics trend of COVID-19 in Italy and compared with Hunan, China	Preprint
Jiang, X.	2020	How does the outbreak of 2019-nCoV spread in mainland China? A retrospective analysis of the dynamic transmission routes	Preprint
Jindal, V. K.	2020	Manifestations of mortality based global data of COVID-19; unifying global model through single parameter	Preprint
Jit, M.	2020	Estimating number of cases and spread of Coronavirus disease 2019 (COVID-19) in the United Kingdom using critical care admissions, February to March 2020	Preprint
Jo, H.	2020	Analysis of COVID-19 spread in South Korea using the SIR model with time-dependent parameters and deep learning	Preprint
Josse, J.	2020	ICU Bed Availability Monitoring and analysis in the Grand Est region of France during the COVID-19 epidemic	Preprint
Karatayev, V.	2020	The far side of the COVID-19 epidemic curve: local re-openings based on globally coordinated triggers may work best	Preprint
Karimuzzaman, M.	2020	Forecasting the COVID-19 Pandemic with Climate Variables for Top Five Burdening and Three South Asian Countries	Preprint
Karin, O.	2020	Adaptive cyclic exit strategies from lockdown to suppress COVID-19 and allow economic activity	Preprint
Karnakov, P.	2020	Data driven inference of the reproduction number (R0) for COVID-19 before and after interventions for 51 European countries	Preprint
Kassa, S.	2020	Analysis of the mitigation strategies for COVID-19: from mathematical modelling perspective	Preprint
Katul, G. G.	2020	Global convergence of COVID-19 basic reproduction number and estimation from early-time SIR dynamics	Preprint
Kaushik, G.	2020	Analysis of Effectiveness of Quarantine Measures in Controlling COVID-19	Preprint
Ke, R.	2020	Fast spread of COVID-19 in Europe and the US suggests the necessity of early, strong and comprehensive interventions	Preprint
Keeling, M. J.	2020	Predictions of COVID-19 dynamics in the UK: short-term forecasting and analysis of potential exit strategies	Preprint
Kempf, P.	2020	Six Scenarios for non-medical interventions in the SARS-CoV-2 epidemic	Preprint
Kerr, C. C.	2020	Covasim: an agent-based model of COVID-19 dynamics and interventions	Preprint
Keskinocak, P.	2020	The Impact of Social Distancing on COVID19 Spread: State of Georgia Case Study	Preprint
Ketcheson, D. I.	2020	Optimal control of an SIR epidemic through finite-time non-pharmaceutical intervention	Preprint
Ketcheson, D. I.	2020	Estimating and forecasting COVID-19 attack rates and mortality	Preprint
Khailaie, S.	2020	Estimate of the development of the epidemic reproduction number Rt from Coronavirus SARS-CoV-2 case data and implications for political measures based on prognostics	Preprint
Khan, I. M.	2020	COVID-19 in China: Risk Factors and R0 Revisited	Preprint
Kharroubi, S.	2020	Modeling and predicting the spread of COVID-19 in Lebanon: A Bayesian perspective	Preprint
khoj, H.	2020	Epidemic Situation and Forecasting of COVID-19 in Saudi Arabia using the SIR model	Preprint

Khosravi, A.	2020	The basic reproduction number and prediction of the epidemic size of the novel coronavirus (COVID-19) in Shahroud, Iran	Preprint
Kim, B.	2020	Estimation of the case fatality rate based on stratification for the COVID-19 outbreak	Preprint
Kim, S.-K.	2020	AAEDM: Theoretical Dynamic Epidemic Diffusion Model and Covid-19 Korea Pandemic Cases	Preprint
Kirpich, A.	2020	Development of an interactive, agent-based local stochastic model of COVID-19 transmission and evaluation of mitigation strategies illustrated for the state of Massachusetts, USA	Preprint
Kissler, S. M.	2020	Projecting the transmission dynamics of SARS-CoV-2 through the post-pandemic period	Preprint
Kissler, S. M.	2020	Social distancing strategies for curbing the COVID-19 epidemic	Preprint
Klabunde, T.	2020	How high and long will the COVID-19 wave be? A data-driven approach to model and predict the COVID-19 epidemic and the required capacity for the German health system	Preprint
Klausner, Z.	2020	A single holiday was the turning point of the COVID-19 policy of Israel	Preprint
Klement, E.	2020	Controlled Avalanche: A Regulated Voluntary Exposure Approach for Addressing Covid19	Preprint
Kluger, D. M.	2020	Impact of healthcare worker shift scheduling on workforce preservation during the COVID-19 pandemic	Preprint
Knoch, C.	2020	RSI model: COVID-19 in Germany Alternating quarantine episodes and normal episodes	Preprint
Kochanczyk, M.	2020	Accounting for super-spreading gives the basic reproduction number R0 of COVID-19 that is higher than initially estimated	Preprint
Kochanczyk, M.	2020	Dynamics of COVID-19 pandemic at constant and time-dependent contact rates	Preprint
Koehler-Rieper, F.	2020	A novel deterministic forecast model for COVID-19 epidemic based on a single ordinary integro-differential equation	Preprint
Kohanovski, I.	2020	Inferring the effective start dates of non-pharmaceutical interventions during COVID-19 outbreaks	Preprint
Komies, S.	2020	COVID-19 Outcomes in Saudi Arabia and the UK: A Tale of Two Kingdoms	Preprint
Kontis, V.	2020	Age- and sex-specific total mortality impacts of the early weeks of the Covid-19 pandemic in England and Wales: Application of a Bayesian model ensemble to mortality statistics	Preprint
Kosmidis, K.	2020	A Fractal kinetics SI model can explain the dynamics of COVID-19 epidemics	Preprint
Kot, A. D.	2020	Critical levels of mask efficiency and of mask adoption that theoretically extinguish respiratory virus epidemics	Preprint
Kraay, A. N. M.	2020	Modeling serological testing to inform relaxation of social distancing for COVID-19 control	Preprint
Kraemer, M. U. G.	2020	The effect of human mobility and control measures on the COVID-19 epidemic in China	Preprint
Kretzschmar, M. E.	2020	Time is of the essence: impact of delays on effectiveness of contact tracing for COVID-19	Preprint
Kretzschmar, M. E.	2020	Isolation and contact tracing can tip the scale to containment of COVID-19 in populations with social distancing	Preprint
Kucharski, A. J.	2020	Effectiveness of isolation, testing, contact tracing and physical distancing on reducing transmission of SARS-CoV-2 in different settings	Preprint
Kucharski, A. J.	2020	Early dynamics of transmission and control of COVID-19: a mathematical modelling study	Preprint
Kumar, A.	2020	Modeling geographical spread of COVID-19 in India using network-based approach	Preprint
Kumar, P.	2020	Forecasting the dynamics of COVID-19 Pandemic in Top 15 countries in April 2020: ARIMA Model with Machine Learning Approach	Preprint
Kumar, P.	2020	Forecasting COVID-19 impact in India using pandemic waves Nonlinear Growth Models	Preprint
Kumar, S.	2020	Predication of Pandemic COVID-19 situation in Maharashtra, India	Preprint
Kurita, J.	2020	Effect of emergency declaration for the COVID-19 outbreak in Tokyo, Japan in the first two weeks	Preprint
Kurita, J.	2020	Forecast of the COVID-19 outbreak and effects of self-restraint in going out in Tokyo, Japan	Preprint
Kurita, J.	2020	Estimation of protection for COVID-19 in children from epidemiological information and estimate effect of policy in Japan	Preprint
Kuzdeuov, A.	2020	A Network-Based Stochastic Epidemic Simulator: Controlling COVID-19 with Region-Specific Policies	Preprint
Kyagulanyi, A.	2020	RISK ANALYSIS AND PREDICTION FOR COVID19 DEMOGRAPHICS IN LOW RESOURCE SETTINGS USING A PYTHON DESKTOP APP AND EXCEL MODELS	Preprint
Labadin, J.	2020	Transmission Dynamics of COVID-19 in Malaysia Prior to the Movement Control Order	Preprint
Lacasa, L.	2020	A flexible load sharing system optimising ICU demand in the context of COVID-19 pandemic	Preprint
Lachmann, A.	2020	Correcting under-reported COVID-19 case numbers: estimating the true scale of the pandemic	Preprint
Lai, S.	2020	Effect of non-pharmaceutical interventions for containing the COVID-19 outbreak in China	Preprint
Lambert, A.	2020	A mathematical assessment of the efficiency of quarantining and contact tracing in curbing the COVID-19 epidemic	Preprint
Lancastle, N. M.	2020	Is the impact of social distancing on coronavirus growth rates effective across different settings? A non-parametric and local regression approach to test and compare the growth rate	Preprint
Lander, A. D.	2020	Releasing the lockdown in the UK Covid-19 epidemic: a stochastic model	Preprint
Lassoued, A.	2020	Dataset on the COVID-19 Pandemic Situation in Tunisia with application to SIR Model	Preprint
Last, M.	2020	The First Wave of COVID-19 in Israel - Initial Analysis of Publicly Available Data	Preprint
Lathika Rajendrakumar, A.	2020	Epidemic Landscape and Forecasting of SARS-CoV-2 in India	Preprint
Lavezzo, E.	2020	Suppression of COVID-19 outbreak in the municipality of Vo, Italy	Preprint
Lavielle, M.	2020	Extension of a SIR model for modelling the propagation of Covid-19 in several countries	Preprint
Lee, F. H.	2020	A Heuristic Model for Spreading of COVID 19 in Singapore	Preprint
Lee, S.	2020	COVID-19 Pandemic Response Simulation: Impact of Non-pharmaceutical Interventions on Ending Lockdowns	Preprint
Lee, S. Y.	2020	Estimation of COVID-19 spread curves integrating global data and borrowing information	Preprint
Lee, T.-W.	2020	Gaussian Statistics and Data-Assimilated Model of Mortality due to COVID-19: China, USA, Italy, Spain, UK, Iran, and the World Total	Preprint
Lemaitre, J. C.	2020	Assessing the impact of non-pharmaceutical interventions on SARS-CoV-2 transmission in Switzerland	Preprint
Lesniewski, A.	2020	Estimating population immunity without serological testing	Preprint

Levesque, J.	2020	A note on COVID-19 seroprevalence studies: a meta-analysis using hierarchical modelling	Preprint
Lewer, D.	2020	COVID-19 and homelessness in England: a modelling study of the COVID-19 pandemic among people experiencing homelessness, and the impact of a residential intervention to isolate vulnerable people and care for people with symptoms	Preprint
Li, D.	2020	Estimating the scale of COVID-19 Epidemic in the United States: Simulations Based on Air Traffic directly from Wuhan, China	Preprint
Li, D.	2020	Estimating the Efficacy of Traffic Blockage and Quarantine for the Epidemic Caused by 2019-nCoV (COVID-19)	Preprint
Li, J.	2020	Estimation of the epidemic properties of the 2019 novel coronavirus: A mathematical modeling study	Preprint
Li, L.	2020	Propagation analysis and prediction of the COVID-19	Preprint
Li, R.	2020	Prediction of the Epidemic of COVID-19 Based on Quarantined Surveillance in China	Preprint
Li, R.	2020	Substantial undocumented infection facilitates the rapid dissemination of novel coronavirus (COVID-19)	Preprint
Li, X.	2020	The lockdown of Hubei Province causing different transmission dynamics of the novel coronavirus (2019-nCoV) in Wuhan and Beijing	Preprint
Liang, J.	2020	The impacts of diagnostic capability and prevention measures on transmission dynamics of COVID-19 in Wuhan	Preprint
Lichtenthaler Filho, R.	2020	A dynamic model for Covid-19 in Brazil	Preprint
Liebig, J.	2020	The current state of COVID-19 in Australia: importation and spread	Preprint
Lin, F.	2020	Evaluating the different control policies for COVID-19 between mainland China and European countries by a mathematical model in the confirmed cases	Preprint
Lin, G.	2020	Explaining the Bomb-Like Dynamics of COVID-19 with Modeling and the Implications for Policy	Preprint
Lin, Q.	2020	Estimating the daily trend in the size of the COVID-19 infected population in Wuhan	Preprint
Lin, Y.-C.	2020	The spatiotemporal estimation of the dynamic risk and the international transmission of 2019 Novel Coronavirus (COVID-19) outbreak: A global perspective	Preprint
Linka, K.	2020	The reproduction number of COVID-19 and its correlation with public health interventions	Preprint
Linka, K.	2020	Outbreak dynamics of COVID-19 in Europe and the effect of travel restrictions	Preprint
Linton, N. M.	2020	Incubation Period and Other Epidemiological Characteristics of 2019 Novel Coronavirus Infections with Right Truncation: A Statistical Analysis of Publicly Available Case Data	Preprint
Liu, D.	2020	The impact of containment measures and air temperature on mitigating COVID-19 transmission: non-classical SEIR modeling and analysis	Preprint
Liu, K.	2020	How many COVID-19 cases could have been prevented in the US if its interventions were as effective as those in China and South Korea?	Preprint
Liu, P.	2020	Diminishing Marginal Benefit of Social Distancing in Balancing COVID-19 Medical Demand-to-Supply	Preprint
Liu, Q.	2020	Assessing the Tendency of 2019-nCoV (COVID-19) Outbreak in China	Preprint
Liu, Q.	2020	Assessing the Global Tendency of COVID-19 Outbreak	Preprint
Liu, Q.	2020	Transmission in Latent Period Causes A Large Number of Infected People in the United States	Preprint
Liu, T.	2020	Time-varying transmission dynamics of Novel Coronavirus Pneumonia in China	Preprint
Liu, X.	2020	Modeling the situation of COVID-19 and effects of different containment strategies in China with dynamic differential equations and parameters estimation	Preprint
Liu, Z.	2020	Predicting the cumulative number of cases for the COVID-19 epidemic in China from early data	Preprint
Liu, Z.	2020	Predicting the number of reported and unreported cases for the COVID-19 epidemics in China, South Korea, Italy, France, Germany and United Kingdom	Preprint
Liu, Z.	2020	Government Responses Matter: Predicting Covid-19 cases in US under an empirical Bayesian time series framework	Preprint
Locey, K.	2020	An Interactive Tool to Forecast US Hospital Needs in the Coronavirus 2019 Pandemic	Preprint
Lofgren, E.	2020	The Epidemiological Implications of Incarceration Dynamics in Jails for Community, Corrections Officer, and Incarcerated Population Risks from COVID-19	Preprint
Lokuge, K.	2020	Exit strategies: optimising feasible surveillance for detection, elimination and ongoing prevention of COVID-19 community transmission	Preprint
Loli Piccolomiini, E.	2020	Monitoring Italian COVID-19 spread by an adaptive SEIRD model	Preprint
Long, C.	2020	Forecasting the Cumulative Number of COVID-19 Deaths in China: a Boltzmann Function-based Modeling Study	Preprint
Lopez, L.	2020	The end of the social confinement in Spain and the COVID-19 re-emergence risk	Preprint
Lopez, L. R.	2020	A modified SEIR model to predict the COVID-19 outbreak in Spain and Italy: simulating control scenarios and multi-scale epidemics	Preprint
Lover, A. A.	2020	Sentinel Event Surveillance to Estimate Total SARS-CoV-2 Infections, United States	Preprint
Lu, X.	2020	Classification of COVID-19 in intensive care patients: towards rational and effective clinical triage	Preprint
Lu, Z.	2020	A fractional-order SEIHDR model for COVID-19 with inter-city networked coupling effects	Preprint
Lyra, W.	2020	COVID-19 pandemics modeling with SEIR(+CAQH), social distancing, and age stratification. The effect of vertical confinement and release in Brazil	Preprint
Lytras, T.	2020	Estimating the ascertainment rate of SARS-CoV-2 infection in Wuhan, China: implications for management of the global outbreak	Preprint
Ma, Z.	2020	A Simple Mathematical Model for Estimating the Inflection Points of COVID-19 Outbreaks	Preprint
Magal, P.	2020	Predicting the number of reported and unreported cases for the COVID-19 epidemic in South Korea, Italy, France and Germany	Preprint
Magdon-Ismail, M.	2020	Machine Learning the Phenomenology of COVID-19 From Early Infection Dynamics	Preprint
Mahmud, A.	2020	Applying the SEIR Model in Forecasting The COVID-19 Trend in Malaysia: A Preliminary Study	Preprint
Maier, B. F.	2020	Effective containment explains sub-exponential growth in confirmed cases of recent COVID-19 outbreak in Mainland China	Preprint
Majumdar, A.	2020	Heterogeneous contact networks in COVID-19 spreading: the role of social deprivation	Preprint
Makhoul, M.	2020	Epidemiological impact of SARS-CoV-2 vaccination: mathematical modeling analyses	Preprint
Maleewong, M.	2020	Time delay epidemic model for COVID-19	Preprint
Malhotra, B.	2020	Progression of COVID-19 in Indian States - Forecasting Endpoints Using SIR and Logistic Growth Models	Preprint
Mallela, A.	2020	Optimal Control applied to a SEIR model of 2019-nCoV with social distancing	Preprint

Mallow, P. J.	2020	When Second Best Might be the Best: Using Hospitalization Data to Monitor the Novel Coronavirus Pandemic	Preprint
Mamo, D. K.	2020	Model the transmission dynamics of COVID-19 propagation with public health intervention	Preprint
Mandal, M.	2020	COVID-19 pandemic scenario in India compared to China and rest of the world: a data driven and model analysis	Preprint
Mangiarotti, S.	2020	Chaos theory applied to the outbreak of Covid-19: an ancillary approach to decision-making in pandemic context	Preprint
Manou-Abi, S.	2020	Analysis of the COVID-19 epidemic in french overseas department Mayotte based on a modified deterministic and stochastic SEIR model	Preprint
Marsland, R.	2020	Data-driven modeling reveals a universal dynamic underlying the COVID-19 pandemic under social distancing	Preprint
Martinez Ruiz del Arbol, P.	2020	Comparison of epidemic control strategies using agent-based simulations	Preprint
Martinez-Loran, E. R.	2020	Multinational modeling of SARS-CoV-2 spreading dynamics: Insights on the heterogeneity of COVID-19 transmission and its potential healthcare burden	Preprint
Masjedi, H.	2020	Nowcasting and Forecasting the Spread of COVID-19 in Iran	Preprint
Maslov, S.	2020	Window of Opportunity for Mitigation to Prevent Overflow of ICU capacity in Chicago by COVID-19	Preprint
Massonnaud, C.	2020	COVID-19: Forecasting short term hospital needs in France	Preprint
Matrajt, L.	2020	Evaluating the effectiveness of social distancing interventions against COVID-19	Preprint
Mayorga, L.	2020	Detection and isolation of asymptomatic individuals can make the difference in COVID-19 epidemic management	Preprint
Mbabazi, F. K.	2020	A Mathematical Model Approach for Prevention and Intervention Measures of the COVID-19 Pandemic in Uganda	Preprint
Mbuvha, R.	2020	On Data-Driven Management of the COVID-19 Outbreak in South Africa	Preprint
Mbuvha, R.	2020	Bayesian Inference of COVID-19 Spreading Rates in South Africa	Preprint
McBryde, E. S.	2020	Flattening the curve is not enough, we need to squash it. An explainer using a simple model	Preprint
McCombs, A.	2020	A model-based evaluation of the efficacy of COVID-19 social distancing, testing and hospital triage policies	Preprint
McGeoch, M. W.	2020	COVID-19 Propagation and Mortality in a Two-Part Population	Preprint
McKeigue, P. M.	2020	Evaluation of "stratify and shield" as a policy option for ending the COVID-19 lockdown in the UK	Preprint
McRae, M. P.	2020	Clinical Decision Support Tool and Rapid Point-of-Care Platform for Determining Disease Severity in Patients with COVID-19	Preprint
Mehrotra, S.	2020	A Model for Supply-Chain Decisions for Resource Sharing with an Application to Ventilator Allocation to Combat COVID-19	Preprint
Menendez, J.	2020	Elementary time-delay dynamics of COVID-19 disease	Preprint
Meng, Z.	2020	Development and utilization of an intelligent application for aiding COVID-19 diagnosis	Preprint
Menkir, T. F.	2020	Estimating the number of undetected COVID-19 cases exported internationally from all of China	Preprint
Menon, A.	2020	Modelling and simulation of COVID-19 propagation in a large population with specific reference to India	Preprint
Messner, W.	2020	The Influence of Contextual Factors on the Initial Phases of the COVID-19 Outbreak across U.S. Counties	Preprint
Messner, W.	2020	Variation in COVID-19 Outbreaks at U.S. State and County Levels	Preprint
Meunier, T. A. J.	2020	Full lockdown policies in Western Europe countries have no evident impacts on the COVID-19 epidemic	Preprint
Michaels, J. A.	2020	Explaining national differences in the mortality of Covid-19: individual patient simulation model to investigate the effects of testing policy and other factors on apparent mortality	Preprint
Milani, F.	2020	COVID-19 Outbreak, Social Response, and Early Economic Effects: A Global VAR Analysis of Cross-Country Interdependencies	Preprint
Milano, M.	2020	Statistical and network-based analysis of Italian COVID-19 data: communities detection and temporal evolution	Preprint
Miller, A. C.	2020	Mobility trends provide a leading indicator of changes in SARS-CoV-2 transmission	Preprint
Miller, J. C.	2020	The risk of SARS-CoV-2 transmission in the healthcare setting and potential impact of cohorting strategies	Preprint
Milligan, W. R.	2020	Impact of essential workers in the context of social distancing for epidemic control	Preprint
Milne, G. J.	2020	The Effectiveness of Social Distancing in Mitigating COVID-19 Spread: a modelling analysis	Preprint
Milne, G. J.	2020	A Modelling Analysis of Strategies for Relaxing COVID-19 Social Distancing	Preprint
Ming, W.-K.	2020	Breaking down of healthcare system: Mathematical modelling for controlling the novel coronavirus (2019-nCoV) outbreak in Wuhan, China	Preprint
Mishra, S.	2020	Estimated surge in hospitalization and intensive care due to the novel coronavirus pandemic in the Greater Toronto Area, Canada: a mathematical modeling study with application at two local area hospitals	Preprint
Mizumoto, K.	2020	Transmission potential of the novel coronavirus (COVID-19) onboard the Diamond Princess Cruises Ship, 2020	Preprint
Mizumoto, K.	2020	Early epidemiological assessment of the transmission potential and virulence of coronavirus disease 2019 (COVID-19) in Wuhan City: China, January-February, 2020	Preprint
Młocek, W.	2020	Forecasting trajectories of an emerging epidemic with mathematical modeling in an online dashboard: The case of COVID-19	Preprint
Moghadami, M.	2020	Modeling the Corona Virus Outbreak in IRAN	Preprint
Mohd, M. H.	2020	Unraveling the Myths of R0 in Controlling the Dynamics of COVID-19 Outbreak: a Modelling Perspective	Preprint
Monleon-Getino, T.	2020	Next weeks of SARS-CoV-2: Projection model to predict time evolution scenarios of accumulated cases in Spain	Preprint
Mora, J. C.	2020	A Semiempirical Dynamical Model to Forecast the Propagation of Epidemics: The Case of the Sars-Cov-2 in Spain	Preprint
Moran, R. J.	2020	Estimating required lockdown cycles before immunity to SARS-CoV-2: Model-based analyses of susceptible population sizes, S0, in seven European countries including the UK and Ireland	Preprint
Moss, R.	2020	Modelling the impact of COVID-19 in Australia to inform transmission reducing measures and health system preparedness	Preprint
Mueller, M.	2020	Using random testing in a feedback-control loop to manage a safe exit from the COVID-19 lockdown	Preprint
Mujallad, A. F.	2020	Is there hope for the Hajj? Using the SIR Model to Forecast COVID-19 Progression in the City of Makkah	Preprint
Mukandavire, Z.	2020	Quantifying early COVID-19 outbreak transmission in South Africa and exploring vaccine efficacy scenarios	Preprint

Mukherji, N.	2020	The Social and Economic Factors Underlying the Incidence of COVID-19 Cases and Deaths in US Counties	Preprint
Munayco, C. V.	2020	Early transmission dynamics and control of COVID-19 in a southern hemisphere setting: Lima-Peru, February 29th-March 30th, 2020	Preprint
Muniz-Rodriguez, K.	2020	Transmission of SARS-CoV-2 in Georgia, USA, 2020	Preprint
Murray, C. J. L	2020	Forecasting COVID-19 impact on hospital bed-days, ICU-days, ventilator-days and deaths by US state in the next 4 months	Preprint
Murray, C. J. L.	2020	Forecasting the impact of the first wave of the COVID-19 pandemic on hospital demand and deaths for the USA and European Economic Area countries	Preprint
Nabi, K. N.	2020	FORECASTING COVID-19 PANDEMIC: A DATA-DRIVEN ANALYSIS	Preprint
Nagappa, B.	2020	Now casting and Forecasting of COVID-19 outbreak in the National Capital Region of Delhi	Preprint
Nail, B.	2020	A new design of an adaptive model of infectious diseases based on artificial intelligence approach: monitoring and forecasting of COVID-19 epidemic cases	Preprint
Naji, M.	2020	Exploring the spread dynamics of COVID-19 in Morocco	Preprint
Nakamoto, I.	2020	On the heterogeneity of infections, containment measures and the preliminary forecast of COVID-19 epidemic	Preprint
Naude, J.	2020	Worldwide Effectiveness of Various Non-Pharmaceutical Intervention Control Strategies on the Global COVID-19 Pandemic: A Linearised Control Model	Preprint
Neher, R. A.	2020	Potential impact of seasonal forcing on a SARS-CoV-2 pandemic	Preprint
Neil, M.	2020	Bayesian Network Analysis of Covid-19 data reveals higher Infection Prevalence Rates and lower Fatality Rates than widely reported	Preprint
Nekovee, M.	2020	Understanding the spreading patterns of COVID-19 in UK and its impact on exit strategies	Preprint
Nesteruk, I.	2020	Statistics based predictions of coronavirus 2019-nCoV spreading in mainland China	Preprint
Nesteruk, I.	2020	Stabilization of the coronavirus pandemic in Italy and global prospects	Preprint
Nesteruk, I.	2020	Hidden periods, duration and final size of COVID-19 pandemic	Preprint
Nesteruk, I.	2020	SIR-simulation of Corona pandemic dynamics in Europe	Preprint
Nesteruk, I.	2020	Long-term predictions for COVID-19 pandemic dynamics in Ukraine, Austria and Italy	Preprint
Neto, O. P.	2020	COVID-19 mathematical model reopening scenarios for Sao Paulo - Brazil	Preprint
Neto, O. P.	2020	Mathematical model of COVID-19 intervention scenarios for Sao Paulo- Brazil	Preprint
Neufeld, Z.	2020	Targeted adaptive isolation strategy for Covid-19 pandemic	Preprint
Neuwirth, C.	2020	Investigating duration and intensity of Covid-19 social-distancing strategies	Preprint
Ngonghala, C. N.	2020	Mathematical assessment of the impact of non-pharmaceutical interventions on curtailing the 2019 novel Coronavirus	Preprint
Nguyen, T. A.	2020	Modelling the impact of control measures against the COVID-19 pandemic in Viet Nam	Preprint
Niehus, R.	2020	Quantifying bias of COVID-19 prevalence and severity estimates in Wuhan, China that depend on reported cases in international travelers	Preprint
Nikolaou, M.	2020	Using Feedback on Symptomatic Infections to Contain the Coronavirus Epidemic: Insight from a SPIR Model	Preprint
Nikolaou, M.	2020	A Fundamental Inconsistency in the SIR Model Structure and Proposed Remedies	Preprint
Noll, N. B.	2020	COVID-19 Scenarios: an interactive tool to explore the spread and associated morbidity and mortality of SARS-CoV-2	Preprint
Nquemdjo, U. K.	2020	Simulating the progression of the COVID-19 disease in Cameroon using SIR models	Preprint
Nyabadza, F.	2020	Modelling the potential impact of social distancing on the COVID-19 epidemic in South Africa	Preprint
Odendaal, W. G.	2020	A Method to Model Outbreaks of New Infectious Diseases with Pandemic Potential such as COVID-19	Preprint
Ogbunugafor, C. B.	2020	The intensity of COVID-19 outbreaks is modulated by SARS-CoV-2 free-living survival and environmental transmission	Preprint
Oliveira, G.	2020	Refined compartmental models, asymptomatic carriers and COVID-19	Preprint
Oliveira, J. F.	2020	Evaluating the burden of COVID-19 on hospital resources in Bahia, Brazil: A modelling-based analysis of 14.8 million individuals	Preprint
Oliveiros, B.	2020	Role of temperature and humidity in the modulation of the doubling time of COVID-19 cases	Preprint
Oluyori, D. A.	2020	Global Analysis of an SEIRS Model for COVID-19 Capturing Saturated Incidence with Treatment Response	Preprint
Ortiz, M. R.	2020	Modeling the COVID-19 outbreak in Ecuador: Is it the right time to lift social distancing containment measures?	Preprint
Ozair, M.	2020	Estimation of Transmission Potential and Severity of COVID-19 in Romania and Pakistan	Preprint
Pais, R. J.	2020	Predicting the evolution and control of COVID-19 pandemic in Portugal	Preprint
Pal, D.	2020	Mathematical Analysis of a COVID-19 Epidemic Model by using Data Driven Epidemiological Parameters of Diseases Spread in India	Preprint
Pan, H.	2020	Multi-chain Fudan-CCDC model for COVID-19 -- a revisit to Singapore's case	Preprint
Pan, J.	2020	Effectiveness of control strategies for Coronavirus Disease 2019: a SEIR dynamic modeling study	Preprint
Pandey, K. R.	2020	COVID-19 Control Strategies and Intervention Effects in Resource Limited Settings: A Modeling Study	Preprint
Pant, R.	2020	COVID-19 Epidemic Dynamics and Population Projections from Early Days of Case Reporting in a 40 million population from Southern India	Preprint
Park, S. W.	2020	Reconciling early-outbreak estimates of the basic reproductive number and its uncertainty: framework and applications to the novel coronavirus (SARS-CoV-2) outbreak	Preprint
Paroli, M.	2020	Predicting SARS-CoV-2 infection trend using technical analysis indicators	Preprint
Pasayat, A. K.	2020	Predicting the COVID-19 positive cases in India with concern to Lockdown by using Mathematical and Machine Learning based Models	Preprint
Patil, N. L.	2020	Current State and Predicting Future Scenario of Highly Infected Nations for COVID-19 Pandemic	Preprint
Patterson-Lomba, O.	2020	Optimal timing for social distancing during an epidemic	Preprint
Paul, A.	2020	Prediction on Covid-19 epidemic for different countries: Focusing on South Asia under various precautionary measures	Preprint
Peak, C. M.	2020	Comparative Impact of Individual Quarantine vs. Active Monitoring of Contacts for the Mitigation of COVID-19: a modelling study	Preprint
Pedersen, M. G.	2020	A simple method to quantify country-specific effects of COVID-19 containment measures	Preprint

Pei, S.	2020	Differential Effects of Intervention Timing on COVID-19 Spread in the United States	Preprint
Pei, S.	2020	Initial Simulation of SARS-CoV2 Spread and Intervention Effects in the Continental US	Preprint
Peirlinck, M.	2020	Visualizing the invisible: The effect of asymptomatic transmission on the outbreak dynamics of COVID-19	Preprint
Peixoto, P. S.	2020	Potential dissemination of epidemics based on Brazilian mobile geolocation data. Part I: Population dynamics and future spreading of infection in the states of Sao Paulo and Rio de Janeiro during the pandemic of COVID-19	Preprint
Pejman, M. M.	2020	Coronavirus epidemic: prediction and controlling measures	Preprint
Peng, B.	2020	Population simulations of COVID-19 outbreaks provide tools for risk assessment and continuity planning	Preprint
Peng, L.	2020	Epidemic analysis of COVID-19 in China by dynamical modeling	Preprint
Perera, S. N.	2020	Mathematical Model to Study Early COVID-19 Transmission Dynamics in Sri Lanka	Preprint
Perez-Reche, F. J.	2020	Importance of untested infectious individuals for the suppression of COVID-19 epidemics	Preprint
Perkins, A.	2020	Estimating unobserved SARS-CoV-2 infections in the United States	Preprint
Perkins, A.	2020	Optimal control of the COVID-19 pandemic with non-pharmaceutical interventions	Preprint
Pham, T. M.	2020	The Potential Impact of Intensified Community Hand Hygiene Interventions on Respiratory tract Infections: A Modelling Study	Preprint
Phipps, S. J.	2020	Estimating the true (population) infection rate for COVID-19: A Backcasting Approach with Monte Carlo Methods	Preprint
Pitzer, V. E.	2020	The impact of changes in diagnostic testing practices on estimates of COVID-19 transmission in the United States	Preprint
Plank, M. J.	2020	A stochastic model for COVID-19 spread and the effects of Alert Level 4 in Aotearoa New Zealand	Preprint
Platt, D. E.	2020	Lies, Gosh Darn Lies, and Not Enough Good Statistics: Why Epidemic Model Parameter Estimation Fails	Preprint
Pongkitivanichkul, C.	2020	Estimating the size of COVID-19 epidemic outbreak	Preprint
Pourghasemi, H. R.	2020	Assessment of the outbreak risk, mapping and infestation behavior of COVID-19: Application of the autoregressive and moving average (ARMA) and polynomial models	Preprint
Prabhakaran, H.	2020	Spread of the Novel Coronavirus (SARS-CoV-2): Modeling and Simulation of Control Strategies	Preprint
Prague, M.	2020	Population modeling of early COVID-19 epidemic dynamics in French regions and estimation of the lockdown impact on infection rate	Preprint
Prakash, M. K.	2020	A minimal and adaptive prediction strategy for critical resource planning in a pandemic	Preprint
Prem, K.	2020	The effect of control strategies that reduce social mixing on outcomes of the COVID-19 epidemic in Wuhan, China	Preprint
Prince, M. J.	2020	The first month of the COVID-19 outbreak in 46 sub-Saharan African countries; a comparative analysis of growth rates	Preprint
Proverbio, D.	2020	Assessing suppression strategies against epidemic outbreaks like COVID-19: the SPQEIR model	Preprint
Pujari, B. S.	2020	Multi-city modeling of epidemics using spatial networks: Application to 2019-nCov (COVID-19) coronavirus in India	Preprint
Qiu, T.	2020	Revealing the influence of national public health policies for the outbreak of the SARS-CoV-2 epidemic in Wuhan, China through status dynamic modeling	Preprint
Qiu, Y.	2020	Impacts of social and economic factors on the transmission of coronavirus disease (COVID-19) in China	Preprint
Quadrat, J.-P.	2020	1-C Nonlinear Covid-19 Epidemic Model and Application to the Epidemic Prediction in France	Preprint
Quadrat, J.-P.	2020	A simple Covid-19 Epidemic Model and Containment Policy in France	Preprint
Quilty, B.	2020	Effectiveness of airport screening at detecting travellers infected with 2019-nCoV	Preprint
Rad, H. A.	2020	A study on control of novel corona-virus (2019-nCoV) disease process by using PID controller	Preprint
Raghavan, M.	2020	Using epidemic simulators for monitoring an ongoing epidemic	Preprint
Rahman, A.	2020	Modelling the transmission dynamics of COVID-19 in six high burden countries	Preprint
Rahman, M. M.	2020	Impact of control strategies on COVID-19 pandemic and the SIR model based forecasting in Bangladesh	Preprint
Rai, B.	2020	COVID-19 in India: Predictions, Reproduction Number and Public Health Preparedness	Preprint
Raimúndez, E.	2020	COVID-19 outbreak in Wuhan demonstrates the limitations of publicly available case numbers for epidemiological modelling	Preprint
Rajesh, A.	2020	CoVID-19 prediction for India from the existing data and SIR(D) model study	Preprint
Ramírez-Aldana, R.	2020	Spatial analysis of COVID-19 spread in Iran: Insights into geographical and structural transmission determinants at a province level	Preprint
Ranjan, R.	2020	Predictions for COVID-19 outbreak in India using Epidemiological models	Preprint
Ranjan, R.	2020	Estimating the Final Epidemic Size for COVID-19 Outbreak using Improved Epidemiological Models	Preprint
Rao, A. S. R. S.	2020	Continued and Serious Lockdown Could Minimize Many Newly Transmitted Cases of COVID-19 in the U.S.: Wavelets, Deterministic Models, and Data	Preprint
Rao, A. S. R. S.	2020	Immediate and Near Future Prediction of COVID-19 Patients in the U.S. Population Aged 65+ With the Prior Medical Conditions of Hypertension, Cardiovascular and Lung Diseases: Methods, Models and Acute Care Estimates	Preprint
Ravinder, R.	2020	An Adaptive, Interacting, Cluster-Based Model Accurately Predicts the Transmission Dynamics of COVID-19	Preprint
Rawson, T.	2020	How and when to end the COVID-19 lockdown: an optimisation approach	Preprint
Ray, D.	2020	Predictions, role of interventions and effects of a historic national lockdown in India's response to the COVID-19 pandemic: data science call to arms	Preprint
Rayo, J. F.	2020	Modeling the dynamics of COVID-19 using Q-SEIR model with age-stratified infection probability	Preprint
Razzak, J. A.	2020	ESTIMATING COVID-19 INFECTIONS IN HOSPITAL WORKERS IN THE UNITED STATES	Preprint
Read, J. M.	2020	Novel coronavirus 2019-nCoV: early estimation of epidemiological parameters and epidemic predictions	Preprint
Renna, I.	2020	When will the Covid-19 epidemic fade out?	Preprint
Ribeiro, S. P.	2020	Severe airport sanitarian control could slow down the spreading of COVID-19 pandemics in Brazil	Preprint
Riccio, A.	2020	Analysis of the SARS-Cov-2 epidemic in Lombardy (Italy) in its early phase. Are we going in the right direction?	Preprint

Ricoca Peixoto, V.	2020	Rapid assessment of the impact of lockdown on the COVID-19 epidemic in Portugal	Preprint
Rinaldi, G.	2020	An empirical estimate of the infection fatality rate of COVID-19 from the first Italian outbreak	Preprint
Rios, V.	2020	Is there a link between temperatures and COVID-19 contagions? Evidence from Italy	Preprint
Riou, J.	2020	Pattern of early human-to-human transmission of Wuhan 2019-nCoV	Preprint
Rivera-Rodriguez, C.	2020	Modelling strategies to predict hospital demand during the COVID-19 outbreak in Bogota, Colombia	Preprint
Rocha Filho, T. M.	2020	Expected impact of COVID-19 outbreak in a major metropolitan area in Brazil	Preprint
Roche, B.	2020	Optimal strategies for quarantine stopping in France. General expected patterns of strategies focusing on contact between age groups	Preprint
Rocklov, J.	2020	COVID-19 healthcare demand and mortality in Sweden in response to non-pharmaceutical (NPIs) mitigation and suppression scenarios	Preprint
Rodrigues Silva, R.	2020	A Bayesian analysis of the total number of cases of the COVID 19 when only a few data is available. A case study in the state of Goias, Brazil	Preprint
Rodriguez Llanes, J. M.	2020	Confronting COVID-19: Surging critical care capacity in Italy	Preprint
Rodriguez, J.	2020	A mechanistic population balance model to evaluate the impact of interventions on infectious disease outbreaks: Case for COVID19	Preprint
Rojas, J. H.	2020	Mathematical Modeling & the Transmission Dynamics of SARS-CoV-2 in Cali, Colombia: Implications to a 2020 Outbreak & public health preparedness	Preprint
Rojas-Gallardo, D. M.	2020	COVID-19 in Latin America: Contrasting phylodynamic inference with epidemiological surveillance	Preprint
Romero-Severson, E. O.	2020	Decline in global transmission rates of COVID-19 through May 6 2020	Preprint
Roques, L.	2020	Effect of a one-month lockdown on the epidemic dynamics of COVID-19 in France	Preprint
Roques, L.	2020	Using early data to estimate the actual infection fatality ratio from COVID-19 in France	Preprint
Rossberg, A. G.	2020	How will this continue? Modelling interactions between the COVID-19 pandemic and policy responses	Preprint
Rossi, N.	2020	Describing the COVID-19 Outbreak - Fitting Modified SIR models to Data	Preprint
Roux, J.	2020	COVID-19: One-month impact of the French lockdown on the epidemic burden	Preprint
Rovetta, A.	2020	Modelling the epidemiological trend and behavior of COVID-19 in Italy	Preprint
Roy, S.	2020	COVID-19 pandemic: Impact of lockdown, contact and non-contact transmissions on infection dynamics	Preprint
Rudnicki, W. R.	2020	Estimate of Covid-19 prevalence using imperfect data	Preprint
Rui, M.	2020	A Sparse Gaussian Network Model for Prediction the Growth Trend of COVID-19 Overseas Import Case: When can Hong Kong Lift the International Traffic Blockad?	Preprint
Russo, L.	2020	Tracing DAY-ZERO and Forecasting the COVID-19 Outbreak in Lombardy, Italy: A Compartmental Modelling and Numerical Optimization Approach	Preprint
Ryu, S.	2020	Estimate number of individuals infected with the 2019-novel coronavirus in South Korea due to the influx of international students from countries with virus risk: a simulation study	Preprint
Saberi, M.	2020	Accounting for underreporting in mathematical modelling of transmission and control of COVID-19 in Iran	Preprint
Sahafizadeh, E.	2020	Estimating the reproduction number of COVID-19 in Iran using epidemic modeling	Preprint
Saif, F.	2020	COVID-19 Pandemic in Pakistan: Stages and Recommendations	Preprint
Saif, F.	2020	Signature of State measures on the COVID-19 Pandemic in China, Italy, and USA	Preprint
Salim, N.	2020	COVID-19 epidemic in Malaysia: Impact of lock-down on infection dynamics	Preprint
Salje, H.	2020	Estimating the burden of SARS-CoV-2 in France	Preprint
Salvatoni, A.	2020	HOW THE COVID-19 PANDEMIA IS SPREADING IN ITALY	Preprint
Sanchez-Romero, M.	2020	How many lives can be saved? A global view on the impact of testing, herd immunity and demographics on COVID-19 fatality rates	Preprint
Sanchez-Taltavull, D.	2020	Modelling strategies to organize healthcare workforce during pandemics: application to COVID-19	Preprint
Savini, L.	2020	A municipality-based approach using commuting census data to characterise the vulnerability to influenza-like epidemic: the COVID-19 application in Italy	Preprint
Scala, A.	2020	Between Geography and Demography: Key Interdependencies and Exit Mechanisms for Covid-19	Preprint
Schröder, M.	2020	COVID-19 in Africa -- outbreak despite interventions?	Preprint
Schuttler, J.	2020	Covid-19 predictions using a Gauss model, based on data from April 2	Preprint
Schwartz, I. B.	2020	Predicting the impact of asymptomatic transmission, non-pharmaceutical intervention and testing on the spread of COVID19	Preprint
Sears, J.	2020	Are we #stayinghome to Flatten the Curve?	Preprint
Sedov, L.	2020	Modeling quarantine during epidemics and mass-testing using drones	Preprint
Sha, D.	2020	Spatiotemporal Analysis of Medical Resource Deficiencies in the U.S. under COVID-19 Pandemic	Preprint
Shah, N. H.	2020	Modelling COVID-19 Transmission in the United States through Interstate and Foreign Travels and Evaluating Impact of Governmental Public Health Interventions	Preprint
Shah, N. H.	2020	Control Strategies to Curtail Transmission of COVID-19	Preprint
Shah, N. H.	2020	Modeling the Impact of Nationwide BCG Vaccine Recommendations on COVID-19 Transmission, Severity, and Mortality	Preprint
Shah, P. V.	2020	Prediction of the Peak, Effect of Intervention and Total Infected by the Coronavirus Disease in India	Preprint
Shanlang, L.	2020	Research on the Influence of Information Diffusion on the Transmission of the Novel Coronavirus (COVID-19)	Preprint
Shao, N.	2020	The reproductive number R0 of COVID-19 based on estimate of a statistical time delay dynamical system	Preprint
Shao, N.	2020	CoVID-19 in Japan: What could happen in the future?	Preprint
Shao, P.	2020	Impact of city and residential unit lockdowns on prevention and control of COVID-19	Preprint
Shao, P.	2020	Beware of asymptomatic transmission: Study on 2019-nCoV prevention and control measures based on extended SEIR model	Preprint
Shardlow, A. M.	2020	The Impact of Social Distancing on The Course of The Covid-19 Pandemic in Four European Countries	Preprint

Sharifi, H.	2020	Estimating the number of COVID-19-related infections, deaths and hospitalizations in Iran under different physical distancing and isolation scenarios: A compartmental mathematical modeling	Preprint
Shayak, B.	2020	Transmission Dynamics of COVID-19 and Impact on Public Health Policy	Preprint
Shen, M.	2020	Modelling the epidemic trend of the 2019 novel coronavirus outbreak in China	Preprint
Sherpa, D.	2020	Estimating Impact of Austerity policies in COVID-19 fatality rates: Examining the dynamics of economic policy and Case Fatality Rates (CFR) of COVID-19 in OECD countries	Preprint
Shi, P.	2020	SEIR Transmission dynamics model of 2019 nCoV coronavirus with considering the weak infectious ability and changes in latency duration	Preprint
Shi, Z.	2020	Temporal relationship between outbound traffic from Wuhan and the 2019 coronavirus disease (COVID-19) incidence in China	Preprint
Shim, E.	2020	Estimating the risk of COVID-19 death during the course of the outbreak in Korea, February-May, 2020	Preprint
Shlomai, A.	2020	Global versus focused isolation during the SARS-CoV-2 pandemic-A cost-effectiveness analysis	Preprint
Shuler, R. L.	2020	Partial unlock model for COVID-19 or similar pandemic averts medical and economic disaster	Preprint
Siedner, M. J.	2020	Social distancing to slow the U.S. COVID-19 epidemic: an interrupted time-series analysis	Preprint
Silva, P. J. S.	2020	Robot dance: a city-wise automatic control of Covid-19 mitigation levels	Preprint
Silva, T. C.	2020	Quantitative Analysis of the Effectiveness of Public Health Measures on COVID-19 Transmission	Preprint
Silveira, A.	2020	Estimation and monitoring of COVID-19 transmissibility from publicly available data	Preprint
Singh, A.	2020	Is this the beginning or the end of COVID-19 outbreak in India? A data driven mathematical model-based analysis	Preprint
Singh, A.	2020	Modelling and data-based analysis of COVID-19 outbreak in India: a study on impact of social distancing measures	Preprint
Singh, A.	2020	Study of Non-Pharmacological Interventions on COVID-19 Spread	Preprint
Singh, B. P.	2020	Forecasting Novel Corona Positive Cases in India using Truncated Information: A Mathematical Approach	Preprint
Singh, B. P.	2020	Modeling Tempo of COVID-19 Pandemic in India and Significance of Lockdown	Preprint
Singh, J.	2020	Mathematical Model Based COVID-19 Prediction in India and its Different States	Preprint
Siraj, A.	2020	Early estimates of COVID-19 infections in small, medium and large population clusters	Preprint
Siwiak, M. M.	2020	From a single host to global spread. The global mobility based modelling of the COVID-19 pandemic implies higher infection and lower detection rates than current estimates	Preprint
Smeets, B.	2020	Scaling analysis of COVID-19 spreading based on Belgian hospitalization data	Preprint
Sofonea, M. T.	2020	Epidemiological monitoring and control perspectives: application of a parsimonious modelling framework to the COVID-19 dynamics in France	Preprint
Song, P. X.	2020	An epidemiological forecast model and software assessing interventions on COVID-19 epidemic in China	Preprint
Sosa, W.	2020	A non-parametric mathematical model to investigate the dynamic of a pandemic	Preprint
Soubeyrand, S.	2020	The current COVID-19 wave will likely be mitigated in the second-line European countries	Preprint
Spousta, M.	2020	Parametric analysis of early data on COVID-19 expansion in selected European countries	Preprint
Srivastava, A.	2020	Agent-Level Pandemic Simulation (ALPS) for Analyzing Effects of Lockdown Measures	Preprint
Stojkoski, V.	2020	The socio-economic determinants of the coronavirus disease (COVID-19) pandemic	Preprint
Struben, J.	2020	The December 2019 New Corona Virus (SARS-CoV-2) Outbreak: A Behavioral Infectious Disease Policy Model	Preprint
Sturniolo, S.	2020	Testing, tracing and isolation in compartmental models	Preprint
Su, L.	2020	Evaluation of the secondary transmission pattern and epidemic prediction of COVID-19 in the four metropolitan areas of China	Preprint
Sugishita, Y.	2020	Preliminary evaluation of voluntary event cancellation as a countermeasure against the COVID-19 outbreak in Japan as of 11 March, 2020	Preprint
Suhail, Y.	2020	Incorporating and Addressing Testing Bias Within Estimates of Epidemic Dynamics for SARS-CoV-2	Preprint
Sun, H.	2020	Tracking and Predicting COVID-19 Epidemic in China Mainland	Preprint
Sun, P.	2020	An SEIR Model for Assessment of Current COVID-19 Pandemic Situation in the UK	Preprint
Sun, S.	2020	Prediction of 2019-nCov in Italy based on PSO and inversion analysis	Preprint
Sun, Y.	2020	The effect of non-pharmaceutical interventions (NPIs) on the spread of COVID-19 pandemic in Japan: A modeling study	Preprint
Supino, M.	2020	The effects of containment measures in the Italian outbreak of COVID-19	Preprint
Syage, J. A.	2020	A Real-Time Statistical Model for Tracking and Forecasting COVID-19 Deaths, Prevalence and Incidence	Preprint
Syed, F.	2020	Estimation of the Final Size of the COVID-19 Epidemic in Pakistan	Preprint
Taboe, B. H.	2020	Predicting COVID-19 spread and public health needs to contain the pandemic in West-Africa	Preprint
Tang, B.	2020	Lessons drawn from China and South Korea for managing COVID-19 epidemic: insights from a comparative modeling study	Preprint
Tang, K.	2020	Novel Coronavirus 2019 (Covid-19) epidemic scale estimation: topological network-based infection dynamic model	Preprint
Tang, S.	2020	Stochastic discrete epidemic modeling of COVID-19 transmission in the Province of Shaanxi incorporating public health intervention and case importation	Preprint
Tang, Z.	2020	Prediction of New Coronavirus Infection Based on a Modified SEIR Model	Preprint
Tao, Y.	2020	Maximum entropy method for estimating the reproduction number: An investigation for COVID-19 in China	Preprint
Teles, P.	2020	PREDICTING THE EVOLUTION OF COVID-19 IN PORTUGAL USING AN ADAPTED SIR MODEL PREVIOUSLY USED IN SOUTH KOREA FOR THE MERS OUTBREAK	Preprint
Telles, C. R.	2020	Influence of countries adopted policies for COVID-19 reduction under the view of the airborne transmission framework	Preprint
Teslya, A.	2020	Impact of self-imposed prevention measures and short-term government-imposed social distancing on mitigating and delaying a COVID-19 epidemic: A modelling study	Preprint
Thompson, R. N.	2020	2019-20 Wuhan coronavirus outbreak: Intense surveillance is vital for preventing sustained transmission in new locations	Preprint
Tian, T.	2020	The Effects of Stringent Interventions for Coronavirus Pandemic	Preprint

Tian, T.	2020	Evaluate the timing of resumption of business for the states of New York, New Jersey, and California via a pre-symptomatic and asymptomatic transmission model of COVID-19	Preprint
Tian, T.	2020	Data-driven analysis on the simulations of the spread of COVID-19 under different interventions of China	Preprint
Tiwari, A.	2020	Modelling and analysis of COVID-19 epidemic in India	Preprint
Tobias, A.	2020	COVID19-Global: A shiny application to perform a global comparative data visualization for the SARS-CoV-2 epidemic	Preprint
Traini, M. C.	2020	Modelling the epidemic 2019-nCoV event in Italy: a preliminary note	Preprint
Traini, M. C.	2020	A study of SARS-CoV-2 evolution in Italy: from early days to secondary effects after social distancing	Preprint
Truelove, S. A.	2020	The Potential Impact of COVID-19 in Refugee Camps in Bangladesh and Beyond: a modeling study	Preprint
Tuite, A.	2020	Mathematical modeling of COVID-19 transmission and mitigation strategies in the population of Ontario, Canada	Preprint
Tuomisto, J. T.	2020	An agent-based epidemic model REINA for COVID-19 to identify destructive policies	Preprint
Turk, P. J.	2020	Modeling COVID-19 latent prevalence to assess a public health intervention at a state and regional scale	Preprint
Ucar, A.	2020	Nowcasting and Forecasting the Spread of COVID-19 and Healthcare Demand In Turkey, A Modelling Study	Preprint
Udomsamuthirun, P.	2020	The reproductive index from SEIR model of Covid-19 epidemic in Asean	Preprint
Unlu, E.	2020	Epidemic analysis of COVID-19 Outbreak and Counter-Measures in France	Preprint
Utsunomiya, Y. T.	2020	Growth rate and acceleration analysis of the COVID-19 pandemic reveals the effect of public health measures in real time	Preprint
van Bunnik, B. A. D.	2020	Segmentation and shielding of the most vulnerable members of the population as elements of an exit strategy from COVID-19 lockdown	Preprint
van Wees, J. -D.	2020	Performance of progressive and adaptive COVID-19 exit strategies: a stress test analysis for managing intensive care unit rates	Preprint
van Zandvoort, K.	2020	Response strategies for COVID-19 epidemics in African settings: a mathematical modelling study	Preprint
Vanella, P.	2020	The role of age distribution, time lag between reporting and death and healthcare system capacity on case fatality estimates of COVID-19	Preprint
Vasconcelos, G. L.	2020	Modelling fatality curves of COVID-19 and the effectiveness of intervention strategies	Preprint
Verma, V. R.	2020	Projecting Demand-Supply Gap of Hospital Capacity in India in the face of COVID-19 pandemic using Age-Structured Deterministic SEIR model	Preprint
Victor, A. O.	2020	MATHEMATICAL PREDICTIONS FOR COVID-19 AS A GLOBAL PANDEMIC	Preprint
Victor, A. O.	2020	Estimation of the probability of reinfection with COVID-19 coronavirus by the SEIRUS model	Preprint
Vieira, A.	2020	Rapid estimation of excess mortality in times of COVID-19 in Portugal - Beyond reported deaths	Preprint
Vollmer, M. A. C.	2020	A sub-national analysis of the rate of transmission of COVID-19 in Italy	Preprint
Vyasarayani, C. P.	2020	New approximations, and policy implications, from a delayed dynamic model of a fast pandemic	Preprint
Wahid, A.	2020	The Epidemiology of COVID-19 and applying Non Pharmaceutical interventions by using the Susceptible, Infectious Recovered epidemiological Model in Pakistan	Preprint
Wan, H.	2020	Risk estimation and prediction by modeling the transmission of the novel coronavirus (COVID-19) in mainland China excluding Hubei province	Preprint
Wang, B.	2020	An effect assessment of Airborne particulate matter pollution on COVID-19: A multi-city Study in China	Preprint
Wang, H.	2020	Tracking and forecasting milepost moments of the epidemic in the early-outbreak: framework and applications to the COVID-19	Preprint
Wang, H.	2020	Phase adjusted estimation of the number of 2019 novel coronavirus cases in Wuhan, China	Preprint
Wang, M.	2020	A deterministic epidemic model for the emergence of COVID-19 in China	Preprint
Wang, Q.	2020	Survival-Convolution Models for Predicting COVID-19 Cases and Assessing Effects of Mitigation Strategies	Preprint
Wang, Q.	2020	Effectiveness and cost-effectiveness of public health measures to control COVID-19: a modelling study	Preprint
Wang, X.	2020	Impact of Social Distancing Measures on COVID-19 Healthcare Demand in Central Texas	Preprint
Wang, Y.	2020	Strongly heterogeneous transmission of COVID-19 in mainland China: local and regional variation	Preprint
Warne, D. J.	2020	Hindsight is 2020 vision: Characterisation of the global response to the COVID-19 pandemic	Preprint
Webb, G. F.	2020	A model to predict COVID-19 epidemics with applications to South Korea, Italy, and Spain	Preprint
Wei, C.	2020	The focus and timing of COVID-19 pandemic control measures under healthcare resource constraints	Preprint
Weiss, A.	2020	Spatial and temporal dynamics of SARS-CoV-2 in COVID-19 patients: A systematic review	Preprint
Weitz, J. S.	2020	Intervention Serology and Interaction Substitution: Modeling the Role of 'Shield Immunity' in Reducing COVID-19 Epidemic Spread	Preprint
Weitz, J. S.	2020	Moving Beyond a Peak Mentality: Plateaus, Shoulders, Oscillations and Other 'Anomalous' Behavior-Driven Shapes in COVID-19 Outbreaks	Preprint
Welling, A. A.	2020	Multilevel Integrated Model with a Novel Systems Approach (MIMANSA) for Simulating the Spread of COVID-19	Preprint
Wells, K.	2020	COVID-19 containment policies through time may cost more lives at metapopulation level	Preprint
Wenbao, W.	2020	Transmission dynamics of SARS-COV-2 in China: impact of public health interventions	Preprint
Whittle, R. S.	2020	An ecological study of socioeconomic predictors in detection of COVID-19 cases across neighborhoods in New York City	Preprint
Wickramaarachchi, T.	2020	COVID-19 epidemic in Sri Lanka: A mathematical and computational modelling approach to control	Preprint
Wieland, T.	2020	Flatten the Curve! Modeling SARS-CoV-2/COVID-19 Growth in Germany on the County Level	Preprint
Wijaya, K. P.	2020	A COVID-19 epidemic model integrating direct and fomite transmission as well as household structure	Preprint
Willem, L.	2020	SOCRATES: An online tool leveraging a social contact data sharing initiative to assess mitigation strategies for COVID-19	Preprint
Wilson, N.	2020	When Can Elimination of SARS-CoV-2 Infection be Assumed? Simulation Modelling in a Case Study Island Nation	Preprint
Wilson, N.	2020	Detecting the Emergent or Re-Emergent COVID-19 Pandemic in a Country: Modelling Study of Combined Primary Care and Hospital Surveillance	Preprint
Wilson, N.	2020	Modelling the Potential Health Impact of the COVID-19 Pandemic on a Hypothetical European Country	Preprint
Wood, R. M.	2020	COVID-19 scenario modelling for the mitigation of capacity-dependent deaths in intensive care: computer simulation study	Preprint

Woody, S.	2020	Projections for first-wave COVID-19 deaths across the US using social-distancing measures derived from mobile phones	Preprint
Worby, C. J.	2020	Face mask use in the general population and optimal resource allocation during the COVID-19 pandemic	Preprint
Wu, K.	2020	Generalized logistic growth modeling of the COVID-19 outbreak in 29 provinces in China and in the rest of the world	Preprint
Wu, S. L.	2020	Substantial underestimation of SARS-CoV-2 infection in the United States due to incomplete testing and imperfect test accuracy	Preprint
Wu, S. S.	2020	Epidemiological Development of Novel Coronavirus Pneumonia in China and Its Forecast	Preprint
Xiao, W.	2020	A Cybernetics-based Dynamic Infection Model for Analyzing SARS-COV-2 Infection Stability and Predicting Uncontrollable Risks	Preprint
Xie, Q.	2020	Effect of large-scale testing platform in prevention and control of the COVID-19 pandemic: an empirical study with a novel numerical model	Preprint
Xiong, H.	2020	Simulating the infected population and spread trend of 2019-nCov under different policy by EIR model	Preprint
Xu, C.	2020	Forecast analysis of the epidemics trend of COVID-19 in the United States by a generalized fractional-order SEIR model	Preprint
Xu, L.	2020	Highland of COVID-19 outside Hubei: epidemic characteristics, control and projections of Wenzhou, China	Preprint
Yadav, R. S.	2020	Mathematical Modeling and Simulation of SIR Model for COVID-2019 Epidemic Outbreak: A Case Study of India	Preprint
Yafia, R.	2020	Modeling and Dynamics in Epidemiology, COVID19 with Lockdown and Isolation Effect: Application to Moroccan Case	Preprint
Yamana, T.	2020	Projection of COVID-19 Cases and Deaths in the US as Individual States Re-open May 4,2020	Preprint
Yang, H. M.	2020	Mathematical model describing CoViD-19 in Sao Paulo State, Brazil - Evaluating isolation as control mechanism and forecasting epidemiological scenarios of release	Preprint
Yang, P.	2020	Feasibility of Controlling COVID-19 Outbreaks in the UK by Rolling Interventions	Preprint
Yang, P.	2020	The effect of multiple interventions to balance healthcare demand for controlling COVID-19 outbreaks: a modelling study	Preprint
Yang, P.	2020	Feasibility Study of Mitigation and Suppression Intervention Strategies for Controlling COVID-19 Outbreaks in London and Wuhan	Preprint
Yang, Q.	2020	Short-term forecasts and long-term mitigation evaluations for the COVID-19 epidemic in Hubei Province, China	Preprint
Yang, W.	2020	Rational evaluation of various epidemic models based on the COVID-19 data of China	Preprint
Yap, W. A.	2020	Time-variant strategies for optimizing the performance of non-pharmaceutical interventions (NPIs) in protecting lives and livelihoods during the COVID-19 pandemic	Preprint
Yeo, Y. Y.	2020	A Computational Model for Estimating the Progression of COVID-19 Cases in the US West and East Coasts	Preprint
Yin, H.	2020	Impacts of Early Interventions on the Age-Specific Incidence of COVID-19 in New York, Los Angeles, Daegu and Nairobi	Preprint
You, C.	2020	Estimation of the Time-Varying Reproduction Number of COVID-19 Outbreak in China	Preprint
Yu, X.	2020	Modeling Return of the Epidemic: Impact of Population Structure, Asymptomatic Infection, Case Importation and Personal Contacts	Preprint
Yuan, G. X.	2020	The Prediction for the Outbreak of COVID-19 for 15 States in USA by Using Turning Phase Concepts as of April 10, 2020	Preprint
Yuan, G. X.	2020	The Framework for the Prediction of the Critical Turning Period for Outbreak of COVID-19 Spread in China based on the iSEIR Model	Preprint
Yuan, H.-Y.	2020	The importance of the timing of quarantine measures before symptom onset to prevent COVID-19 outbreaks - illustrated by Hong Kong's intervention model	Preprint
Yuan, H.-Y.	2020	Estimating the risk on outbreak spreading of 2019-nCoV in China using transportation data	Preprint
Yuan, H.-Y.	2020	Effectiveness of quarantine measure on transmission dynamics of COVID-19 in Hong Kong	Preprint
Yyadav, S.	2020	Basic Reproduction Rate and Case Fatality Rate of COVID-19: Application of Meta-analysis	Preprint
Zahiri, A.	2020	Prediction of Peak and Termination of Novel Coronavirus Covid-19 Epidemic in Iran	Preprint
Zhai, Z.-M.	2020	State-by-State prediction of likely COVID-19 scenarios in the United States and assessment of the role of testing and control measures	Preprint
Zhan, C.	2020	Modeling and Prediction of the 2019 Coronavirus Disease Spreading in China Incorporating Human Migration Data	Preprint
Zhan, C.	2020	General Model for COVID-19 Spreading with Consideration of Intercity Migration, Insufficient Testing and Active Intervention: Application to Study of Pandemic Progression in Japa and USA	Preprint
Zhan, C.	2020	Prediction of COVID-19 Spreading Profiles in South Korea, Italy and Iran by Data-Driven Coding	Preprint
Zhang, B.	2020	Study on SARS-COV-2 transmission and the effects of control measures in China	Preprint
Zhang, C.	2020	Pathways of the COVID-19 Pandemic with Human Mobility across Countries	Preprint
Zhang, F.	2020	A simple ecological model captures the transmission pattern of the coronavirus COVID-19 outbreak in China	Preprint
Zhang, F.	2020	Exponential damping: The key to successful containment of COVID-19	Preprint
Zhang, H.	2020	Dynamic Estimation of Epidemiological Parameters of COVID-19 Outbreak and Effects of Interventions on Its Spread	Preprint
Zhang, J.	2020	Evolving epidemiology of novel coronavirus diseases 2019 and possible interruption of local transmission outside Hubei Province in China: a descriptive and modeling study	Preprint
Zhang, K. K.	2020	Characterizing the transmission and identifying the control strategy for COVID-19 through epidemiological modeling	Preprint
Zhang, L.	2020	What is required to prevent a second major outbreak of the novel coronavirus SARS-CoV-2 upon lifting the metropolitan-wide quarantine of Wuhan city, China	Preprint
Zhang, T.	2020	A model to estimate bed demand for COVID-19 related hospitalization	Preprint
Zhang, Y.	2020	Strategic release of lockdowns in a COVID infection model	Preprint
Zhang, Y.	2020	Prediction of the COVID-19 outbreak based on a realistic stochastic model	Preprint
Zhang, Y.	2020	Periodic COVID-19 Testing in Emergency Department Staff	Preprint
Zhang, Y.	2020	Estimating Preventable COVID19 Infections Related to Elective Outpatient Surgery in Washington State: A Quantitative Model	Preprint
Zhang, Y.	2020	The impact of social distancing and epicenter lockdown on the COVID-19 epidemic in mainland China: A data-driven SEIQR model study	Preprint
Zhang, Z.	2020	Prevent the resurgence of infectious disease with asymptomatic carriers	Preprint
Zhao, P. J.	2020	A Social Network Model of the COVID-19 Pandemic	Preprint
Zhao, Q.	2020	Analysis of the epidemic growth of the early 2019-nCoV outbreak using internationally confirmed cases	Preprint

Zhao, S.	2020	Preliminary estimation of the basic reproduction number of novel coronavirus (2019-nCoV) in China, from 2019 to 2020: A data-driven analysis in the early phase of the outbreak	Preprint
Zhao, S.	2020	Modeling the Epidemic Dynamics and Control of COVID-19 Outbreak in China	Preprint
Zhao, Z.	2020	A mathematical model for estimating the age-specific transmissibility of a novel coronavirus	Preprint
Zhao, Z.	2020	The Prediction for Development of COVID-19 in Global Major Epidemic Areas Through Empirical Trends in China by Utilizing State Transition Matrix Model	Preprint
Zhigljavsky, A.	2020	A prototype for decision support tool to help decision-makers with the strategy of handling the COVID-19 UK epidemic	Preprint
Zhigljavsky, A.	2020	Generic probabilistic modelling and non-homogeneity issues for the UK epidemic of COVID-19	Preprint
Zhigljavsky, A.	2020	Comparison of different exit scenarios from the lock-down for COVID-19 epidemic in the UK and assessing uncertainty of the predictions	Preprint
Zhou, C.	2020	Evaluating new evidence in the early dynamics of the novel coronavirus COVID-19 outbreak in Wuhan, China with real time domestic traffic and potential asymptomatic transmissions	Preprint
Zhou, G.	2020	A model simulation study on effects of intervention measures in Wuhan COVID-19 epidemic	Preprint
Zhou, H.	2020	Healthcare-resource-adjusted vulnerabilities towards the 2019-nCoV epidemic across China	Preprint
Zhou, X.	2020	Forecasting the Worldwide Spread of COVID-19 based on Logistic Model and SEIR Model	Preprint
Zhou, X.	2020	Modelling-based evaluation of the effect of quarantine control by the Chinese government in the coronavirus disease 2019 outbreak	Preprint
Zhu, H.	2020	Transmission Dynamics and Control Methodology of COVID-19: a Modeling Study	Preprint
Zhu, X.	2020	Spatially Explicit Modeling of 2019-nCoV Epidemic Trend based on Mobile Phone Data in Mainland China	Preprint
Zhuang, Z.	2020	Estimation of local novel coronavirus (COVID-19) cases in Wuhan, China from off-site reported cases and population flow data from different sources	Preprint
Zia, K.	2020	COVID-19 Outbreak in Oman: Model-Driven Impact Analysis and Challenges	Preprint
Zou, D.	2020	Epidemic Model Guided Machine Learning for COVID-19 Forecasts in the United States	Preprint
Zou, Y.	2020	Outbreak analysis with a logistic growth model shows COVID-19 suppression dynamics in China	Preprint

*At the time of the PubMed, Embase, Web of Science, IEEE Xplore, and ACM Guide to Computing Literature database searches (May 4, 2020).

Supplementary References

1. Wan, K. et al. When will the battle against novel coronavirus end in Wuhan: A SEIR modeling analysis. *J. Glob. Health* https://doi.org/10.7189/jogh.10.011002 (2020).
2. An der Heiden, M. et al. Breaking the waves: modelling the potential impact of public health measures to defer the epidemic peak of novel influenza A/H1N1. *PLoS One* https://doi.org/10.1371/journal.pone.0008356 (2009).
3. Peirlinck, M. et al. Visualizing the invisible: The effect of asymptomatic transmission on the outbreak dynamics of COVID-19. *Comput. Methods Appl. Mech. Eng.* https://doi.org/10.1101/2020.05.23.20111419 (2020).
4. Tan, X., Yuan, L., Zhou, J., Zheng, Y. & Yang, F. Modeling the initial transmission dynamics of influenza A H1N1 in Guangdong Province, China. *Int. J. Infect. Dis.* **17**, e479-484 (2013).
5. House, T. et al. Estimation of outbreak severity and transmissibility: Influenza A(H1N1) pdm09 in households. *BMC Med.* https://doi.org/10.1186/1741-7015-10-117 (2012).
6. Kumar, A. Modeling geographical spread of COVID-19 in India using network-based approach. Preprint at https://www.medrxiv.org/content/10.1101/2020.04.23.20076489v1 (2020).
7. Lesniewski, A. Estimating population immunity without serological testing. Preprint at https://www.medrxiv.org/content/10.1101/2020.04.23.20076786v1 (2020).
8. Distante, C., Piscitelli, P. & Miani, A. Covid-19 Outbreak Progression in Italian Regions: Approaching the Peak by the End of March in Northern Italy and First Week of April in Southern Italy. *Int. J. Environ. Res. Public Health* https://doi.org/10.3390/ijerph17093025 (2020).
9. McRae, M. P. et al. Clinical decision support tool and rapid point-of-care platform for determining disease severity in patients with COVID-19. *Lab Chip* **20**, 2075-2085 (2020).

10. Dorigatti, I., Cauchemez, S., Pugliese, A. & Ferguson, N. M. A new approach to characterising infectious disease transmission dynamics from sentinel surveillance: Application to the Italian 2009-2010 A/H1N1 influenza pandemic. *Epidemics* **4**, 9-21 (2012).

11. Anastassopoulou, C., Russo, L., Tsakris, A. & Siettos, C. Data-based analysis, modelling and forecasting of the COVID-19 outbreak. *PloS One* https://doi.org/10.1371/journal.pone.0230405 (2020).

12. Cori, A. et al. Estimating influenza latency and infectious period durations using viral excretion data. *Epidemics* **4**, 132-138 (2012).

13. Ivorra, B., Ferrández, M. R., Vela-Pérez, M. & Ramos, A. M. Mathematical modeling of the spread of the coronavirus disease 2019 (COVID-19) taking into account the undetected infections. The case of China. *Commun. Nonlinear Sci. Numer. Simul.* https://doi.org/10.1016/j.cnsns.2020.105303 (2020).

14. Cooley, P. et al. Protecting health care workers: a pandemic simulation based on Allegheny County. *Influenza Other Respir. Viruses* **4**, 61-72 (2010).

15. Lofgren, E. et al. The Epidemiological Implications of Incarceration Dynamics in Jails for Community, Corrections Officer, and Incarcerated Population Risks from COVID-19. Preprint at https://www.medrxiv.org/content/10.1101/2020.04.08.20058842v2 (2020).

16. Lewer, D. et al. COVID-19 and homelessness in England: a modelling study of the COVID-19 pandemic among people experiencing homelessness, and the impact of a residential intervention to isolate vulnerable people and care for people with symptoms. Preprint at https://www.medrxiv.org/content/10.1101/2020.05.04.20079301v1 (2020).

17. Earnest, A., Chen, M. I., Ng, D. & Sin, L. Y. Using autoregressive integrated moving average (ARIMA) models to predict and monitor the number of beds occupied during a SARS outbreak in a tertiary hospital in Singapore. *BMC Health Serv. Res.* https://doi.org/10.1186/1472-6963-5-36 (2005).

18. Weissman, G. E. et al. Locally Informed Simulation to Predict Hospital Capacity Needs During the COVID-19 Pandemic. *Ann. Intern. Med.* **173**, 21-28 (2020).

19. Cui, J., Zhang, Y., Feng, Z. L., Guo, S. B. & Zhang, Y. Influence of asymptomatic infections for the effectiveness of facemasks during pandemic influenza. *Math Biosci. Eng.* **16**, 3936-3946 (2019).

20. Liu, M., Zhang, Z. & Zhang, D. A dynamic allocation model for medical resources in the control of influenza diffusion. *J. Syst. Sci. Syst. Eng.* **24**, 276-292 (2015).

21. See, B. D., Liu, S., Lu, Y. & Pang, Q. Staffing a Pandemic Urgent Care facility during an outbreak of pandemic influenza. In *Proceedings of the 2009 Winter Simulation Conference (WSC)* https://doi.org/10.1109/WSC.2009.5429695 (2009).

22. Gatto, M. et al. Spread and dynamics of the COVID-19 epidemic in Italy: Effects of emergency containment measures. *Proc. Natl. Acad. Sci. U. S. A.* **117**, 10484-10491 (2020).

23. Mao, L. Cost-effectiveness of workplace closure and travel restriction for mitigating influenza outbreaks: a network-based simulation. In *Proceedings of the Second ACM SIGSPATIAL International Workshop on the Use of GIS in Public Health* https://doi.org/10.1145/2535708.2535709 (2013).

24. Kretzschmar, M. E., Rozhnova, G. & van Boven, M. Isolation and contact tracing can tip the scale to containment of COVID-19 in populations with social distancing. *Front. Phys.* https://doi.org/10.3389/fphy.2020.622485 (2021).

25. Worby, C. J. & Chang, H. H. Face mask use in the general population and optimal resource allocation during the COVID-19 pandemic. *Nat. Commun.* https://doi.org/10.1038/s41467-020-17922-x (2020).

26. Brauer, M., Zhao, J. T., Bennitt, F. B. & Stanaway, J. D. Global access to handwashing: Implications for COVID-19 control in low-income countries. *Environ. Health Perspect.* https://doi.org/10.1289/EHP7200 (2020).

27. Cleary, B. et al. Using viral load and epidemic dynamics to optimize pooled testing in resource-constrained settings. *Sci. Transl. Med.* https://doi.org/10.1126/scitranslmed.abf1568 (2021).

28. Chin, E. T. et al. Frequency of routine testing for Coronavirus Disease 2019 (COVID-19) in high-risk healthcare environments to reduce outbreaks. *Clin. Infect. Dis.* https://doi.org/10.1093/cid/ciaa1383 (2020).

29. Sedov, L., Krasnochub, A. & Polishchuk, V. Modeling quarantine during epidemics and mass-testing using drones. *PLoS One* https://doi.org/10.1371/journal.pone.0235307 (2020).

30. Zhang, Y. & Cheng, S.-R. Evaluating the need for routine COVID-19 testing of Emergency Department staff: Quantitative analysis. *JMIR Public Health Surveill.* https://doi.org/10.2196/20260 (2020).

31. Nuno, M., Reichert, T. A., Chowell, G. & Gumel, A. B. Protecting residential care facilities from pandemic influenza. *Proc. Natl. Acad. Sci. U. S. A.* **105**, 10625-10630 (2008).

32. Adekunle, A., Meehan, M., Rojas-Alvarez, D., Trauer, J. & McBryde, E. Delaying the COVID-19 epidemic in Australia: evaluating the effectiveness of international travel bans. *Aust. N. Z. J. Public Health* **44**, 257-259 (2020).

33. Quilty, B. J., Clifford, S., CMMID nCoV working group, Flasche, S. & Eggo, R. M. Effectiveness of airport screening at detecting travellers infected with novel coronavirus (2019-nCoV). *Euro. Surveill.* https://doi.org/10.2807/1560-7917.ES.2020.25.5.2000080 (2020).

34. Chong, K. C. & Ying Zee, B. C. Modeling the impact of air, sea, and land travel restrictions supplemented by other interventions on the emergence of a new influenza pandemic virus. *BMC Infect. Dis.* https://doi.org/10.1186/1471-2334-12-309 (2012).

35. Kim, S., Kim, Y. J., Peck, K. R. & Jung, E. School opening delay effect on transmission dynamics of Coronavirus Disease 2019 in Korea: Based on mathematical modeling and simulation study. *J. Korean Med. Sci.* https://doi.org/10.3346/jkms.2020.35.e143 (2020).

36. Chen, S.-C. & Liao, C.-M. Modelling control measures to reduce the impact of pandemic influenza among schoolchildren. *Epidemiol. Infect.* **136**, 1035-1045 (2008).

37. Yan, Q., Tang, S., Gabriele, S. & Wu, J. Media coverage and hospital notifications: Correlation analysis and optimal media impact duration to manage a pandemic. *J. Theor. Biol.* **390**, 1-13 (2016).
38. Buonomo, B. Effects of information-dependent vaccination behavior on coronavirus outbreak: insights from a SIRI model. *Ric. di Mat.* **69**, 483-499 (2020).
39. Gao, X. L., Li, Y. G. & Leung, G. M. Ventilation control of indoor transmission of airborne diseases in an urban community. *Indoor Built Environ.* **18**, 205-218 (2009).
40. German, R., Djanatliev, A., Maile, L., Bazan, P. & Hackstein, H. Modeling Exit Strategies from COVID-19 Lockdown with a Focus on Antibody Tests. Preprint at https://www.medrxiv.org/content/10.1101/2020.04.14.20063750v1 (2020).
41. Mizumoto, K. & Chowell, G. Transmission potential of the novel coronavirus (COVID-19) onboard the diamond Princess Cruises Ship, 2020. *Infect. Dis. Model.* **5**, 264-270 (2020).
42. Laguzet, L. & Turinici, G. Individual Vaccination as Nash Equilibrium in a SIR Model with Application to the 2009-2010 Influenza A (H1N1) Epidemic in France. *Bull. Math. Biol.* **77**, 1955-1984 (2015).
43. Cruz-Aponte, M., McKiernan, E. C. & Herrera-Valdez, M. A. Mitigating effects of vaccination on influenza outbreaks given constraints in stockpile size and daily administration capacity. *BMC Infect. Dis.* https://doi.org/10.1186/1471-2334-11-207 (2011).
44. Beeler, M. F., Aleman, D. M. & Carter, M. W. Estimation and management of pandemic influenza transmission risk at mass immunization clinics. In *Proceedings of the 2011 Winter Simulation Conference (WSC)* https://doi.org/10.1109/WSC.2011.6147834 (2011).
45. Ekici, A., Keskinocak, P. & Swann, J. L. Pandemic influenza response. In *2008 Winter Simulation Conference* https://doi.org/10.1109/WSC.2008.4736242 (2008).
46. Zhao, S. et al. Preliminary estimation of the basic reproduction number of novel coronavirus (2019-nCoV) in China, from 2019 to 2020: A data-driven analysis in the early phase of the outbreak. *Int. J. Infect. Dis.* **92**, 214-217 (2020).

47. Johns Hopkins University Center for Systems Science and Engineering. COVID-19 Dashboard. https://coronavirus.jhu.edu/map.html (2020).

48. Worldometers.info. COVID-19 Coronavirus Pandemic. https://www.worldometers.info/coronavirus/ (2020).

49. Yu, Z., Liu, J. & Zhu, X. Inferring a district-based hierarchical structure of social contacts from census data. *PloS One* https://doi.org/10.1371/journal.pone.0118085 (2015).

50. Adegboye, O. A., Adekunle, A. I. & Gayawan, E. Early transmission dynamics of novel Coronavirus (COVID-19) in Nigeria. *Int. J. Environ. Res. Public Health* https://doi.org/10.3390/ijerph17093054 (2020).

51. Wallace, D. I., Chen, M. & Mondaini, R. P. A simulation of the US influenza outbreak in 2009-2010 using a patch SIR model based on airport transportation data. In *Biomat 2013: International Symposium on Mathematical and Computational Biology* https://doi.org/10.1142/9789814602228_0017 (2014).

52. Riley, P. et al. Multiple estimates of transmissibility for the 2009 influenza pandemic based on influenza-like-illness data from small US military populations. *PLoS Comput. Biol.* https://doi.org/10.1371/journal.pcbi.1003064 (2013).

53. Furushima, D., Kawano, S., Ohno, Y. & Kakehashi, M. Estimation of the basic reproduction number of novel Influenza A (H1N1) pdm09 in elementary schools using the SIR model. *Open Nurs. J.* **11**, 64-72 (2017).

54. Fenichel, E. P., Berry, K., Bayham, J. & Gonsalves, G. A cell phone data driven time use analysis of the COVID-19 epidemic. Preprint at https://www.medrxiv.org/content/10.1101/2020.04.20.20073098v1 (2020).

55. Que, J. & Tsui, F. C. Spatial and temporal algorithm evaluation for detecting over-the-counter thermometer sale increases during 2009 H1N1 pandemic. *Online J. Public Health Inform.* https://doi.org/10.5210/ojphi.v4i1.3915 (2012).

56. Bryant, P. & Elofsson, A. Estimating the impact of mobility patterns on COVID-19 infection rates in 11 European countries. *PeerJ* https://doi.org/10.7717/peerj.9879 (2020).

57. Zhou, X. et al. A spatial-temporal method to detect global influenza epidemics using heterogeneous data collected from the internet. *IEEE/ACM Trans. Comput. Biol. Bioinform.* **15**, 802-812 (2018).

58. Dukic, V., Lopes, H. F. & Polson, N. G. Tracking Epidemics with Google Flu Trends Data and a State-Space SEIR Model. *J. Am. Stat. Assoc.* **107**, 1410-1426 (2012).

59. Miller, A. C. et al. Mobility trends provide a leading indicator of changes in SARS-CoV-2 transmission. Preprint at https://www.medrxiv.org/content/10.1101/2020.05.07.20094441v1 (2020).

60. Rocklöv, J., Sjödin, H. & Wilder-Smith, A. COVID-19 outbreak on the Diamond Princess cruise ship: estimating the epidemic potential and effectiveness of public health countermeasures. *J. Travel Med.* https://doi.org/10.1093/jtm/taaa030 (2020).

61. Emery, J. C. et al. The contribution of asymptomatic SARS-CoV-2 infections to transmission on the Diamond Princess cruise ship. *eLife* https://doi.org/10.7554/eLife.58699 (2020).

62. Xiao, H. et al. Influence of extreme weather and meteorological anomalies on outbreaks of influenza A (H1N1). *Chin. Sci. Bull.* **58**, 741-749 (2013).

63. Qiu, Y., Chen, X. & Shi, W. Impacts of social and economic factors on the transmission of coronavirus disease 2019 (COVID-19) in China. *J. Popul. Econ.* **33**, 1127-1172 (2020).

64. Springborn, M., Chowell, G., MacLachlan, M. & Fenichel, E. P. Accounting for behavioral responses during a flu epidemic using home television viewing. *BMC Infect. Dis.* https://doi.org/10.1186/s12879-014-0691-0 (2015).
